# Optimal control to reach eco-evolutionary stability in metastatic castrate-resistant prostate cancer

**DOI:** 10.1371/journal.pone.0243386

**Published:** 2020-12-08

**Authors:** Jessica Cunningham, Frank Thuijsman, Ralf Peeters, Yannick Viossat, Joel Brown, Robert Gatenby, Kateřina Staňková

**Affiliations:** 1 Department of Integrated Mathematical Oncology, Moffitt Cancer Center & Research Institute, Tampa, Florida, United States of America; 2 Department of Data Science and Knowledge Engineering, Maastricht University, Maastricht, The Netherlands; 3 CEREMADE, Université Paris-Dauphine, Université PSL, Paris, France; 4 Department of Biological Sciences, University of Illinois at Chicago, Chicago, Illinois, United States of America; 5 Department of Diagnostic Imaging and Interventional Radiology, Moffitt Cancer Center & Research Institute, Tampa, Florida, United States of America; 6 Delft Institute of Applied Mathematics, Delft University of Technology, Delft, The Netherlands; University of Southern California, UNITED STATES

## Abstract

In the absence of curative therapies, treatment of metastatic castrate-resistant prostate cancer (mCRPC) using currently available drugs can be improved by integrating evolutionary principles that govern proliferation of resistant subpopulations into current treatment protocols. Here we develop what is coined as an ‘evolutionary stable therapy’, within the context of the mathematical model that has been used to inform the first adaptive therapy clinical trial of mCRPC. The objective of this therapy is to maintain a stable polymorphic tumor heterogeneity of sensitive and resistant cells to therapy in order to prolong treatment efficacy and progression free survival. Optimal control analysis shows that an increasing dose titration protocol, a very common clinical dosing process, can achieve tumor stabilization for a wide range of potential initial tumor compositions and volumes. Furthermore, larger tumor volumes may counter intuitively be more likely to be stabilized if sensitive cells dominate the tumor composition at time of initial treatment, suggesting a delay of initial treatment could prove beneficial. While it remains uncertain if metastatic disease in humans has the properties that allow it to be truly stabilized, the benefits of a dose titration protocol warrant additional pre-clinical and clinical investigations.

## 1 Introduction

While overall survival of early stage prostate cancer is increasing due to early detection and improving therapy for local and regionally confined disease, the overall survival for metastatic prostate cancer patients remains bleak [1]. This is largely due to the ability of metastatic cancer populations to evolve resistance to all currently available therapies [2–7]. While the search for truly curative therapies continues, there is some evidence that patient outcomes can be improved using currently available therapies by integrating evolutionary principles that govern proliferation of resistant subpopulations into current treatment protocols [8–10]. Delaying or preventing the evolution of resistance, known as ‘evolutionary’ therapies, could prolong drug sensitivity and potentially allow for large increases in overall survival.

For instance, a type of evolutionary therapy known as adaptive therapy uses drug holidays timed specifically to each patients’ disease dynamics in an attempt to intentionally maintain a sufficient level of drug sensitive cells [8, 11–14]. Upon withdrawing therapy, these sensitive cells can compete with and suppress resistant cancer cells, thus prolonging drug efficacy. Continuous or maximum tolerated dose therapies quickly eliminate the entire sensitive population resulting in treatment failure as resistance cells can now grow unchecked. Adaptive therapy clinical trials are underway in multiple different cancers including trials in metastatic castrate-resistant prostate cancer (NCT02415621, NCT03511196), in melanoma—NCT03543969, and in thyroid—NCT03630120.

The design of these adaptive therapies is rooted heavily in the use of mathematical modeling, more specifically evolutionary game theory (EGT) [15–17], which helps us to model situations where multiple organisms interact and where interactions with individuals of different properties largely determine one’s chances of survival (fitness). Unlike in the classical game theory [18, 19], individuals are not expected to be overtly rational, and their ‘strategies’ are properties that they inherit from their predecessors. The EGT models build and test the fundamental understanding of the dynamical interactions underlying tumor population dynamics [20–25]. The development and study of mathematical models like these has suggested other possible evolutionary therapies beyond adaptive therapies, most notably the notion of long term stabilization [26]. One of the core properties of evolutionary systems that can be studied with EGT is the presence of an evolutionary stable strategy (ESS) [15–17], which corresponds to the stable equilibria of the tumor dynamics [27]. If such stable equilibria in tumors exist, reaching it using available therapies could provide a means for achieving long term stabilization of tumors and subsequent dramatic increase in progression-free survival [28, 29].

Previous theoretical work suggests that stable polymorphic equilibria could exist within tumor subpopulations [30, 31]. Interestingly, early preclinical in-vivo studies of adaptive therapy in OVCAR xenografts treated with carboplatin, and in MDA-MB-231/luc triple-negative and MCF7 estrogen receptor–positive (ER+) breast cancers treated with paclitaxel showed the ability to stabilize tumor volume, though the underlying subpopulations were not explicitly measured [32, 33]. In both of these studies, once initial tumor volume control using the maximum tolerable dose was achieved, it could be maintained with progressively smaller drug doses, suggestive of a stable equilibria. Furthermore, polymorphic stability in heterogeneous tumor cell populations has been shown to exist explicitly in breast cancer and neuroendocrine pancreatic cancer in-vitro [34, 35].

If these stable equilibria exist, the clinically relevant question is how can we use currently available drugs to arrive at these equilibria? The ‘evolutionary stable therapies’ attempt to maintain a stable polymorphic tumor composition of cells sensitive and resistant to therapy, in order to prolong treatment efficacy and progression free survival [36, 37]. Previous mathematical studies have developed examples of evolutionary stable therapies, by focusing only on stabilization of the frequency dynamics, while generally ignoring the density dynamics [38, 39]. Stabilization of only the underlying frequency dynamics is inadequate in the case of long term stabilization of a growing tumor where tumor cell density is paramount to patient health [40].

Here we develop an evolutionary stable therapy for the Zhang et al. mathematical model that was used to inform the adaptive therapy clinical trial in mCRPC [8]. First, stability analysis of the evolutionary game theoretic model of mCRPC allows for identification of basic properties of the model that are required for a stable equilibria to exist within constraints on density. Next, to identify an evolutionary stable therapy, we frame the problem of arriving at a stable equilibrium as an optimal control problem [41–46]. Interestingly, previous optimal control studies with the objective of lengthening patient overall survival identified stabilization techniques as optimal treatment strategies [47, 48]. The evolutionary stable therapy identified here with the explicit objective of reaching a stable equilibria is then translated into a clinically feasible strategy and performance is compared against simulated standard of care and adaptive therapy treatment protocols for >200, 000 virtual patients. The clinical and psychological implications of this new strategy are discussed.

## 2 Metastatic castrate-resistant prostate cancer growth model

We build upon the [8, 49], and [50] mathematical models that consider mCRPC as an evolutionary game between three cancer cell types:

- *T*^+^ cells requiring exogenous androgen;
- *T*^*P*^ cells expressing 17*α*-hydroxy/17,20-lyase (CYP17*α*) and producing testosterone; and
- *T*^−^ cells that are androgen-independent.

With abiraterone therapy, the patients are also on androgen deprivation therapy that suppresses the production of testosterone by the body. This suppression does not directly affect *T*^*P*^ or *T*^−^ cells, but it does mean that *T*^+^ can only exist in the presence of *T*^*P*^ cells because the *T*^*P*^ cells secrete testosterone as a public good that can support the *T*^+^ cells.

### 2.1 Lotka-Volterra model

The system of equations describes the interactions between *T*^+^, *T*^*P*^, and *T*^−^ cell types, i∈T= {*T*^+^, *T*^*P*^, *T*^−^}. The instantaneous rate of change in the population size of each cell type i∈T, ${\dot{x}}_{i}\overset{\text{def}}{=}\frac{d\;x_{i}}{d\;t},$ is given by

$$\begin{array}{c} {\dot{x}}_{i}=r_{i}x_{i}(1-\frac{\sum_{j\in T}\alpha_{ij}x_{j}}{K_{i}}) \end{array}$$ (1)

where the parameters *r*_*i*_, *K*_*i*_, and *α*_*ij*_ correspond to the growth rates, carrying capacities, and competition coefficients, respectively.

### 2.2 Growth rates *r*_*i*_

The growth rates of the three subpopulations in (1) were derived from the measured doubling times of representative cell lines. The LNCaP cell line (ATCC@CRL-1740) is representative of *T*^+^ cells with a measured doubling time of 60 hours. The H295R cell line (ATCC@CRL-2128) is representative of *T*^*P*^ cells with a doubling time of 48 hours. The PC-3 cell line (ATCC®CRL-1435) is representative of *T*^−^ cells with a doubling time of 25 hours. From these doubling times the growth rates of the *T*^+^, *T*^*P*^, and *T*^−^ cells would be 0.27726, 0.34657, and 0.66542, (units of per day) respectively. These cell line derived growth rates are unlikely to be biologically feasible within a tumor environment with limited resources. We therefore scale these growth rates to *r*_*T*^+^_ = 2.7726 ⋅ 10^−3^, *r*_*T*^*P*^_ = 3.4657 ⋅ 10^−3^, and *r*_*T*^−^_ = 6.6542 ⋅ 10^−3^ as in [8]. Note that the intrinsic growth rates do not influence the equilibrium frequency of the three cell types, only the rate at which the dynamics play out.

### 2.3 Carrying capacities *K*_*i*_ and the effect of abiraterone

In our model, the abiraterone dose Λ(*t*) ∈ [0, 1] equals 0 if no drug is given at time *t*, equals to 1 if the maximum tolerated dose is applied, and scales between (0, 1) at intermediate doses. The carrying capacity of *T*^−^ cells is independent of the abiraterone dose and we set it to *K*_*T*^−^_(*t*) = 10000 for all *t*. The actual magnitude of *K*_*T*^−^_ is arbitrary. What matters is how it scales relative to the carrying capacities of the other two cell types. The carrying capacities of *T*^*P*^ and *T*^+^ cells are affected by abiraterone dose. With no abiraterone given, the carrying capacity for *T*^*P*^ cells is 10000, the same as for *T*^−^. We assume that abiraterone directly affects the carrying capacity of *T*^*P*^ and reduces it linearly, to a minimum of 100 when abiraterone is administered at maximum tolerated dose, i.e. when Λ(*t*) = 1. Therefore, as in [50], we assume that *K*_*T*^*P*^_ at time *t* is a linear function of the dose Λ(*t*) as follows:

$$\begin{array}{c} K_{T^{P}}(\Lambda (t))=10000-9900\Lambda \left(t\right) \end{array}$$ (2)

Additionally, abiraterone affects the growth of *T*^+^ cells as the carrying capacity of the *T*^+^ cell population derives entirely from utilizing the endogenous testosterone produced by the *T*^*P*^ cells. We assume that the carrying capacity of *T*^+^ is a linear function of the density of *T*^*P*^ cells as defined by

$$\begin{array}{c} K_{T^{+}}(\Lambda (t))=\mu (\Lambda (t))\cdot x_{T^{P}}\left(t\right) \end{array}$$ (3)

where

$$\begin{array}{c} \mu (\Lambda (t))=1.5-\Lambda \left(t\right). \end{array}$$ (4)

In this way, the per cell contribution of *T*^*P*^ to *K*_*T*^+^_ referred to here as the symbiosis coefficient *μ*(Λ(*t*)), has a maximum of 1.5 when no abiraterone is given and is lowered linearly to a minimum value of 0.5 as abiraterone dose increases to the maximum tolerated dose. When Λ(*t*) = 0 the carrying capacity of *T*^+^ cells could be as high as 15000 if the density of *T*^*P*^ cells was equal to *K*_*T*^*P*^_ = 10000. Since the maximum carrying capacity of any one type of cell type is at least 10000, we must define the maximal tolerated tumor burden (viable total tumor population size) to be less than 10000. This ensures that a tumor burden that is untreated with abiraterone where Λ(*t*) = 0 for all *t* will result in patient death by any one cell type. We choose a relatively high maximal tolerated tumor burden of 9000 because we assume that clinically, patient death does not occur until the latest moment possible, only after the human body has exhausted all of its resources. This results in the following viability constraint:

$$\begin{array}{c} \sum_{i\in T}x_{i}\leq 9000 \end{array}$$ (5)

where $i\in T=\{T^{+},T^{P},T^{-}\}$.

### 2.4 Competition coefficients *α*_*ij*_nd their impact on system stability

The behavior of the model, including stability, depends heavily on the 3 × 3 competition matrix that characterizes the evolutionary game between the three cancer cell types from the set T={*T*^+^, *T*^*P*^, *T*^−^}. Each competition coefficient represents the effect of an individual of type *j* on the growth rate of type *i*. The competition matrix used in [8] and analyzed here is

$$\begin{array}{c} A=\left(\alpha_{ij}\right)=(\begin{array}{ccc} 1 & 0.7 & 0.8\\ 0.4 & 1 & 0.6\\ 0.5 & 0.9 & 1 \end{array}) \end{array}$$ (6)

Stability analysis is performed for different but constant values of Λ(⋅) ∈ [0, 1], as we are interested in situations where tumor burden can be maintained using a fixed amount of medication. A detailed explanation of the original development of this competition matrix and stability analysis is provided in detail in S1 in S1 File. The population densities $x_{T^{+}}^{*}$, $x_{T^{P}}^{*}$ and $x_{T^{-}}^{*},$ corresponding to stable equilibria for matrix (6) are shown in S2 in S1 File. While stable equilibria for (1) exist for this competition matrix, there are no stable equilibria that correspond to a total tumor volume less than the patient viability constraint (5), or even $\sum_{i\in T}x_{i}\leq 10000.$ The stable points are dominated by the resistant *T*^−^ cells, out-competing the *T*^+^ and *T*^*P*^ cells. While some stable points exist where *T*^+^ and *T*^*P*^ cells can contain the *T*^−^ cells, it requires so many *T*^*P*^ cells that the patient viability constraint must be broken.

From analysis in S3 in S1 File, the coefficients *α*_31_ and *α*_32_ describing the competition effect of *T*^+^ and *T*^*P*^ cells on *T*^−^ cells respectively, are the key parameters affecting containment of the *T*^−^ cells. Specifically, *α*_31_ and/or *α*_32_ must be greater than one. While the initial assumptions of the model required the competition coefficients to be within [0, 1], studies co-culturing sensitive and resistant cell lines show that competition coefficients between cancer cells may not be limited to this range. In-vitro and theoretical studies tend to suggest significant competition between cancer cells in non small cell lung cancer and breast cancer cell lines [28, 51, 52]. For example, results from a novel re-imagining of a Gause style experiment using two competing breast cancer cell lines, MCF7 and MB-MDA-231, with analysis using Lotka-Volterra models suggest that the competition coefficients between these cancer cell lines may be as high as 12.6 [34].

While the exact values of these competition coefficients in-vitro or in-vivo is currently unknown, here we consider a formulation of the model where we increase the competition effect of *T*^*P*^ cells on *T*^−^ cells, choosing a value of *α*_32_ = 2. This value is chosen because 1) it is large enough to allow for stable equilibria within the patient viability constraint (5), and 2) is small enough to not eliminate *T*^−^ in the stable equilibria (which is the case for higher values of *α*_32_, such as *α*_32_ = 5, see S3 in S1 File). With *α*_32_ = 2, the resistant *T*^−^ cells are still present in the tumor at stable equilibria, which would be expected clinically. The new matrix is shown below:

$$\begin{array}{c} A=\left(\alpha_{ij}\right)=(\begin{array}{ccc} 1 & 0.7 & 0.8\\ 0.4 & 1 & 0.6\\ 0.5 & 2 & 1 \end{array}) \end{array}$$ (7)

**Fig 1:**
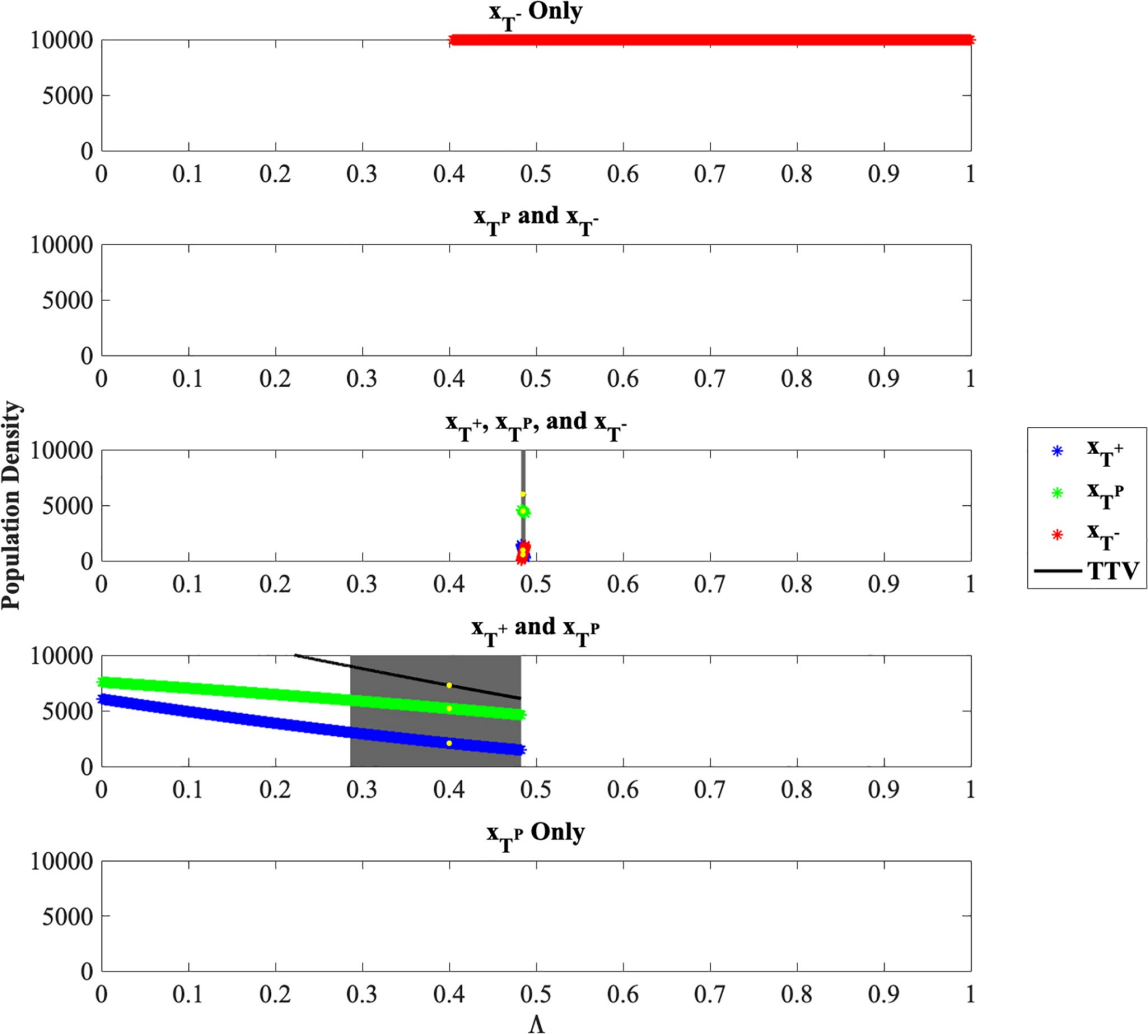
Population densities for stable equilibria. Population densities $x_{T^{+}}^{*}$, $x_{T^{P}}^{*}$ and $x_{T^{-}}^{*},$ corresponding to stable equilibria for Λ ∈ [0, 1] and for *α*_32_ = 2.0. The gray highlighted regions show the stable equilibria that are within the patient viability constraint (5). The yellow highlighted points represent two specific stable equilibria chosen for further analysis.

For the matrix *A*_*ij*_ = (*a*_*ij*_) as defined in (7) the resulting stable equilibria are shown in Fig 1.

From Fig 1, for all values of Λ ≥ 0.4041, $(x_{T^{+}}^{*},x_{T^{P}}^{*},x_{T^{-}}^{*})=\left(0,0,10000\right)$ is a stable equilibrium. There are no stable equilibria for a polymorphic *T*^*P*^ and *T*^−^ tumor nor for the monomorphic *T*^*P*^ tumor. We can see that there is a bifurcation at about Λ = 0.4828: For any smaller Λ, a stable equilibrium contains a mix of *T*^+^ and *T*^*P*^ cells, while for Λ ∈ [0.4828, 0.4877), a stable equilibrium contains a mix of all three cell types. Regions of Λ where the total tumor burden of the stable equilibrium is within the patient viability constraint (5) are highlighted in gray.

## 3 Optimal control to arrive at stable equilibria

If the system is at a stable equilibrium with a constant dose of abiraterone, like those shown above, remaining at that dose will keep the system at that equilibrium indefinitely. However, the clinically relevant question is: Can we arrive at this equilibrium from any viable point *x*(*t*_0_) = (*x*_*T*^+^_, *x*_*T*^*P*^_, *x*_*T*^−^_) corresponding to an incoming patient tumor composition, using only varying doses of abiraterone as the control? We frame the problem of arriving at an equilibrium point as an optimal control problem to identify the dosing schedule $\Lambda^{*}\left(\cdot \right)\overset{\text{def}}{=}{\left[\Lambda^{*}\left(t\right)\right]}_{t\in \left[t_{0},t_{f}\right]}$ that minimizes the average distance between the state of the system *x*(*t*) and the equilibrium point x* over time horizon between the initial time *t*_0_ and the final time *t*_*f*_:

$$\begin{array}{c} \Lambda^{*}\left(\cdot \right)=\text{arg}\min_{\Lambda (\cdot )}\int_{t_{0}}^{t_{f}}\sqrt{{\left(x_{T^{+}}\left(t\right)-x_{T^{+}}^{*}\right)}^{2}+{\left(x_{T^{P}}\left(t\right)-x_{T^{P}}^{*}\right)}^{2}+{\left(x_{T^{-}}\left(t\right)-x_{T^{-}}^{*}\right)}^{2}}dt \end{array}$$ (8)

with respect to the system dynamics (1), growth rates *r*_*T*^+^_ = 2.7726 ⋅ 10^−3^, *r*_*T*^*P*^_ = 3.4657 ⋅ 10^−3^, *r*_*T*^−^_ = 6.6542 ⋅ 10^−3^, carrying capacities for *T*^*P*^ and *T*^+^ given by Eqs (2) and (3), respectively, *K*_*T*^−^_ = 10000, and with *A* = (*α*_*ij*_) defined by (7).

The time horizon is set to 10000 as this is well beyond the lifespan of the typical patient presented with metastatic castrate-resistant prostate cancer (> 20 simulated years under the assigned growth rates). In this way, if the tumor volume remains below the patient viability constraint (5) over this time interval, the patient will most likely die from some other cause.

**Fig 2:**
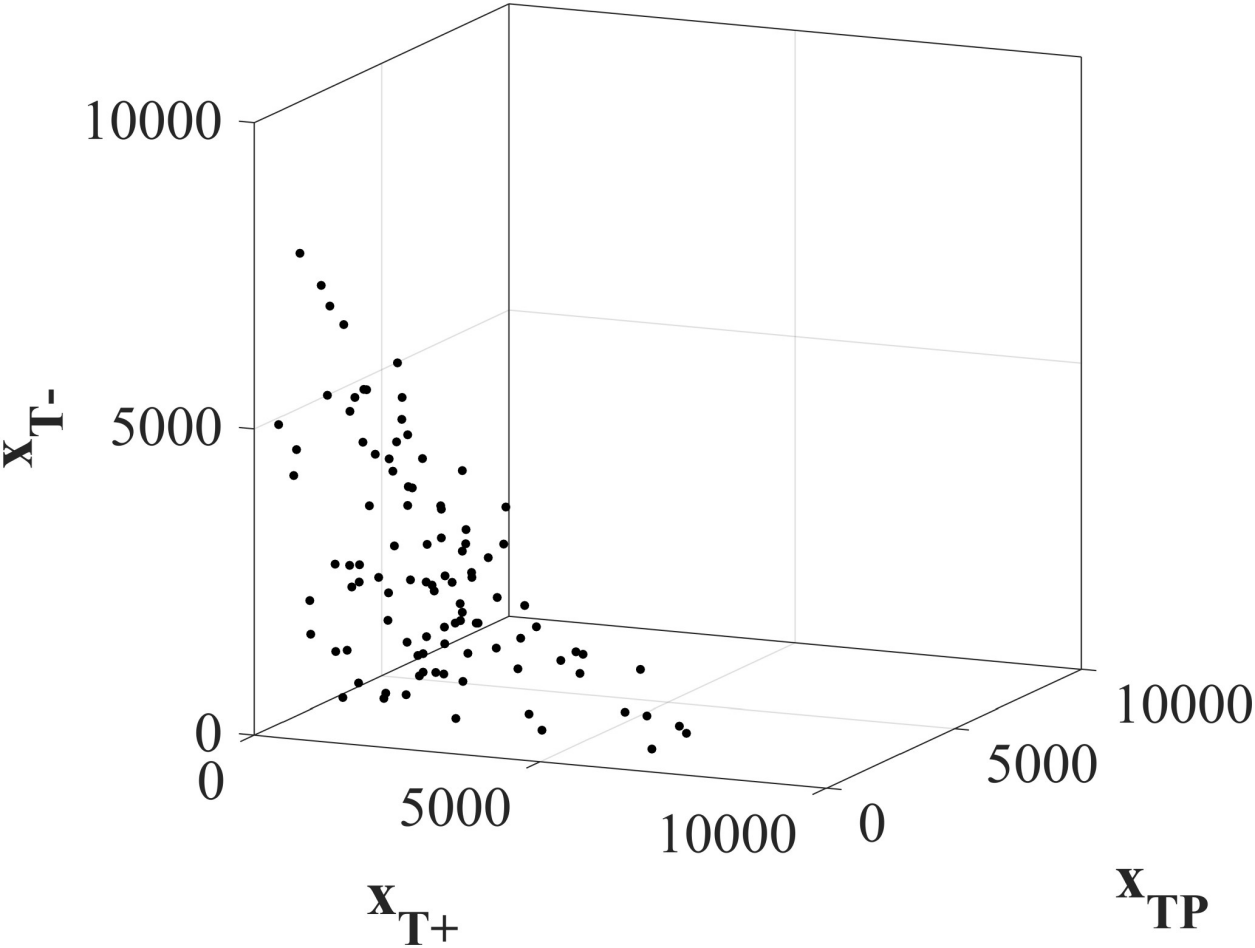
Initial tumor compositions for Forwards Backwards Sweep analysis. 100 randomly selected initial tumor compositions used in the Forwards Backwards Sweep optimal control analysis. All initial total tumor volumes satisfy the patient viability constraint $\sum_{i\in T}x_{i}\left(t_{0}\right)\leq 9000$.

We vary the initial tumor compositions *x*(*t*_0_) = (*x*_*T*^+^_(*t*_0_), *x*_*T*^*P*^_(*t*_0_), *x*_*T*^−^_(*t*_0_)) to explore a wide range of possible initial conditions. 100 randomly selected tumors that satisfy the viability constraint (5) are explored in Fig 2.

We know from Section 2.4 that two regions of stable equilibria in terms of Λ exist. For Λ ∈ [0, 0.4828), the two species *T*^+^ and *T*^*P*^ equilibrium is the stable equilibrium. We select (2082.76, 5206.90, 0.00), corresponding to Λ = 0.4, as x* in (8). For Λ ∈ [0.4828, 0.4877), the three-species equilibrium is a stable equilibrium. We select (863.45, 4436.73, 694.82), corresponding to Λ = 0.4848, as another possible x* in (8). These two points are shown with yellow highlights in Fig 1. While we chose these two equilibria to study specifically for (8), any equilibrium corresponding to Λ ∈ (0.2866, 0.4877) could be used as these equilibria fall within the patient viability constraint. Alternatively, we could adopt a reach-avoid formulation instead of selecting a specific x* in (8).

### 3.1 Forward Backwards Sweep method

Here we use the Forward Backward Sweep (FBS) numerical technique to find the dosing schedule Λ*(⋅) satisfying (8). The FBS method characterizes the optimal control problem using the Hamiltonian formulation. The Hamiltonian for this problem is given below as follows:

$$\begin{array}{cc} H(t) & =\sqrt{{\left(x_{T^{+}}\left(t\right)-x_{T^{+}}^{*}\right)}^{2}+{\left(x_{T^{P}}\left(t\right)-x_{T^{P}}^{*}\right)}^{2}+{\left(x_{T^{-}}\left(t\right)-x_{T^{-}}^{*}\right)}^{2}}\\ & +\lambda_{T^{+}}\left(t\right)x_{T^{+}}\left(t\right)r_{T^{+}}(1-\frac{x_{T^{+}}\left(t\right)+\alpha_{12}x_{T^{P}}\left(t\right)+\alpha_{13}x_{T^{-}}\left(t\right)}{x_{T^{P}}\left(t\right)\left(K_{T^{+}}\left(\Lambda \left(t\right)\right)\right)})\\ & +\lambda_{T^{P}}\left(t\right)x_{T^{P}}\left(t\right)r_{T^{P}}(1-\frac{\alpha_{21}x_{T^{+}}\left(t\right)+x_{T^{P}}\left(t\right)+\alpha_{23}x_{T^{-}}\left(t\right)}{K_{T^{+}}\left(\Lambda \left(t\right)\right)})\\ & +\lambda_{T^{-}}\left(t\right)x_{T^{-}}\left(t\right)r_{T^{-}}(1-\frac{\alpha_{31}x_{T^{+}}\left(t\right)+\alpha_{32}x_{T^{P}}\left(t\right)+x_{T^{-}}\left(t\right)}{K_{T^{-}}}) \end{array}$$ (9)

where λ_*i*_’s are referred to as the costates or adjoint variables, given by $\lambda_{i}=-\frac{\partial H}{\partial x_{i}}$. The state equations given in (1) are subject to the initial conditions (*x*_*T*^+^_(*t*_0_), *x*_*T*^*P*^_(*t*_0_), *x*_*T*^−^_(*t*_0_)) shown in Fig 2 and are solved forwards in time. The costate equation must satisfy a transversality condition λ_*i*_(*t*_*f*_) = 0 and are solved backwards in time, from the final time towards the beginning. A full explanation of FBS is given in [53] and in detail particularly for this system in S4 in S1 File. The solution provided by FBS approximates the treatment strategy Λ*(⋅) that minimizes the Hamiltonian (1), subject to initial conditions for state variables and final conditions for costates, which is equivalent to minimizing (8), subject to the system dynamics (1).

## 4 Optimizing abiraterone treatment to reach stable equilibrium

**Fig 3:**
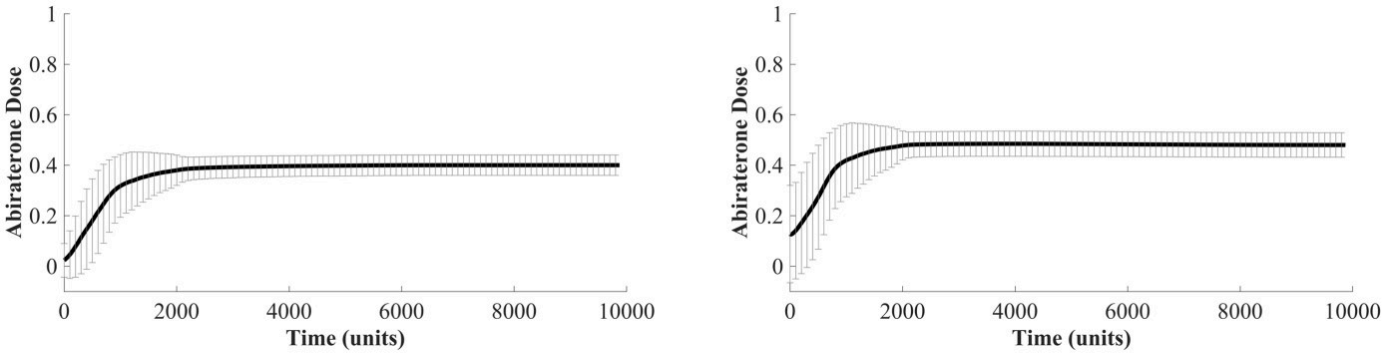
Forwards Backwards Sweep optimized dosing schedules. Forward Backwards Sweep results for optimal dosing schedule to arrive at two-species stability point (left panel) and three species stability point (right panel). The mean of all 100 paths is shown with symmetric one standard deviation error bars (dosage values <0 are not possible). Standard error of the mean is on the order of 10^−3^.

Adopting the Forward Backward Sweep method introduced in the previous section, we identified the optimized abiraterone treatment strategy for each of the 100 initial conditions (*x*_*T*^+^_(*t*_0_), *x*_*T*^*P*^_(*t*_0_), *x*_*T*^−^_(*t*_0_)) shown in Fig 2. While the individual optimized treatment strategies of each of the 100 virtual patients are shown in the S5 in S1 File, Fig 3 shows the mean optimal treatment strategy where the objective is to reach the two-species equilibrium point (2082.76, 5206.90, 0.00), corresponding to Λ = 0.4 (left), and the three-species equilibrium (863.45, 4436.73, 694.82), corresponding to Λ = 0.4848 (right). To reach the two-species equilibrium point, the individual treatment strategies tend towards a Λ(*t*_0_) = 0 while in some cases to reach the three-species equilibrium point a Λ(*t*_0_)>0 is required. Interestingly, the average optimized treatment dose to arrive at either equilibrium point is a simple dose titration scheme that begins with a small abiraterone dose and increases slowly until the known equilibrium dose Λ = 0.4 and Λ = 0.4848, respectively, is reached.

**Fig 4:**
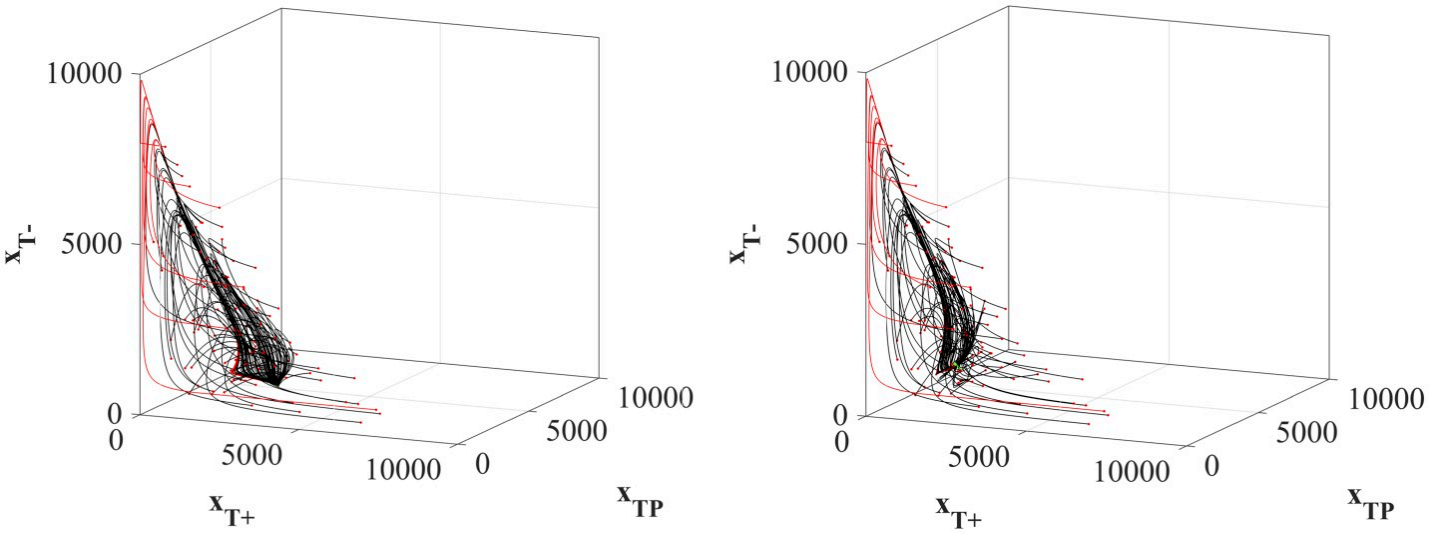
System state trajectories under optimal dose schedules. State trajectories from each of the 100 initial tumor compositions to the two species equilibrium point (left panel) and the three-species equilibrium point (right panel). Paths highlighted in red breach the patient viability constraint before reaching the equilibrium point.

The state trajectory paths *x*(⋅) for each of the 100 initial tumors to the equilibrium with the optimized treatments are shown in Fig 4. It is important to note that while all 100 initial tumors can reach the stable equilibrium, a subset of the trajectories (13 initial tumors) result in patient death by violating the patient viability constraint (5). These trajectories are highlighted in red in both panels. The common characteristic of these initial tumors that cannot be stabilized without first causing patient death is that the initial value of *x*_*T*^*P*^_ is ≤ 4.10% of the total tumor composition (S6 in S1 File). Because both equilibria require a significant amount of *T*^*P*^ cells, if there are very few of them to begin with, the only way to shift the composition of the tumor towards the equilibrium points is to allow for a very high tumor volume that, in this model, results in patient death. Increasing or decreasing the patient viability constraint will either rescue some of these lost patients or cause more of the patients to cross the constraint, respectively.

## 5 Clinical translation of dose titration

Can a dose titration of abiraterone be successfully implemented under clinical constraints to achieve the tumor stabilization or mCRPC? Dose titration is a very common clinical process of incrementally increasing the dose of a medication in order to find the most beneficial dosage and is commonly used to find the appropriate dose to manage other long-term illnesses such as diabetes and depressive disorders [54]. Generally, little information is available to the physician and dose changes are made based on benefits and side effects of the patient in real time. Similarly, in the case of titrating abiraterone, the physician will not know either the location or existence of an equilibrium nor the initial tumor composition. To address this lack of information, we analyze a variety of generalized dose titration schedules that do not require precise initial or final conditions, but instead rely on monitoring the total tumor volume (i.e. PSA measurement) in real time.

In all modeled titration protocols the total tumor volume $V\left(t\right)=\sum_{i\in T}x_{i}\left(t\right)$ is measured every 100 simulated time points (just over 3 months in real time). Since the equilibrium tumor volume V* corresponding to the equilibrium point x* that we want to reach will be unknown in the clinic, here we test two volumes *V*_*a*_ and *V*_*b*_ that can be measured clinically: 1. the incoming baseline tumor volume $V_{a}=\sum_{i\in T}x_{i}\left(t_{0}\right)$, and 2. a maximum tolerable tumor volume defined as a volume just smaller than the volume that causes a loss in quality of life (i.e. bone pain due to extensive metastases). In reality, this volume will vary greatly with age, demographics, general overall health, psychological comfort, and other patient-specific factors. Here, we choose a relatively large maximum tolerable tumor volume *V*_*b*_ = 7000 for all patients.

**Fig 5:**
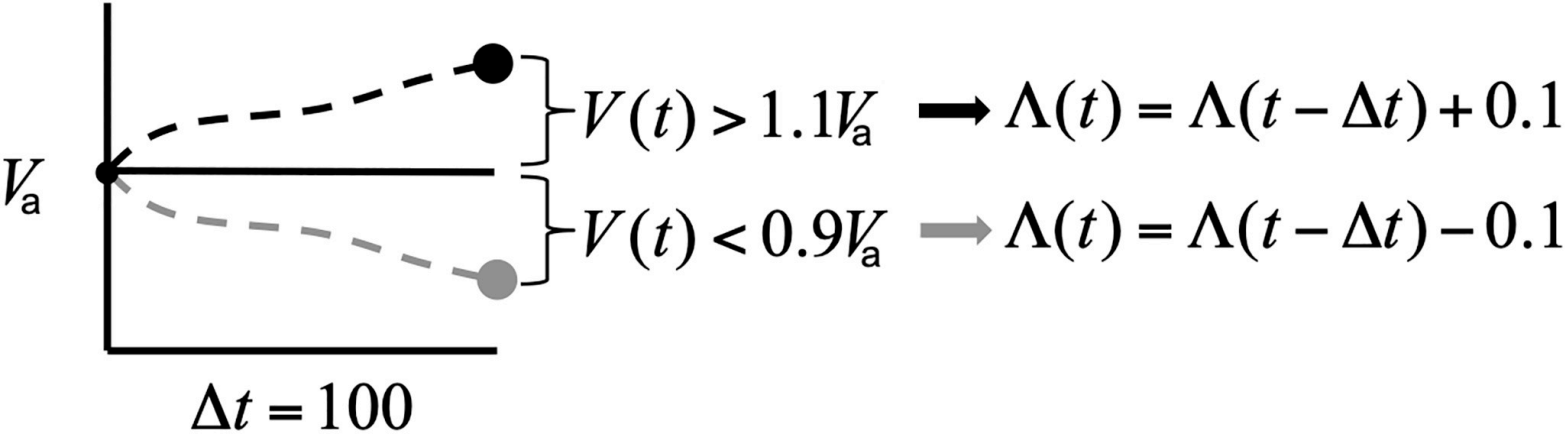
Dose adjustment schematic. Schematic description of the dose adjustment rules based on the measured total tumor volume shown here, attempting to maintain a total tumor burden at *V*_*a*_, though the same rules apply to *V*_*b*_.

Here we allow ourselves to change the abiraterone dose in the increments of 0.1 (i.e. Λ(*t*) ∈ {0.1, …, 1}), where the dose change may occur at the time of volume measurement. If the current *V*(*t*) increases above 110% of the tumor volume we are attempting to maintain (*V*_*a*_ or *V*_*b*_), the dose is increased by 0.1. If *V*(*t*) decreases below 90% of the tumor volume we are attempting to maintain, the dose is decreased by 0.1. If the tumor burden *V*(*t*) is within 0.9 and 1.1 of the target volume, the dose remains unchanged (Fig 5).

While the optimal control results suggest an initial dose Λ(*t*_0_) = 0, here we run additional simulations to compare this optimized result to the protocols suggested in [32] and [33] in in-vivo stabilization studies where the initial dose is Λ(*t*_0_) = 1 and the dose is titrated down. We compare all of the combinations of *V*_*a*_, *V*_*b*_, Λ(*t*_0_) = 0 and Λ(*t*_0_) = 1 to the clinical standard of care (maximum tolerated dose) where Λ(*t*) = 1 for all *t* ∈ [*t*_0_, *t*_*f*_] and the adaptive therapy protocol used in [8]. In this way, we model six clinically feasible protocols:

1. Maximum tolerated dose
2. Adaptive therapy cutting the initial volume by 50%.
3. Stabilization at initial tumor volume *V*_*a*_, with Λ(*t*_0_) = 1.
4. Stabilization at initial tumor volume *V*_*a*_ with Λ(*t*_0_) = 0.
5. Stabilization at maximum tolerated tumor volume *V*_*b*_ with Λ(*t*_0_) = 1.
6. Stabilization at maximum tolerated tumor volume *V*_*b*_ with Λ(*t*_0_) = 0.

**Fig 6:**
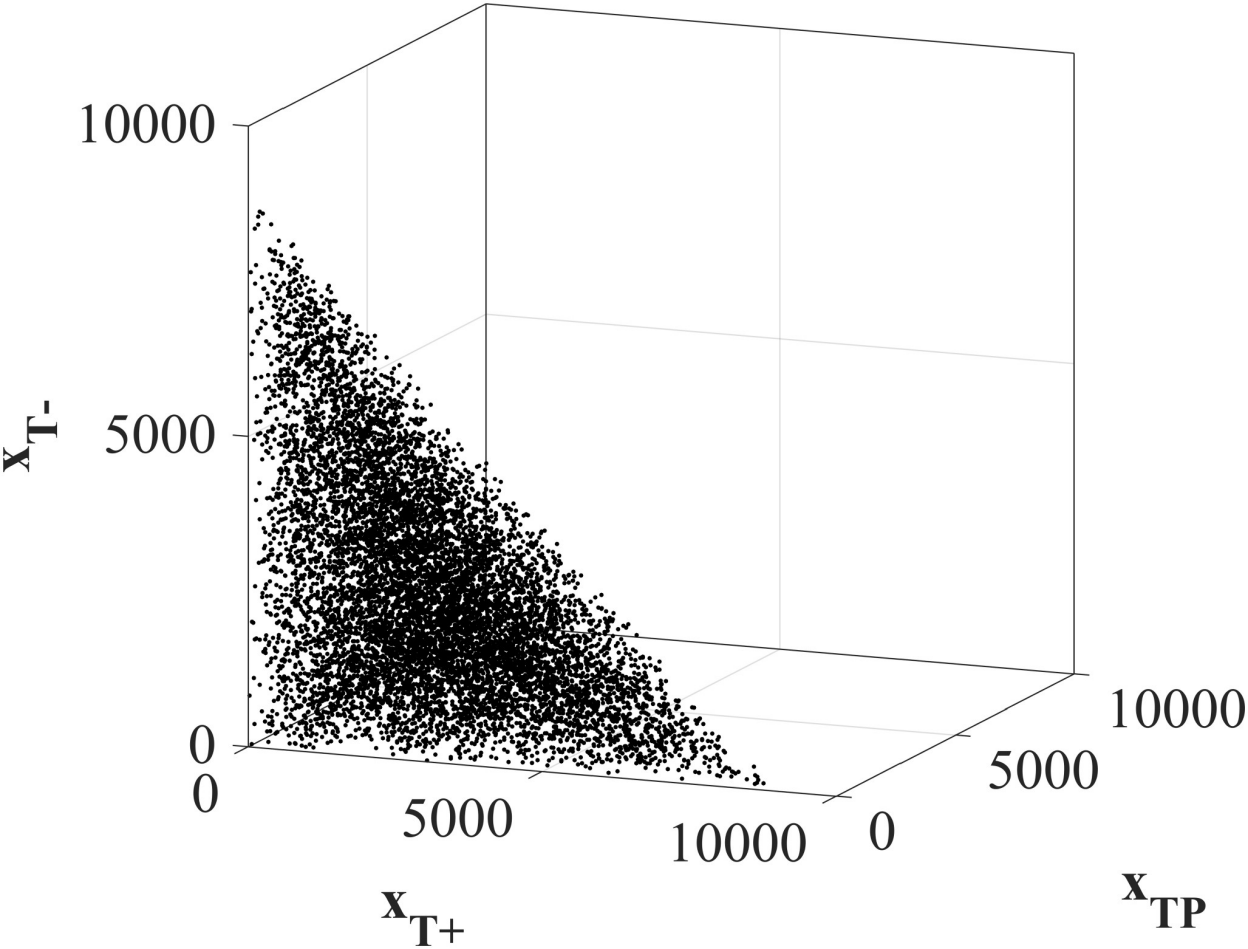
Initial tumor compositions for clinical feasible protocols. 10, 000 randomly selected initial tumor compositions used to analyze the clinically feasible protocols. All total tumor volumes satisfy the patient viability constraint $\sum_{i\in T}x_{i}\left(t_{0}\right)\leq 9000$.

Since clinically the initial tumor composition will be unknown, we test 10, 000 initial tumor compositions (*x*_*T*^+^_(*t*_0_), *x*_*T*^*P*^_(*t*_0_), *x*_*T*^−^_(*t*_0_)), as shown in Fig 6.

## 6 Outcomes of clinically feasible protocols

**Fig 7:**
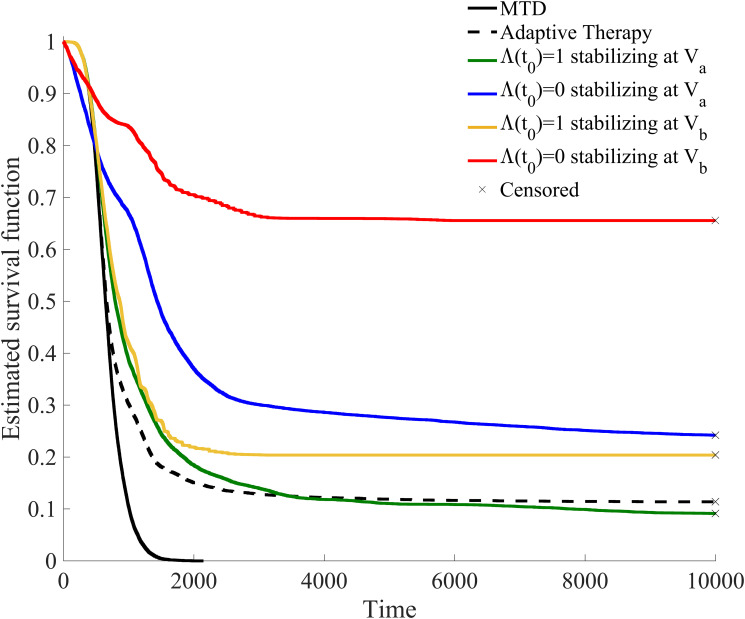
Kaplan-Meier survival analysis for clinically feasible treatment strategies. 10, 000 patients were given each of the six clinically feasible treatment strategies. In this way this shows the outcome of 60, 000 simulated patients. Patients that had not yet breached the patient viability constraint by the end of the simulation are labeled as censored.

In Fig 7, a Kaplan-Meier survival analysis is provided for the total of 60, 000 simulated patients under the six treatment strategies.

The percentage of these simulated patients that breached the patient viability constraint (5) and the mean and standard deviation of the time of this breach are summarized in Table 1. Each protocol is discussed in detail below, though three main takeaways are apparent:

1. MTD results in 100% of the patients violating the viability constraint at an average of 667.38 simulated time units or roughly corresponding to just over 21 months. This falls within the overall survival reported from patients with and without previous treatment with docetaxel (14.8 months and 53.3 months, respectively) [55, 56],
2. adaptive therapy can provide permanent control for only a small subset (11.39%) of initial tumor compositions, and
3. the most successful therapy in terms of patients surviving until the end of the simulation is titrating up from an initial dose of Λ(*t*_0_) = 0 and allowing for a large tumor volume. This results in 65.55% of the 10, 000 initial tumor compositions simulated to not breach the patient viability constraint.

**Table 1:** Survival statistics for clinically feasible treatment strategies.

Treatment	% Patients death	Mean (SD) time of death
MTD	100%	667.38(246.66)
Adaptive Therapy	88.61%	815.97(687.65)
*V*_*a*_, Λ(*t*_0_) = 1	90.78%	1112.62(1209.67)
*V*_*a*_, Λ(*t*_0_) = 0	75.80%	1412.53(1519.43)
*V*_*b*_, Λ(*t*_0_) = 1	89.61%	817.07(429.51)
*V*_*b*_, Λ(*t*_0_) = 0	34.45%	1108.09(918.98)

Percentage of simulated patients that breached the patient viability constraint before the end of simulation (*t*_*f*_ = 10000) and the average time of this breach.

### 6.1 Maximum tolerated dose dynamics

**Fig 8:**
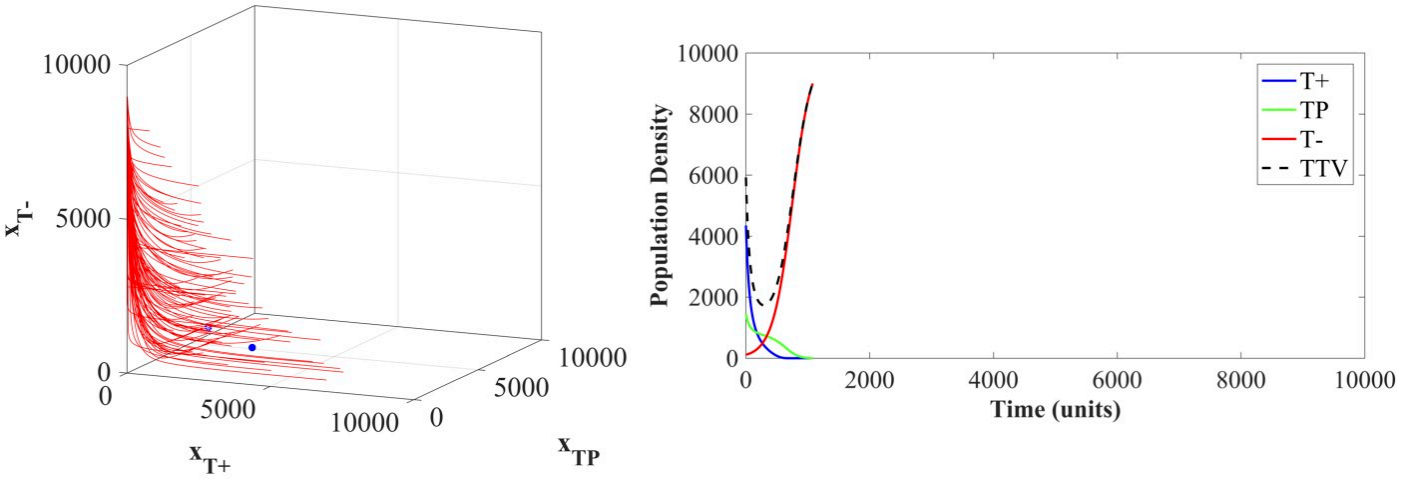
Maximum tolerable dose state dynamics. State trajectories for 100 initial tumor compositions (left panel), used for optimization of patients under the maximum tolerated dose protocol. All state trajectories end when the total tumor burden violates the patient viability constraint (5). The two blue dots show the location of the two equilibria (two- and three- species). The right panel shows the population densities of the three cell types in a representative case.

Using maximum tolerated dose (Λ(*t*) = 1 for all *t* ∈ [*t*_0_, *t*_*f*_]) eliminates the *T*^+^ and *T*^*P*^ cells and the tumor composition quickly becomes dominated by *T*^−^, as shown in Fig 8. All of the patients breach the viability constraint within a relatively short simulated time, with an average time of 667.38 time units.

### 6.2 Adaptive therapy dynamics

**Fig 9:**
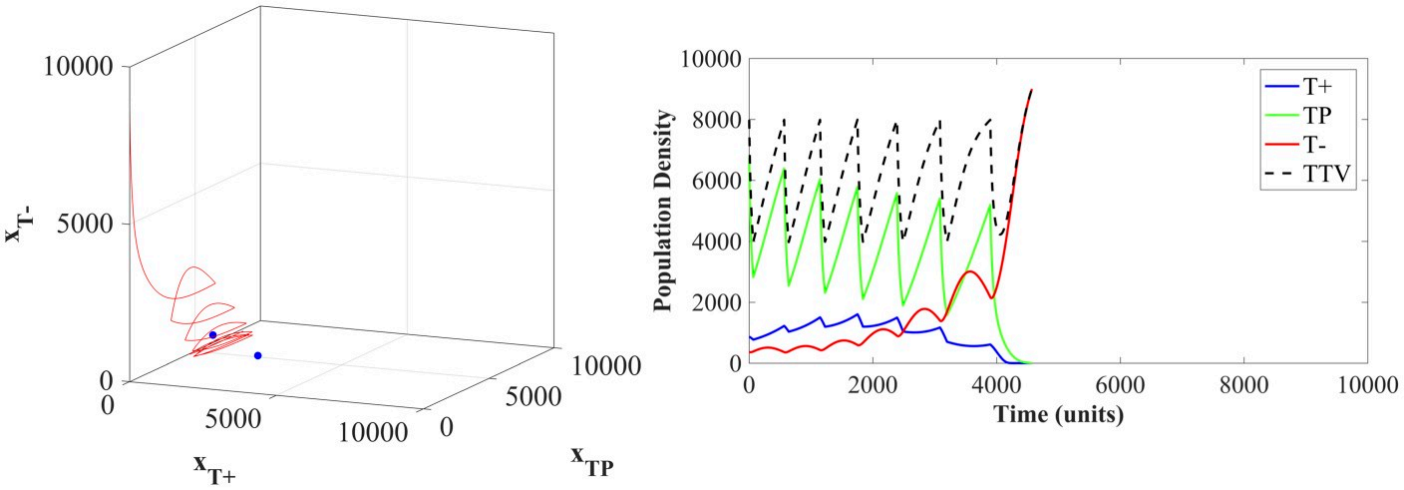
Adaptive therapy state dynamics. The state trajectory (left panel) and population densities (right panel) of an example patient under the adaptive therapy protocol. The two blue dots show the location of the two stable equilibria (two- and three- species).

Adaptive therapy protocols result in an average time to breaching the viability constraint of 815.97 simulated time points. This increase in survival beyond the MTD standard of care is due to adaptive therapy delaying the competitive release of the *T*^−^ population. Unfortunately, the treatment windows where Λ(*t*) = 1 ratchet the population towards a tumor composed of all *T*^−^ cells and cause the state trajectories to miss the stable equilibria. An example of the ultimate failure of the adaptive therapy is shown in Fig 9.

### 6.3 Dose titration dynamics

**Fig 10:**
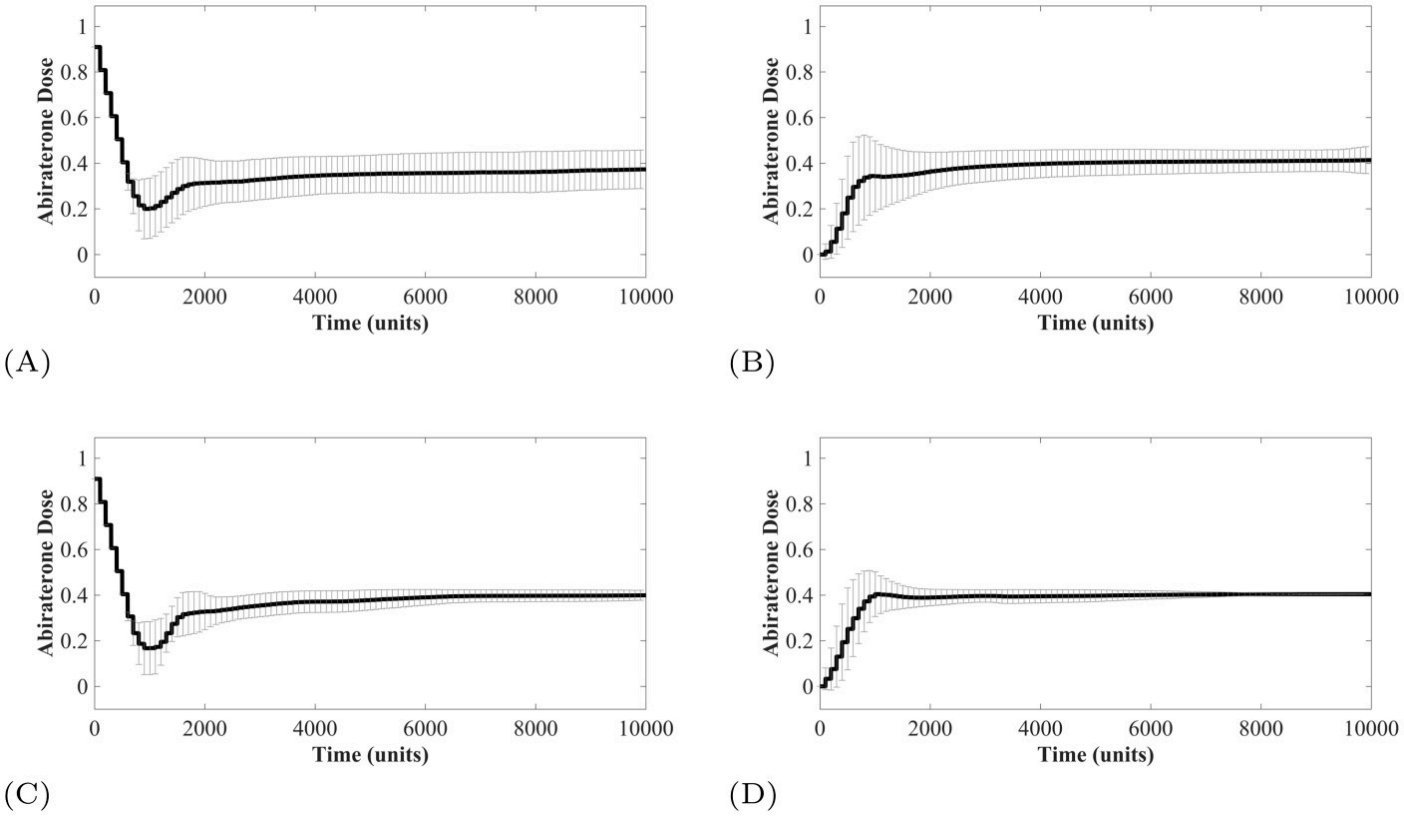
Titration protocols resulting in patient survival. Average titration protocols of patients that did not breach the patient viability constraint within the simulation time. The standard error of the mean (SEM) is on the order of 10^−3^ for all cases, therefore here the error bars show one standard deviation. (A) Λ(*t*_0_) = 1 stabilizing at *V*_*a*_. (B) Λ(*t*_0_) = 0 stabilizing at *V*_*a*_. (C) Λ(*t*_0_) = 1 stabilizing at *V*_*b*_. (D) Λ(*t*_0_) = 0 stabilizing at *V*_*b*_.

For the titration protocols, the mean successful treatment strategy of the surviving patients is shown in Fig 10. Of note, for the treatments with an initial dose Λ(*t*_0_) = 0 (panel B and D), the titration protocol developed in real time directly mimics the protocol identified by the optimal protocol found by the optimal control analysis shown in (Fig 3). These results show that a simple set of titration rules with no prior knowledge of the initial tumor composition nor the existence or location of an equilibrium can be used to stabilize a population at an equilibrium point. Interestingly, all of these surviving simulated patients under the titration protocols end the simulation at the two-species equilibrium point where Λ = 0.4 with *T*^+^ = 2068.97 and *T*^*P*^ = 5172.41. Since intermediate values of Λ are not available in the chosen dosage scheme, the entire region Λ ∈ [0.4828, 0.4877) where a three-species equilibrium is located, is unreachable. While the population dynamics may pass by a three-species equilibrium point, stabilizing there is unlikely, due to the discrete values of Λ available. More gradual changes in dose will however allow to reach the three-species equilibrium.

**Fig 11:**
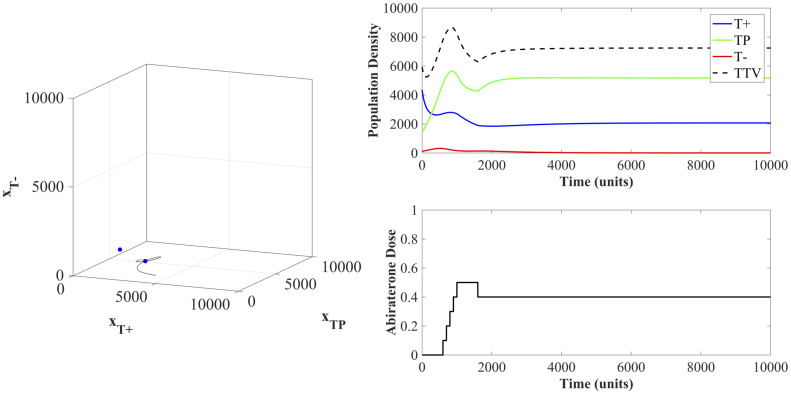
State dynamics of patient undergoing titration protocol. The dynamics here show an example patient under the initial dose of Λ(*t*_0_) = 0 and attempting to stabilize at a tumor volume equal to *V*_*b*_ = 7000. The state trajectory in the left panel shows the population arriving at the two-species equilibrium. The population densities and abiraterone dose are shown in the right panel.

A specific example of dose titration with an initial dose of Λ(*t*_0_) = 0 and attempting to stabilize at the previously defined maximum tolerated tumor volume of *V*_*b*_ = 7000 is shown in Fig 11. Interestingly, no drug was given for over 500 simulated time units. This is the time required for the tumor volume to exceed 7700 (110% of *V*_*b*_ = 7000) at which point the dose keeps increasing until the stabilizing dose of Λ = 0.4 in reached. The population dynamics show that while *T*^−^ cells are present in the initial tumor, allowing the *T*^+^ and *T*^*P*^ cells to remain and even increase in density prevents the competitive release of these *T*^−^ cells. In this example, the population of *T*^+^ and *T*^*P*^ cells can then be maintained at their equilibrium using a constant dose Λ = 0.4. An example of all six clinically feasible therapies on one simulated patient is available in S7 in S1 File.

## 7 Effect of initial tumor composition on treatment outcome

**Fig 12:**
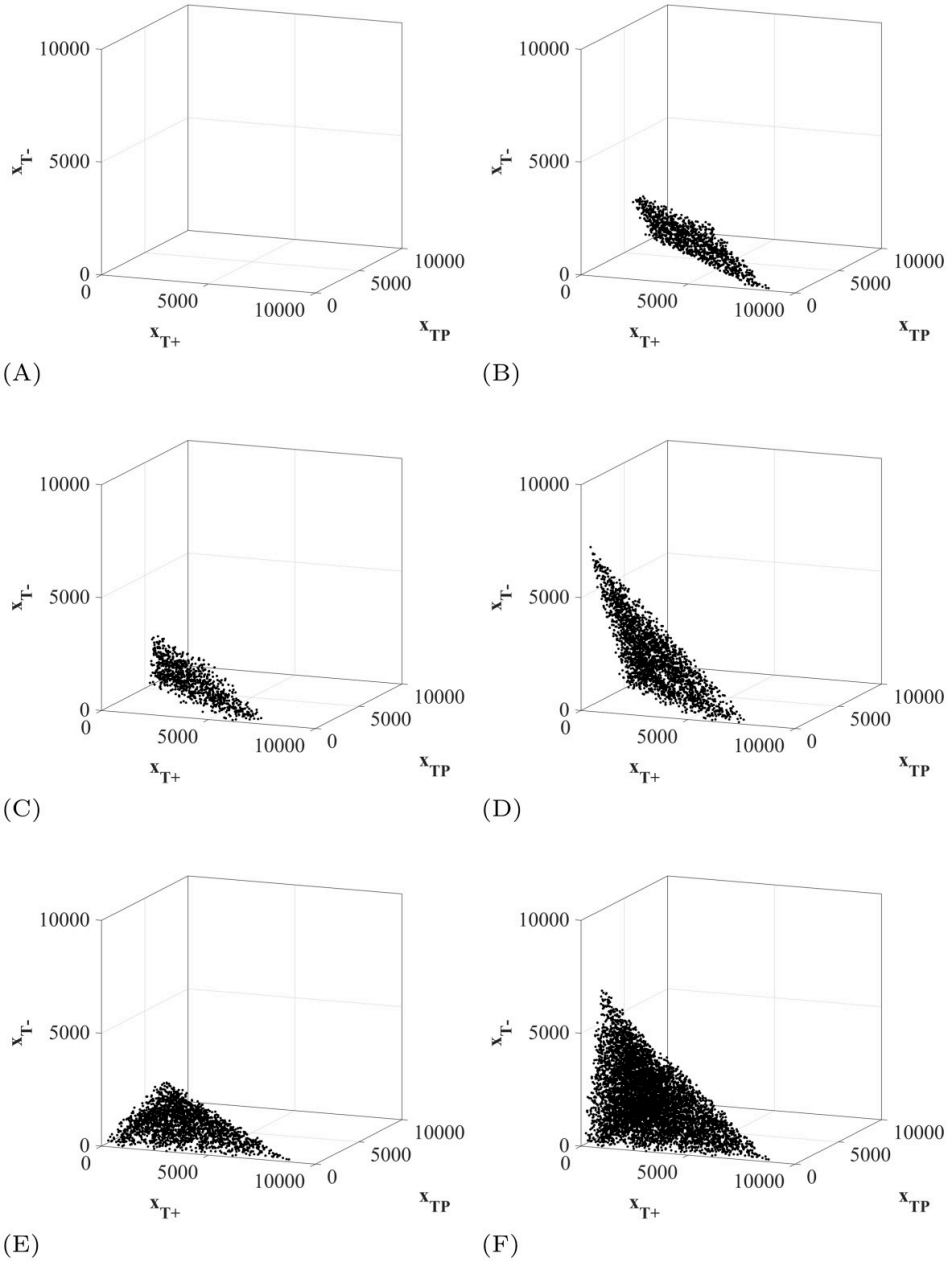
Initial tumor compositions of surviving patients. The initial tumor compositions of the patients that did not breach the patient viability constraint within the simulation time for each of the six clinically feasible protocols. Their two dimensional projections are available in S9 in S1 File. (A) Maximum tolerable dose. (B) Adaptive therapy. (C) Λ(*t*_0_) = 1 stabilizing at *V*_*a*_. (D) Λ(*t*_0_) = 0 stabilizing at *V*_*a*_. (E) Λ(*t*_0_) = 1 stabilizing at *V*_*b*_. (F) Λ(*t*_0_) = 0 stabilizing at *V*_*b*_.

The initial tumor composition has a large effect on the outcomes of the treatment protocols. In Fig 12, we show the initial tumor compositions that survive until the end of the simulation time for each of the protocols studied. Firstly, no patients survive to the end of simulation under MTD (Fig 12A). More interestingly, the patients that survive using adaptive therapy all begin within a small region of initial tumor compositions (Fig 12B). Using adaptive therapy, the 11.39% of patients who survive have very large initial tumor volumes (>7774) and relatively small *T*^−^ populations (<33.6% of the initial tumor volume). This combination is required as even short doses at Λ = 1 allow the *T*^−^ opportunity to grow, as seen in Fig 9.

The patients that survive using the titration protocol attempting stabilization at initial tumor volume *V*_*a*_ with Λ(*t*_0_) = 1 also have large initial tumor volumes (>5400) and small populations of *T*^−^ (Fig 12C). On the other hand, the titration protocol attempting stabilization at initial tumor volume where Λ(*t*_0_) = 0 (Fig 12D) still requires a high tumor volume to survive, but can tolerate much higher initial densities of *T*^−^ cells. For both cases, the minimum tumor volume that could be stabilized was 5195. Again, an initial dose of Λ(*t*_0_) = 0 allows patients with higher initial frequencies of *T*^−^ cells to survive as any doses at Λ = 1 allow the *T*^−^ opportunity to grow, as seen in Fig 9.

Furthermore, attempting stabilization at a maximum tolerated tumor volume allows patients with small initial tumor volumes to survive (Fig 12E and 12F). It is important to note that allowing these patients’ tumors to grow will not decrease their quality of life. So while it is psychologically difficult to intentionally let a small initial tumor burden grow, it could potentially provide clinical benefits. With an initial dose of Λ(*t*_0_) = 1, the initial *T*^−^ population must still be small in order to avoid competitive release of the *T*^−^ population at early treatment stage, regardless of the tumor volume. By setting Λ(*t*_0_) = 0, even patients with small initial tumor volumes and high initial frequencies of *T*^−^ cells can survive to the end of the simulation.

### 7.1 Tumor composition at time of death for clinically feasible protocols

**Fig 13:**
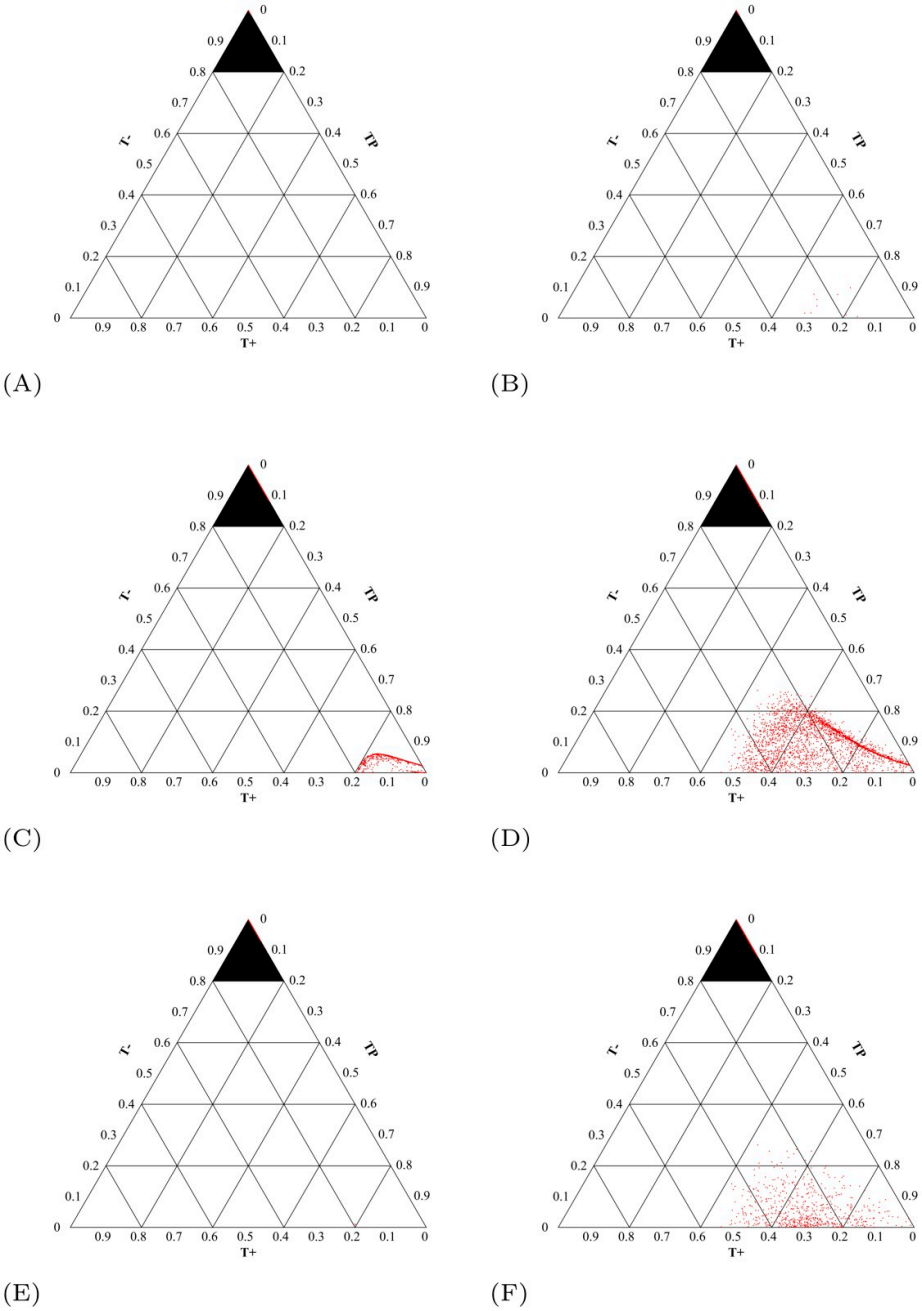
Tumor composition at time of viability constraint breach. Ternary plots where each red dot indicates the tumor composition of *T*^+^, *T*^*P*^, and *T*^−^ cells at the time a patient reached the viability constraint. (Figures made using [57].) The top highlighted triangle in each figure encompasses the tumor compositions with >80% *T*^−^ cells. Patients with tumor compositions located in this upper triangle suffered from competitive release of the *T*^−^ cells. Outside of this upper triangle, treatable cells were still present at the time of viability constraint breach. (A) 100% of patients are located in the top triangle: *n* = 10000. (B) Adaptive Therapy. 99.90% of patients are located in the top triangle: *n* = 8861. (C) Λ(*t*_0_) = 1 stabilizing at *V*_*a*_. 87.13% of patients are located in the top triangle: *n* = 9088. (D) Λ(*t*_0_) = 0 stabilizing at *V*_*a*_. 60.30% of patients are located in the top triangle: *n* = 7580. (E) Λ(*t*_0_) = 1 stabilizing at *V*_*b*_. 99.97% of patients are located in the top triangle: *n* = 7961. (F) Λ(*t*_0_) = 0 stabilizing at *V*_*b*_. 81.86% of patients are located in the top triangle: *n* = 3445.

It is important also to understand the composition of the tumor that caused the patient to cross the viability constraint to understand why the treatment failed. In Fig 13, the tumor composition at the time of crossing the patient viability constraint is presented for each treatment. For the treatment protocols giving high doses—MTD, adaptive therapy, and Λ(*t*_0_) = 1 stabilizing at *V*_*b*_—the vast majority of the patients died of tumors comprised completely of *T*^−^ cells. This makes sense as the high doses of abiraterone given throughout or early in treatment will eliminate the *T*^+^ and *T*^*P*^ cells, causing the competitive release of *T*^−^ and eventual treatment failure.

For the patients undergoing the treatment protocol where Λ(*t*_0_) = 1 stabilizing at *V*_*a*_, the 12.87% that had a tumor comprised mostly of *T*^+^ and *T*^*P*^ at the time of treatment failure had large initial tumor volumes, and therefore large values of *V*_*a*_ that were >8000. In this way, while the initial dose of abiraterone was Λ = 1, the protocol titrates down very quickly in order to maintain the desired tumor volume. Unfortunately, this generally results in an under treatment of the tumor and the patient crossing the viability constraint with *T*^+^ and *T*^*P*^ cells remaining.

Additionally, the tumor compositions at the time of treatment failure of the patients receiving initial low doses of abiraterone—Λ(*t*_0_) = 0 stabilizing at *V*_*a*_ and Λ(*t*_0_) = 0 stabilizing at *V*_*b*_—show that 39.7% and 18.14% of the patients who crossed the viability constraint, respectively, had high percentages of *T*^+^ and *T*^*P*^ cells remaining. Since these cells are treatable by abiraterone, these patients were indeed under treated by the treatment protocol.

These results show that there is an important balance between giving too much abiraterone causing competitive release of resistant cells, and not giving enough abiraterone causing treatment failure even with treatable cells remaining in the tumor.

## 8 Discussion

Here we developed and analyzed an ‘evolutionary stable therapy’ in mCRPC that can maintain a stable polymorphic tumor heterogeneity of sensitive and resistant cells to abiraterone, in order to prolong treatment efficacy and progression free survival. Surprisingly, in the majority of simulated patients, the optimal control analysis suggests a simple increasing dose titration protocol to achieve stabilization. While a single formulation of the competition matrix is presented here, three additional clinically relevant matrices were investigated resulting in the analysis of a total of seven possible stable points. The optimal control analysis consistently suggests a simple increasing dose titration protocol to achieve stabilization (see S5 in S1 File). Furthermore, the outcomes of the simulated clinically feasible protocols show that increasing dose titration protocols invariably increased progression free survival in the majority of patients (see S8 in S1 File). This suggests that if the properties of the underlying biology allow stabilization, regardless of the actual composition of the stable polymorphic tumor heterogeneity, an increasing dose titration protocol may, in general, provide an appropriate dosing strategy to achieve stabilization.

Dose titration is a very common protocol used with drugs like insulin, anti-depressants, and opioids, to find the optimal dose of a medication while minimizing the adverse side effects, physical or financial [58–60]. Most notably in oncology, a ‘ramp-up’ protocol for Venetoclax is used in patients with chronic lymphocytic leukemia in order to limit tumor lysis syndrome (physical toxicity) [61]. In patients with hepatocellular carcinoma a dose titration of sorafenib is used to significantly lower overall cost (financial toxicity) while maintaining equivalent survival [62]. Interestingly, some initial studies of dose titration protocols show benefit beyond toxicity management. For example, titration of axitinib resulted in a greater proportion of patients with metastatic renal cell carcinoma achieving an objective response and, incredibly, titration of regorafenib in patients with metastatic colorectal cancer actually increased median overall survival from 5.9 months (initiating treatment at standard dose) to 9.0 months [63, 64].

Interestingly, in the case of abiraterone titration, our analysis also showed that larger tumor volumes may counter intuitively be more likely to be stabilized if sensitive cells dominate the tumor composition at time of initial treatment, suggesting a delay of initial treatment could prove beneficial. This reiterates previous analysis of this model comparing intermittent abiraterone to optimized treatments concluding that delaying treatment for as long as possible, while increasing tumor volume, maintained a larger sensitive population and resulted in prolonged tumor control [50]. This result is also seen in other disciplines such as agricultural pest management, equine parasite management, and bacterial infection management where large sensitive populations can contain resistant populations [12, 65, 66].

If stabilization of the tumor is possible, the use of titration to reach an equilibrium of metastatic disease could have many benefits such as prolonging progression free survival and administering lower doses of drug leading to less cumulative drug over the length of the treatment. While the goal of treating any cancer is to allow the patient to live a normal life span, a titration protocol will also generally increase patient quality of life by limiting the toxicity related side effects of cancer drugs. Furthermore, delaying the absolute growth of disease within a patient could allow other physiological processes, such as vascular normalization and the immune system, that have little effect on large rapidly growing tumors to play a greater role in patient outcomes [33]. It is also possible that curative strategies using application of additional drugs or immune therapies could be more effective in a stabilized tumor environment [67–74].

With any novel treatment protocol, there are potential drawbacks. Analysis here showed that it is possible to either undertreat or overtreat patients using a titration protocol. If a patient is already experiencing quality of life issues because of high tumor burden, beginning at low doses in a titration protocol is not wise. For these patients, much like in the two mouse models where the initial exponential growth required immediate intervention, it may be necessary to use a more aggressive approach, like the decreasing dose titration protocol used in the in-vitro mouse models or adaptive therapy. Overtreatment, on the other hand, could be mitigated by more frequent PSA measurements in order to react more quickly to changes in tumor response and limit the competitive release of resistant cells during therapy [75]. In reality, it is likely that PSA alone will be insufficient to guide detailed evolutionary protocols such as the one discussed here [76]. Additional information particularly related to the underlying tumor composition such as DHT-PET imaging or AR-V7 expression from circulating tumor cells could greatly improve evolutionary management in mCRPC [77–80]. An ideal implementation would be to consider using drug pumps like those used in insulin management for continuous measurement and administration of cancer therapeutics [81].

The outcomes of this study are heavily dependent on the underlying mathematical model used and its parameterization [82]. As with any evolutionary game, the competition coefficients are of particular interest [83]. Once the clinical trial that was designed using the model studied here is completed, parameter optimization of the competition matrix using patient data from both the standard of care maximum tolerable dose cohort as well as the adaptive therapy cohort can be performed. While studies are now attempting to measure these intratumoral competitive properties in-vitro [34], more detailed experimental work will be required before understanding and attempting stabilization in-vivo [28].

Furthermore, this particular model studied here does not encompass the full complexity of metastatic disease within a patient. For example, phenotypic switching, which can be implicitly accounted for in population models like the one used here, is not modeled explicitly and could alter the dynamics of treatment outcomes [84–87]. Furthermore, this model assumes no new mutations resulting in novel phenotypes occur during treatment, which is likely not true. If a new resistant phenotype emerges, this will ultimately change the dynamics of the game and the stability properties [88]. It will require further in-vivo analysis to show that either new mutations cannot invade the tumor population or that these mutations occur late enough that the patient succumbs to another cause of death before treatment failure. As in other ecological systems, it is still unknown whether stability of both the ecological and evolutionary dynamics is feasible and robust, and will remain unknown in metastatic disease until further experiments along this line are performed [89, 90].

The effects of the spatial structure within a heterogeneous tumor population is not explicitly studied in this model, though have been shown to affect stabilization properties [91–94]. Interestingly, [49] added a spatial structure to the model used here and showed that the interaction neighborhood size and the effects of carrying capacity affect the stability properties. In this way, it would be of great interest to identify ‘evolutionary stable therapies’ in other models of prostate cancer that model treatments as death rates or reductions in growth rates, address the importance of cell turnover, and include spatial structure [95–103].

The clinical development of an evolutionary stable therapy described here could provide immediate and substantial benefits to both patient quantity and quality of life. A better understanding of the properties of disease that make evolutionary therapies superior to current standard of care and the psychological shift required are of great interest [67, 104]. While it remains uncertain if metastatic disease in humans has the properties that allow it to be truly stabilized, the benefits of a dose titration protocol warrant additional pre-clinical and clinical investigations.

## Supporting information

S1 File (ZIP)

## References

[1] Jemal A, Ward EM, Johnson CJ, Cronin KA, Ma J, Ryerson AB, et al Annual Report to the Nation on the Status of Cancer, 1975–2014, Featuring Survival. JNCI: Journal of the National Cancer Institute. 2017;109(9). 10.1093/jnci/djx030 28376154 PMC5409140

[2] Nakazawa M, Paller C, Kyprianou N. Mechanisms of Therapeutic Resistance in Prostate Cancer. Current Oncology Reports. 2017;19:13 10.1007/s11912-017-0568-7 28229393 PMC5812366

[3] Holohan C, Schaeybroeck S, Longley DB, Johnston PG. Cancer drug resistance: an evolving paradigm. Nature Reviews Cancer. 2013;13:714–726. 10.1038/nrc3599 24060863

[4] Attolini CSO, Michor F. Evolutionary Theory of Cancer. Annals of the New York Academy of Sciences. 2009;1168(1):23–51. 10.1111/j.1749-6632.2009.04880.x 19566702

[5] Greaves M, Maley C. Clonal evolution in cancer. Nature. 2012;481 10.1038/nature10762 PMC3367003 22258609

[6] Merlo LM, Pepper JW, Reid BJ, Maley CC. Cancer as an evolutionary and ecological process. Nature Reviews Cancer. 2006;6(12):924–935. 10.1038/nrc2013 17109012

[7] Stankova K. Resistance games. Nature Ecology & Evolution. 2019;3:336–337. 10.1038/s41559-018-0785-y 30778182

[8] Zhang J, Cunningham JJ, Brown JS, Gatenby RA. Integrating evolutionary dynamics into treatment of metastatic castrate-resistant prostate cancer. Nature Communications. 2017;8(1):1816 10.1038/s41467-017-01968-5 PMC5703947 29180633

[9] Smalley I, Kim E, Li J, Spence P, Wyatt CJ, Eroglu Z, et al Leveraging transcriptional dynamics to improve BRAF inhibitor responses in melanoma. EBioMedicine. 2019;48:178–190. 10.1016/j.ebiom.2019.09.023 31594749 PMC6838387

[10] Kam Y, Das T, Minton S, Gatenby RA. Evolutionary strategy for systemic therapy of metastatic breast cancer: Balancing response with suppression of resistance. Womens Health. 2014;10. 10.2217/whe.14.23 PMC4258899 25259902

[11] Zhang J, Fishman MN, Brown J, Gatenby RA. Integrating evolutionary dynamics into treatment of metastatic castrate-resistant prostate cancer (mCRPC): Updated analysis of the adaptive abiraterone (abi) study (NCT02415621). Journal of Clinical Oncology. 2019;37(15_suppl):5041–5041. 10.1200/JCO.2019.37.15_suppl.5041

[12] Hansen E, Karslake J, Woods RJ, Read AF, Wood KB. Antibiotics can be used to contain drug-resistant bacteria by maintaining sufficiently large sensitive populations. bioRxiv. 2019;. 10.1101/638924 PMC7266357 32413038

[13] Tabashnik BE, Brévault T, Carrière Y. Insect resistance to Bt crops: lessons from the first billion acres. Nature biotechnology. 2013;31(6):510 10.1038/nbt.2597 23752438

[14] Pouchol C, Clairambault J, Lorz A, Trélat E. Asymptotic analysis and optimal control of an integro-differential system modelling healthy and cancer cells exposed to chemotherapy. Journal de Mathématiques Pures et Appliquées. 2018;116:268–308.

[15] Smith JM, Price GR. The logic of animal conflict. Nature. 1973;246(5427):15–18. 10.1038/246015a0

[16] Smith JM. Evolution and the Theory of Games. Cambridge university press; 1982.

[17] Hofbauer J, Sigmund K. Evolutionary Games and Population Dynamics. Cambridge University Press; 1998.

[18] Nash JF, et al Equilibrium points in n-person games. Proceedings of the national academy of sciences. 1950;36(1):48–49. 10.1073/pnas.36.1.48 16588946 PMC1063129

[19] von Neumann J, Morgenstern O. Theory of Games and Economic Behavior. Princeton University Press; 1944.

[20] Tomlinson IP. Game-theory models of interactions between tumour cells. European Journal of Cancer. 1997;33:1495–1500. 10.1016/S0959-8049(97)00170-6 9337695

[21] Gatenby RA, Vincent TL. Application of quantitative models from population biology and evolutionary game theory to tumor therapeutic strategies. Molecular cancer therapeutics. 2003;2(9):919–927. 14555711

[22] Vincent TL, Gatenby RA. Modeling cancer as an evolutionary game. International Game Theory Review. 2005;7(03):331–346. 10.1142/S0219198905000557

[23] Dingli D, Chalub F, Santos F, Van Segbroeck S, Pacheco J. Cancer phenotype as the outcome of an evolutionary game between normal and malignant cells. British Journal of Cancer. 2009;101(7):1130–1136. 10.1038/sj.bjc.6605288 19724279 PMC2768082

[24] McEvoy J. Evolutionary game theory: lessons and limitations, a cancer perspective. British journal of cancer. 2009;101(12):2060–2061. 10.1038/sj.bjc.6605444 19920827 PMC2795450

[25] Gallaher JA, Enriquez-Navas PM, Luddy KA, Gatenby RA, Anderson ARA. Spatial Heterogeneity and Evolutionary Dynamics Modulate Time to Recurrence in Continuous and Adaptive Cancer Therapies. Cancer Research. 2018;78(8):2127–2139. 10.1158/0008-5472.CAN-17-2649 29382708 PMC5899666

[26] Martin RB, Fisher ME, Minchin RF, Teo KL. Optimal control of tumor size used to maximize survival time when cells are resistant to chemotherapy. Mathematical Biosciences. 1992;110(2):201–219. 10.1016/0025-5564(92)90038-X 1498450

[27] Zeeman EC. Population dynamics from game theory In: Global theory of dynamical systems. Springer; 1980 p. 471–497.

[28] Kaznatcheev A, Peacock J, Basanta D, Marusyk A, Scott JG. Fibroblasts and alectinib switch the evolutionary games played by non-small cell lung cancer. Nature ecology & evolution. 2019;3(3):450–456. 10.1038/s41559-018-0768-z 30778184 PMC6467526

[29] West J, Ma Y, Newton PK. Capitalizing on competition: An evolutionary model of competitive release in metastatic castration resistant prostate cancer treatment. Journal of Theoretical Biology. 2018;455:249–260. 10.1016/j.jtbi.2018.07.028 30048718 PMC7519622

[30] Bach LA, Bentzen S, Alsner J, Christiansen FB. An evolutionary-game model of tumour–cell interactions: possible relevance to gene therapy. European Journal of Cancer. 2001;37(16):2116–2120. 10.1016/S0959-8049(01)00246-5 11597393

[31] Cross WC, Graham TA, Wright NA. New paradigms in clonal evolution: punctuated equilibrium in cancer. The Journal of pathology. 2016;240(2):126–136. 10.1002/path.4757 27282810

[32] Gatenby RA, Silva AS, Gillies RJ, Frieden BR. Adaptive therapy. Cancer Research. 2009;69(11):4894–4903. 10.1158/0008-5472.CAN-08-3658 19487300 PMC3728826

[33] Enriquez-Navas PM, Kam Y, Das T, Hassan S, Silva A, Foroutan P, et al Exploiting evolutionary principles to prolong tumor control in preclinical models of breast cancer. Science Translational Medicine. 2016;8(327):327ra24 10.1126/scitranslmed.aad7842 26912903 PMC4962860

[34] Freischel AR, Damaghi M, Cunningham JJ, Ibrahim-Hashim A, Gillies RJ, Gatenby RA, et al Frequency-dependent interactions determine outcome of competition between two breast cancer cell lines. bioRxiv. 2020;. 10.1038/s41598-021-84406-3 PMC7921689 33649456

[35] Archetti M, Ferraro DA, Christofori G. Heterogeneity for IGF-II production maintained by public goods dynamics in neuroendocrine pancreatic cancer. Proceedings of the National Academy of Sciences. 2015;112(6):1833–1838. 10.1073/pnas.1414653112 PMC4330744 25624490

[36] Dingli D, Offord C, Myers R, Peng KW, Carr T, Josic K, et al Dynamics of multiple myeloma tumor therapy with a recombinant measles virus. Cancer gene therapy. 2009;16(12):873 10.1038/cgt.2009.40 19498461 PMC2821809

[37] Archetti M. Evolutionary game theory of growth factor production: implications for tumour heterogeneity and resistance to therapies. British journal of cancer. 2013;109(4):1056–1062. 10.1038/bjc.2013.336 23922110 PMC3749558

[38] Gluzman M, Scott JG, Vladimirsky A. Optimizing adaptive cancer therapy: dynamic programming and evolutionary game theory. Proceedings of the Royal Society B. 2020;287(1925):20192454 10.1098/rspb.2019.2454 32315588 PMC7211445

[39] West J, You L, Brown J, Newton PK, Anderson ARA. Towards multi-drug adaptive therapy. bioRxiv. 2018;. 10.1101/476507

[40] Gerlee P, Altrock PM. Extinction rates in tumour public goods games. Journal of The Royal Society Interface. 2017;14(134):20170342 10.1098/rsif.2017.0342 PMC5636271 28954847

[41] Swan GW. Optimal control in some cancer chemotherapy problems. International Journal of Systems Science. 1980;11:223–. 10.1080/00207728008967009

[42] Swan GW. Cancer chemotherapy: Optimal control using the Verhulst-Pearl equation. Bulletin of Mathematical Biology. 1986;48:381–. 10.1007/BF02459688 3828564

[43] Swan GW. General applications of optimal control theory in cancer chemotherapy. IMA J Math Appl Med Biol. 1988;5(5):303–. 10.1093/imammb/5.4.303 3241099

[44] Swan GW. Role of optimal control therapy in cancer chemotherapy. Mathematical Biosciences. 1990;101(2):237–. 10.1016/0025-5564(90)90021-P 2134485

[45] Swan GW, Vincent TL. Optimal control analysis in the chemotherapy of IgG multiple myeloma. Bulletin of Mathematical Biology. 1977;39(3):317–337. 10.1016/S0092-8240(77)80070-0 857983

[46] Orlando PA, Gatenby RA, Brown JS. Cancer treatment as a game: integrating evolutionary game theory into the optimal control of chemotherapy. Physical Biology. 2012;9(6):065007 10.1088/1478-3975/9/6/065007 23197192 PMC3653600

[47] Wang S, Schattler H. Optimal control of a mathematical model for cancer chemotherapy under tumor heterogeneity. Mathematical Biosciences and Engineering. 2016;13(6):1223–1240. 10.3934/mbe.2016040 27775377

[48] Carrere C. Optimization of an in vitro chemotherapy to avoid resistant tumours. Journal of Theoretical Biology. 2017;413:24–33. 10.1016/j.jtbi.2016.11.009 27864095

[49] You L, Brown JS, Thuijsman F, Cunningham JS, Gatenby RA, Zhang J, et al Spatial vs. non-spatial eco-evolutionary dynamics in a tumor growth model. Journal of Theoretical Biology. 2017;435:78–97. 10.1016/j.jtbi.2017.08.022 28870617

[50] Cunningham JJ, Brown JS, Gatenby RA, Staňková K. Optimal control to develop therapeutic strategies for metastatic castrate resistant prostate cancer. Journal of Theoretical Biology. 2018;459:67–78. 10.1016/j.jtbi.2018.09.022 30243754

[51] Bacevic K, Noble R, Soffar A, Wael Ammar O, Boszonyik B, Prieto S, et al Spatial competition constrains resistance to targeted cancer therapy. Nature Communications. 2017;8 10.1038/s41467-017-01516-1 29222471 PMC5722825

[52] Grolmusz VK, Chen J, Emond R, Cosgrove PA, Pflieger L, Nath A, et al Exploiting collateral sensitivity controls growth of mixed culture of sensitive and resistant cells and decreases selection for resistant cells in a cell line model. Cancer Cell International. 2020;20(1):1475–2867. 10.1186/s12935-020-01337-1 PMC7301982 32565737

[53] McAsey M, Mou L, Han W. Convergence of the forward-backward sweep method in optimal control. Computational Optimization and Applications. 2012;53:207–226. 10.1007/s10589-011-9454-7

[54] Roden DM. Principles of clinical pharmacology. Kasper DL, Braunwald E, Fauci AS, Hauser SL, Longo. 1995;.

[55] de Bono JS, Logothetis CJ, Molina A, Fizazi K, North S, Chu L, et al Abiraterone and Increased Survival in Metastatic Prostate Cancer. New England Journal of Medicine. 2011;364(21):1995–2005. 10.1056/NEJMoa1014618 21612468 PMC3471149

[56] Fizazi K, Tran N, Fein L, Matsubara N, Rodriguez-Antolin A, Alekseev BY, et al Abiraterone acetate plus prednisone in patients with newly diagnosed high-risk metastatic castration-sensitive prostate cancer (LATITUDE): final overall survival analysis of a randomised, double-blind, phase 3 trial. The Lancet Oncology. 2019;20(5):686–700. 10.1016/S1470-2045(19)30082-8 30987939

[57] Sandrock C. Plot ternary diagrams in Matlab; 2020 Available from: https://github.com/alchemyst/ternplot.

[58] Wilson M, Weinreb J, Hoo GWS. Intensive Insulin Therapy in Critical Care. Diabetes Care. 2007;30(4):1005–1011. 10.2337/dc06-1964 17213376

[59] Huss M, Duhan P, Gandhi P, Chen CW, Spannhuth C, Kumar V. Methylphenidate dose optimization for ADHD treatment: review of safety, efficacy, and clinical necessity. Neuropsychiatric disease and treatment. 2017;13(1):1741–1751. 10.2147/NDT.S130444 28740389 PMC5505611

[60] Mercadante S. Opioid titration in cancer pain: A critical review. European Journal of Pain. 2007;11(8):823–830. 10.1016/j.ejpain.2007.01.003 17331764

[61] Roberts AW, Davids MS, Pagel JM, Kahl BS, Puvvada SD, Gerecitano JF, et al Targeting BCL2 with venetoclax in relapsed chronic lymphocytic leukemia. New England Journal of Medicine. 2016;374(4):311–322. 10.1056/NEJMoa1513257 26639348 PMC7107002

[62] Kaplan DE, Yu S, Taddei TH, Reiss KA, Mehta R, D’Addeo K, et al Up-titration of sorafenib for hepatocellular carcinoma: Impact on duration of exposure and cost.; 2017.

[63] Rini BI, Melichar B, Ueda T, Grünwald V, Fishman MN, Arranz JA, et al Axitinib with or without dose titration for first-line metastatic renal-cell carcinoma: a randomised double-blind phase 2 trial. The lancet oncology. 2013;14(12):1233–1242. 10.1016/S1470-2045(13)70464-9 24140184 PMC4120767

[64] Bekaii-Saab TS, Ou FS, Anderson DM, Ahn DH, Boland PM, Ciombor KK, et al Regorafenib dose optimization study (ReDOS): randomized phase II trial to evaluate dosing strategies for regorafenib in refractory metastatic colorectal cancer (mCRC)(a) over-cap (sic). An ACCRU network study. J Clin Oncol. 2018;36(4):0–3932.

[65] Barzman M, Bárberi P, Birch ANE, Boonekamp P, Saaydeh SD, Graf B, et al Eight principles of integrated pest management. Agron Sustain Dev. 2015;35:1199–1215. 10.1007/s13593-015-0327-9

[66] Nielsen MK. Sustainable equine parasite control: Perspectives and research needs. Veterinary Parasitology. 2012;185(1):32–44. 10.1016/j.vetpar.2011.10.012 22055611

[67] Hansen E, Read AF. Cancer therapy: attempt cure or manage drug resistance? Evolutionary Applications. 2020;. 10.1111/eva.12994 32821276 PMC7428817

[68] Yoon N, Vander Velde R, Marusyk A, Scott JG. Optimal therapy scheduling based on a pair of collaterally sensitive drugs. Bulletin of mathematical biology. 2018;80(7):1776–1809. 10.1007/s11538-018-0434-2 29736596

[69] Gatenby RA, Artzy-Randrup Y, Epstein T, Reed DR, Brown JS. Eradicating metastatic cancer and the eco-evolutionary dynamics of Anthropocene extinctions. Cancer Research. 2019;. 31772037 10.1158/0008-5472.CAN-19-1941 PMC7771333

[70] Gatenby RA, Zhang J, Brown JS. First Strike–Second Strike Strategies in Metastatic Cancer: Lessons from the Evolutionary Dynamics of Extinction. Cancer Research. 2019;. 10.1158/0008-5472.CAN-19-0807 31221821 PMC6606376

[71] Leung C, Weitz J. Modeling the synergistic elimination of bacteria by phage and the innate immune system. Journal of Theoretical Biology. 2017;429:241–252. 10.1016/j.jtbi.2017.06.037 28668337

[72] Thomas F, Donnadieu E, Charriere GM, Jacqueline C, Tasiemski A, Pujol P, et al Is adaptive therapy natural? PLOS Biology. 2018;16(10):1–12. 10.1371/journal.pbio.2007066 PMC6168119 30278037

[73] Bayer P, Brown JS, Staňková K. A two-phenotype model of immune evasion by cancer cells. Journal of Theoretical Biology. 2018;455:191–204. 10.1016/j.jtbi.2018.07.014 30031001

[74] Dhawan A, Nichol D, Kinose F, Abazeed ME, Marusyk A, Haura EB, et al Collateral sensitivity networks reveal evolutionary instability and novel treatment strategies in ALK mutated non-small cell lung cancer. Scientific Reports. 2017;7(1):1–9. 10.1038/s41598-017-00791-8 28450729 PMC5430816

[75] Fischer A, Vázquez-García I, Mustonen V. The value of monitoring to control evolving populations. Proceedings of the National Academy of Sciences. 2015;112(4):1007–1012. 10.1073/pnas.1409403112 PMC4313848 25587136

[76] Verbel DA, Heller G, Kelly WK, Scher HI. Quantifying the amount of variation in survival explained by prostate-specific antigen. Clinical cancer research. 2002;8(8):2576–2579. 12171886

[77] Heller G, McCormack R, Kheoh T, Molina A, Smith MR, Dreicer R, et al Circulating tumor cell number as a response measure of prolonged survival for metastatic castration-resistant prostate cancer: a comparison with prostate-specific antigen across five randomized phase III clinical trials. Journal of Clinical Oncology. 2018;36(6):572 10.1200/JCO.2017.75.2998 29272162 PMC5815402

[78] Scher HI, Heller G, Molina A, Attard G, Danila DC, Jia X, et al Circulating tumor cell biomarker panel as an individual-level surrogate for survival in metastatic castration-resistant prostate cancer. Journal of clinical oncology. 2015;33(12):1348 10.1200/JCO.2014.55.3487 25800753 PMC4397279

[79] Koo KM, Mainwaring PN, Tomlins SA, Trau M. Merging new-age biomarkers and nanodiagnostics for precision prostate cancer management. Nature Reviews Urology. 2019;16(5):302–317. 10.1038/s41585-019-0178-2 30962568

[80] Fox JJ, Gavane SC, Blanc-Autran E, Nehmeh S, Gönen M, Beattie B, et al Positron emission tomography/computed tomography–based assessments of androgen receptor expression and glycolytic activity as a prognostic biomarker for metastatic castration-resistant prostate cancer. JAMA oncology. 2018;4(2):217–224. 10.1001/jamaoncol.2017.3588 29121144 PMC6231549

[81] Evans J, Qiu M, MacKinnon M, Green E, Peterson K, Kaizer L. A multi-method review of home-based chemotherapy. European journal of cancer care. 2016;25(5):883–902. 10.1111/ecc.12408 26545409

[82] Pacheco JM, Santos FC, Dingli D. The ecology of cancer from an evolutionary game theory perspective. Interface focus. 2014;4(4):20140019 10.1098/rsfs.2014.0019 25097748 PMC4071510

[83] Swierniak A, Krzeslak M, Borys D, Kimmel M. The role of interventions in the cancer evolution–an evolutionary games approach. Mathematical Biosciences and Engineering. 2019;16(1):265–291. 10.3934/mbe.2019014 30674120

[84] Jolly MK, Kulkarni P, Weninger K, Orban J, Levine H. Phenotypic plasticity, bet-hedging, and androgen independence in prostate cancer: Role of non-genetic heterogeneity. Frontiers in oncology. 2018;8:50 10.3389/fonc.2018.00050 29560343 PMC5845637

[85] Nam A, Mohanty A, Bhattacharya S, Kotnala S, Achuthan S, Hari K, et al Suppressing chemoresistance in lung cancer via dynamic phenotypic switching and intermittent therapy. bioRxiv. 2020;.

[86] Kumar N, Cramer GM, Dahaj SAZ, Sundaram B, Celli JP, Kulkarni RV. Stochastic modeling of phenotypic switching and chemoresistance in cancer cell populations. Scientific reports. 2019;9(1):1–10. 31350465 10.1038/s41598-019-46926-x PMC6659620

[87] Craig M, Kaveh K, Woosley A, Brown AS, Goldman D, Eton E, et al Cooperative adaptation to therapy (CAT) confers resistance in heterogeneous non-small cell lung cancer. PLoS computational biology. 2019;15(8):e1007278 10.1371/journal.pcbi.1007278 31449515 PMC6709889

[88] Strauss SY. Ecological and evolutionary responses in complex communities: implications for invasions and eco-evolutionary feedbacks. Oikos. 2014;123(3):257–266. 10.1111/j.1600-0706.2013.01093.x

[89] Lankau RA. Rapid Evolutionary Change and the Coexistence of Species. Annual Review of Ecology, Evolution, and Systematics. 2011;42(1):335–354. 10.1146/annurev-ecolsys-102710-145100

[90] Koch H, Frickel J, Valiadi M, Becks L. Why rapid, adaptive evolution matters for community dynamics. Frontiers in Ecology and Evolution. 2014;2:17 10.3389/fevo.2014.00017

[91] Świerniak A, Krześlak M. Cancer heterogeneity and multilayer spatial evolutionary games. Biology direct. 2016;11(1):53 10.1186/s13062-016-0156-z 27737715 PMC5064968

[92] Cleveland C, Liao D, Austin R. Physics of cancer propagation: A game theory perspective. AIP advances. 2012;2(1):011202 10.1063/1.3699043 PMC3321518 22489277

[93] Nanda M, Durrett R. Spatial evolutionary games with weak selection. Proceedings of the National Academy of Sciences. 2017;114(23):6046–6051. 10.1073/pnas.1620852114 PMC5468649 28533405

[94] Kaznatcheev A, Scott JG, Basanta D. Edge effects in game-theoretic dynamics of spatially structured tumours. Journal of The Royal Society Interface. 2015;12(108):20150154 10.1098/rsif.2015.0154 PMC4528581 26040596

[95] Jackson T. A mathematical model of prostate tumor growth and androgen-independent relapse. Discrete & Continuous Dynamical Systems-B. 2004;4(1):187 10.3934/dcdsb.2004.4.187

[96] Jackson TL. A mathematical investigation of the multiple pathways to recurrent prostate cancer: comparison with experimental data. Neoplasia (New York, NY). 2004;6(6):697 10.1593/neo.04259 PMC1531673 15720795

[97] Ideta AM, Tanaka G, Takeuchi T, Aihara K. A mathematical model of intermittent androgen suppression for prostate cancer. Journal of nonlinear science. 2008;18(6):593 10.1007/s00332-008-9031-0

[98] Shimada T, Aihara K. A nonlinear model with competition between prostate tumor cells and its application to intermittent androgen suppression therapy of prostate cancer. Mathematical biosciences. 2008;214(1-2):134–139. 10.1016/j.mbs.2008.03.001 18420226

[99] Tao Y, Guo Q, Aihara K. A mathematical model of prostate tumor growth under hormone therapy with mutation inhibitor. Journal of nonlinear science. 2010;20(2):219–240. 10.1007/s00332-009-9056-z

[100] Jain HV, Clinton SK, Bhinder A, Friedman A. Mathematical modeling of prostate cancer progression in response to androgen ablation therapy. Proceedings of the National Academy of Sciences. 2011;108(49):19701–19706. 10.1073/pnas.1115750108 PMC3241775 22106268

[101] Basanta D, Scott JG, Fishman MN, Ayala G, Hayward SW, Anderson AR. Investigating prostate cancer tumour–stroma interactions: clinical and biological insights from an evolutionary game. British Journal of Cancer. 2012;106(1):174–181. 10.1038/bjc.2011.517 22134510 PMC3251863

[102] Gallaher J, Cook LM, Gupta S, Araujo A, Dhillon J, Park JY, et al Improving treatment strategies for patients with metastatic castrate resistant prostate cancer through personalized computational modeling. Clinical & experimental metastasis. 2014;31(8):991–999. 10.1007/s10585-014-9674-1 25173680 PMC5399888

[103] Hirata Y, Morino K, Akakura K, Higano CS, Aihara K. Personalizing androgen suppression for prostate cancer using mathematical modeling. Scientific reports. 2018;8(1):1–8. 10.1038/s41598-018-20788-1 29422657 PMC5805696

[104] Viossat Y, Noble R. The logic of containing tumors. bioRxiv. 2020;. 10.1101/2020.01.22.915355

