# Supplementary material for: Optimal control to reach eco-evolutionary stability in metastatic castrate-resistant prostate cancer
Source: PLoS One. 2020 Dec 8;15(12):e0243386. doi: 10.1371/journal.pone.0243386 (PMC7723267; doi:10.1371/journal.pone.0243386)
Supplement: S1 File — (ZIP) [file pone.0243386.s001.zip › SupportingInformation/SupportingInformation.pdf]

Supporting Information

S1 Competition Coefficients Background

Prior to and independent of the patient trial, the values of the competition matrix were approximated through a series of inequalities. These inequalities were derived from the literature [1] and professional judgment of prostate oncologists. Two general rules determine the relative values of inter-cell type interactions; (1) T^+ cells with no exogenous testosterone are in general the least competitive cell type, and (2) the competitive effect of T^- cells is stronger on T^P cells than on T^+ cells. In this way, the following six inequalities are considered:

$$\alpha_{31} > \alpha_{21}$$
$$\alpha_{32} > \alpha_{12}$$
$$\alpha_{13} > \alpha_{23}$$
$$\alpha_{13} > \alpha_{12}$$
$$\alpha_{23} > \alpha_{21}$$
$$\alpha_{32} > \alpha_{31}$$

There are 22 different rank orderings of the competition coefficients that satisfy these six inequality conditions. For initial analysis, the values for the ranked α_{ij} coefficients were set to $\{0.4, 0.5, 0.6, 0.7, 0.8, 0.9\}$. For these 22 resulting matrices and growth rates $r_{T^+} = 2.7726 \cdot 10^{-3}$, $r_{T^P} = 3.4657 \cdot 10^{-3}$, $r_{T^-} = 6.6542 \cdot 10^{-3}$ we perform stability analysis with no abiraterone to obtain equilibrium points which occur when the right-hand side expressions in (1) are all zero, while all x_i's are non-negative. The system dynamics (1) with carrying capacities for T^P and T^+ given by Eqs (2) and (3), respectively, is undefined for zero T^P population. Therefore, if an equilibrium point has one or more values of x_i equal to 0, a more refined analysis must be performed, disregarding infeasible direction. We let 0+ denote the right limit to 0.

The stability of the equilibrium points is calculated using eigenvalue analysis of the Jacobian J. The Jacobian (i.e., the matrix of first-order partial derivatives of the right-hand side expressions in (1) with respect to the variables x_i) of the system (1) with carrying capacities for T^P and T^+ given by Eqs (2) and (3), respectively, has the following form:

$$J = \begin{pmatrix} r_{T^+}\left(\frac{x_{T^+}+\alpha_{12}\,x_{T^P}+\alpha_{13}\,x_{T^-}}{x_{T^P}\left(\Lambda-\frac{3}{2}\right)}+1\right)+\frac{r_{T^+}\,x_{T^+}}{x_{T^P}\left(\Lambda-\frac{3}{2}\right)} & -r_{T^+}\,x_{T^+}\left(\frac{x_{T^+}+\alpha_{12}\,x_{T^P}+\alpha_{13}\,x_{T^-}}{{x_{T^P}}^2\left(\Lambda-\frac{3}{2}\right)}-\frac{\alpha_{12}}{x_{T^P}\left(\Lambda-\frac{3}{2}\right)}\right) & \frac{\alpha_{13}\,r_{T^+}\,x_{T^+}}{x_{T^P}\left(\Lambda-\frac{3}{2}\right)} \\ \frac{\alpha_{21}\,r_{T^P}\,x_{T^P}}{9900\,\Lambda-10000} & r_{T^P}\left(\frac{x_{T^P}+\alpha_{21}\,x_{T^+}+\alpha_{23}\,x_{T^-}}{9900\,\Lambda-10000}+1\right)+\frac{r_{T^P}\,x_{T^P}}{9900\,\Lambda-10000} & \frac{\alpha_{23}\,r_{T^P}\,x_{T^P}}{9900\,\Lambda-10000} \\ -\frac{\alpha_{31}\,r_{T^-}\,x_{T^-}}{K_{T^-}} & -\frac{\alpha_{32}\,r_{T^-}\,x_{T^-}}{K_{T^-}} & -r_{T^-}\left(\frac{x_{T^-}+\alpha_{31}\,x_{T^+}+\alpha_{32}\,x_{T^P}}{K_{T^-}}-1\right)-\frac{r_{T^-}\,x_{T^-}}{K_{T^-}} \end{pmatrix} \quad (10)$$

Based on the frequency of the resistant T^- cells at the stable point for each of the 22 matrices, we can divide the 22 cases into three different groups: best-responders, responders, and non-responders. Sensitivity analysis of these stable points and categories by varying any one competition coefficient is discussed in detail in [49]. This analysis shows that the exact value of the competition coefficient when within [0,1] doesn't vary the stable points all that much. What is of most interest is the rank ordering itself.

Empirically, some men do not show a response to abiraterone and would presumably have matrices within the non-responder category. Furthermore, it is rare if non-existent to see men cured by abiraterone as would be the case with the the patients with best-responder matrices. In this way, these two groups are somewhat trivial for analysis here. On the other hand the responder group containing four possible rank orderings is of particular interest.

- Non-responders: Six matrices result in high frequencies of T^- at the stable point with frequencies of T^- ($\geq 20\%$). These competition matrices do not respond to

therapy and do not result in even a 50% initial drop in tumor burden as measured by PSA.

- Best responders: Twelve of the 22 possible competition matrices promote an absence of T^- at the stable point. These tumor types should respond well to therapy with large and relatively sustained drops in PSA.

- Responders: Four matrices result in low frequencies of T^- at the stable point. When simulating standard of care, the initial response is expected to be strong but unsustainable as T^- quickly increases in frequency and results in progression. The four matrices are shown in table S1.

Matrix	coefficient values					
	α_{12}	α_{13}	α_{21}	α_{23}	α_{31}	α_{32}
(A)	0.7	0.8	0.4	0.6	0.5	0.9
(B)	0.6	0.8	0.4	0.7	0.5	0.9
(C)	0.7	0.9	0.4	0.5	0.6	0.8
(D)	0.5	0.9	0.4	0.7	0.6	0.8

Table S1. Responder matrix coefficients Four responder matrices identified from the analysis of the original 22 possible parameter orderings that satisfy the biological inequalities.

S2 Stability Analysis for Constant But Varying Doses of Abiraterone

For these four matrices, stability analysis is performed for different but constant values of $\Lambda(\cdot) \in [0, 1]$ as we are interested in situations where tumor burden can be maintained using a fixed amount of medication. If such an equilibrium exists and is asymptotically stable, it will have some robustness against stochastic variations such as tumor composition and actual delivered dose. Furthermore, this allows us to apply piecewise constant control laws, where we choose between just a few different levels of abiraterone dose that are clinically available, i.e. $\Lambda(t) \in \{0.1, 0.2, 0.3, \ldots, 1\}$. In Table S1 the stable points for each of the four matrices is presented as a function of abiraterone dose $\Lambda(\cdot) \in [0, 1]$. [50], [49], and here show that no stable equilibria exist with these original competition coefficients where the patient tumor burden is below the viability constraint (≤ 9000).

Fig S1. Stable points for the four responder matrices. There are no regions where the stable points are within the patient viability constraint where the total volume at that stability point is ≤ 9000.

S3 Adjusting Competition Matrices

While the initial assumptions of the model required the competition coefficients to be within $[0, 1]$, evolutionary competition studies have shown that competing cancer cells may result in competition coefficients that are far greater than one [28, 34]. In this way, we relax the constraint on the competition coefficients having values less than one and identify which competition coefficients are most pertinent to stability for these four competition matrices. To identify stable points that exist within the patient viability constraint that include T^+ and T^P cells, we set $\dot{x}_{T^-} \leq 0$. Rearranging the state equation for x_3 gives the following.

$$\dot{x}_3 = x_3 r_3 \left(1 - \frac{\alpha_{31}x_1 + \alpha_{32}x_2 + x_3}{K_3}\right)$$

Setting $\dot{x}_3 \leq 0$.

$$x_3 r_3 \left(1 - \frac{\alpha_{31}x_1 + \alpha_{32}x_2 + x_3}{K_3}\right) \leq 0$$

$$1 - \frac{\alpha_{31}x_1 + \alpha_{32}x_2 + x_3}{K_3} \leq 0$$

$$1 \leq \frac{\alpha_{31}x_1 + \alpha_{32}x_2 + x_3}{K_3}$$

$$K_3 \leq \alpha_{31}x_1 + \alpha_{32}x_2 + x_3$$

$$K_3 - x_3 \leq \alpha_{31}x_1 + \alpha_{32}x_2$$

Under the patient viability constraint where

$$\sum_{i \in \mathcal{T}} x_i \leq 9000 \tag{11}$$

we see that the competition coefficients α_{31} and α_{32} are the key parameters affecting stability for all values of x_3. Specifically, α_{31} and/or α_{32} must be greater than one. α_{31} and α_{32} are the competition of T^+ and T^P cells on T^- cells respectively. To see how the increase of α_{32} affects the stable points we show a sensitivity analysis of increasing α_{32} for Matrix A (the matrix explored in detail within the main text). Fig S2 graphically displays this stability analysis for $\alpha_{32} = 1.0$, $\alpha_{32} = 1.1$, $\alpha_{32} = 2.0$, and $\alpha_{32} = 5.0$.

Fig S2. Stable points for various values of α in matrix A. The dark highlighted regions show the stable points that are within the patient viability constraint where the total volume at that stability point is ≤ 9000. (A) Stable points for Matrix A with $\alpha_{32} = 1.0$. (B) Stable points for Matrix A with $\alpha_{32} = 1.1$ (C) Stable points for Matrix A with $\alpha_{32} = 2.0$. (D) Stable points for Matrix A with $\alpha_{32} = 5.0$.

Fig S2 shows that increasing the value of α_{32} shifts the stable points. The values of the competition coefficients being less than or equal to one require high cell densities to elicit a strong competition between cell types, specifically the T^- cells, which makes all stable points have total volumes above the patient viability constraint. As α_{32} increases, the stable points shift, allowing the same dose of abiraterone to maintain lower levels of T^-, along with some stability points where T^- is excluded.

The case where $\alpha_{32} = 2.0$ is the matrix discussed in detail in the main text of the paper. We choose $\alpha_{32} = 2.0$, because it 1) allows for stability points within the patient viability constraint and 2) did not eliminate T^- from all stability points (like that seen with $\alpha_{32} = 5.0$) so we could analyze a case where the resistant T^- cells are still present in the tumor, which would be expected clinically.

Obviously, the exact value of any of the competition coefficients will affect the behaviour and stability of this model. Experimental work to identify the exact, or at least probable, values of these coefficients will greatly improve the validity of the model predictions. The work provided here just assumes that there is a stable heterogeneous stable point of any kind within the patient tumor burden. There are many ways to achieve this, and we will show a few examples beyond that shown in the main text here. Until experimental work is provided, the values chosen here provide a starting point for further analysis.

For Matrix B, increasing the value of $\alpha_{32} = 2.0$ introduced a region of stable points that include only T^+ and T^P cells shown in Fig S3.

Fig S3. Stable points for Matrix B $\alpha_{32} = 2.0$. The dark highlighted regions show the stable points that are within the patient viability constraint where the total volume at that stability point is ≤ 9000.

Interestingly, for matrices C and D (Fig S4), in order to increase the value of α_{32} above 1.0 while maintaining the inequalities, α_{13} (the competition of T^- cells on T^+ cells) also had be increased. In this way the value of α_{13} is set to 2.0 and α_{32} is set to 1.5. Because the T^- cells now have a significant competitive affect on T^+ cells, the stability points that contain T^- cells have quite low to zero densities of T^+ cells.

Fig S4. Stable points for Matrix C and D with increased coefficients. The dark highlighted regions show the stable points that are within the patient viability constraint where the total volume at that stability point is ≤ 9000. (A) Stable points for Matrix C $\alpha_{13} = 2.0$ and $\alpha_{32} = 1.5$. (B) Stable points for Matrix D $\alpha_{13} = 2.0$ and $\alpha_{32} = 1.5$.

A stable point from every stable region from each of the updated matrices is displayed in Table S2. The stable points for Matrix A are discussed in detail in the main text, while the remaining stable points are discussed in further supplemental sections.

	coefficient values								
Matrix	α_{12}	α_{13}	α_{21}	α_{23}	α_{31}	α_{32}	Λ	Stable Point for FBS	Volume
(A)	0.7	0.8	0.4	0.6	0.5	2.0	0.4	(2082.76, 5206.90, 0.00)	7290
							0.4848	(863.45,4436.73,694.82)	5995
(B)	0.6	0.8	0.4	0.7	0.5	2.0	0.39	(2600.00,5099.00,0.00)	7699
(C)	0.7	2.0	0.4	0.5	0.6	1.5	0.4	(0.0, 3764, 4354)	8118
							0.35	(1003, 5739,790)	7532
							0.31	(2840,5795,0)	8635
(D)	0.5	2.0	0.4	0.7	0.6	1.5	0.33	(3558,5310,0.00)	8868

Table S2. All stable points used for further analysis. The matrix label, updated coefficients, abiraterone dose associated with the stable point, the density of each of the subpopulations at the stable point, and the total tumor volume are shown. A total of 7 stable points are available for discussion.

S4 Forwards Backwards Sweep Algorithm

The Forward Backward Sweep (FBS) algorithm is a method designed to solve the differential-algebraic system generated by Pontryagin's Maximum Principle [2,3]. The Maximum Principle in optimal control theory states that there is a co-state variable $\Lambda_i(t)$ where an optimal state $x_i(t)$ and the optimal control $\Lambda^*(t)$ must satisfy the dynamics of the state equations where $i \in \mathcal{T} = \{T^+, T^P, T^-\}$ given by

$$\begin{cases} \dot{x_i} = r_i x_i \left(1 - \frac{\sum_{j \in \mathcal{T}} \alpha_{ij} x_j}{K_i}\right) \\ x_i(t_0) = x_i^0 \end{cases}$$

Initial conditions are given by the 100 random initial tumor compositions $(x_{T^+}(t_0), x_{T^P}(t_0), x_{T^-}(t_0))$, shown in Fig 2. The two-dimensional projections of these initial tumor compositions are shown if Fig S5.

Fig S5. Two-dimensional projections for 100 random initial states used by FBS. Projections of the 100 random initial states $x(t_0)$ to x^* with $\Lambda^*(\cdot)$ used by FBS. (x_{T^+}, x_{T^P})-space in the left panel, (x_{T^+}, x_{T^-})-space in the center panel, and (x_{T^P}, x_{T^-})-space in the right panel.

It is also required that costates $\lambda_i(t)$ satisfy the transversality conditions given by

$$\begin{cases} \dot{\lambda}_i = -\frac{\partial H}{\partial x_i} \\ \lambda_i(t_f) = 0 \end{cases}$$

where co-state equations λ_i for the Hamiltonian in (9) are constructed below.

$$\begin{aligned} \dot{\lambda}_{T^+} &= \frac{x_{T^+} - x^*_{T^+}}{\phi} - \lambda_{T^+}\, r_{T^+} \left(\frac{x_{T^+} + \alpha_{12}\, x_{T^P} + \alpha_{13}\, x_{T^-}}{K_{T^+}(\Lambda)\, x_{T^P}} - 1 \right) \dots \\ &\quad - \frac{\lambda_{T^+}\, r_{T^+}\, x_{T^+}}{K_{T^+}(\Lambda)\, x_{T^P}} - \frac{\alpha_{21}\, \lambda_{T^P}\, r_{T^P}\, x_{T^P}}{K_{T^P}(\Lambda)} - \frac{\alpha_{31}\, \lambda_{T^-}\, r_{T^-}\, x_{T^-}}{K_{T^-}} \end{aligned} \tag{12}$$

$$\begin{aligned} \dot{\lambda}_{T^P} &= \frac{x_{T^P} - x^*_{T^P}}{\phi} - \lambda_{T^P}\, r_{T^P} \left(\frac{\alpha_{21}\, x_{T^+} + x_2 + \alpha_{23}\, x_{T^-}}{K_{T^P}} - 1 \right) \dots \\ &\quad - \lambda_{T^+}\, r_{T^+}\, x_{T^+} \left(\frac{\alpha_{12}}{K_{T^+}(\Lambda)\, x_{T^P}} - \frac{x_{T^+} + \alpha_{12}\, x_{T^P} + \alpha_{13}\, x_{T^-}}{K_{T^+}(\Lambda)\, {x_{T^P}}^2} \right) - \dots \\ &\quad \frac{\lambda_{T^P}\, r_{T^P}\, x_{T^P}}{K_{T^P}(\Lambda)} - \frac{\alpha_{32}\, \lambda_{T^-}\, r_{T^-}\, x_{T^-}}{K_{T^-}} \end{aligned} \tag{13}$$

$$\begin{aligned} \dot{\lambda}_{T^-} &= \frac{x_{T^-} - x^*_{T^-}}{\phi} - \lambda_{T^-}\, r_{T^-} \left(\frac{x_{T^-} + \alpha_{31}\, x_{T^+} + \alpha_{32}\, x_{T^P}}{K_{T^-}} - 1 \right) \dots \\ &\quad - \frac{\alpha_{13}\, \lambda_{T^+}\, r_{T^+}\, x_{T^+}}{K_{T^+}(\Lambda)\, x_{T^P}} - \frac{\alpha_{23}\, \lambda_{T^P}\, r_{T^P}\, x_{T^P}}{K_{T^P}(\Lambda)} - \frac{\lambda_{T^-}\, r_{T^-}\, x_{T^-}}{K_{T^-}} \end{aligned} \tag{14}$$

where

$$\phi = \sqrt{\left(x_{T^+} - x^*_{T^+}\right)^2 + \left(x_{T^P} - x^*_{T^P}\right)^2 + \left(x_{T^-} - x^*_{T^-}\right)^2} \tag{15}$$

The first iteration of the FBS algorithm requires as input an initial guess of the control $\Lambda(\cdot)$ which for the simulations presented here is set to $\Lambda(t) = 0.5$ for all $t \in [t_0, t_f]$. For each iteration the initial value problem of the state equation is solved forward in time subject to the initial conditions set by the initial tumor composition $(x_{T^+}(t_0), x_{T^P}(t_0), x_{T^-}(t_0))$. Then the co-state final value problem is solved backwards in time subject to the transversality conditions $\lambda_i(t_f) = 0$. The solution to these are then used solve for the Hamiltonian given in (9). As optimal control attempts to maximizes the Hamiltonian given in (9), the new guess for the optimal $\Lambda(t)$, denoted here by $\Lambda^{\circ(n+1)}$, is calculated using the following formula.

$$\Lambda^{\circ(n+1)} = \Lambda^{\circ(n)} - \frac{\partial H}{\partial \Lambda^{\circ(n)}} \tag{16}$$

where

$$\begin{aligned} \frac{\partial H}{\partial \Lambda} &= \frac{\lambda_{T^P}\, r_{T^P}\, x_{T^P}\, \left(\alpha_{21}\, x_{T^+} + x_{T^P} + \alpha_{23}\, x_{T^-}\right)}{K_{T^P}(\Lambda)^2} + \dots \\ &\quad \frac{\lambda_{T^+}\, r_{T^+}\, x_{T^+}\, \left(x_{T^+} + \alpha_{12}\, x_{T^P} + \alpha_{13}\, x_{T^-}\right)}{10000\, x_{T^P} \left(\frac{K_{T^P}(\Lambda)}{10000} + \frac{x_{T^+}}{2} \right)^2} \end{aligned} \tag{17}$$

also known as a switching manifold. The stopping criteria for this iterative algorithm is determined by finding the relative error of the control variable and requiring that it be less than a tolerance $\delta = 10^{-4}$. In this way the algorithm terminates when

$$\frac{\|\Lambda^{\circ(n-1)} - \Lambda^{\circ(n)}\|}{\|\Lambda^{\circ(n)}\|} < \delta \tag{18}$$

where $\|\cdot\|$ is the ℓ^1-norm, $\|\Lambda^{\circ(n)}\| = \sum_{j=1}^{t_f} |\Lambda(j)^{\circ(n)}|$. The initial guess of the optimal treatment is given by $\Lambda^{\circ(1)} = 0.5$. The vector $\Lambda^{\circ(n)}$ is then used as the optimized treatment protocol.

S5 All Optimal Treatments

The Forwards Backwards Sweep algorithm is used for each of the seven stability points provided in S2. The optimal abiraterone dosing schedule to arrive at the stable point

from each of the 100 starting points is shown in Fig S6 - Fig S12.

Fig S6. Forwards backwards sweep results for Matrix A to two-species stability point. Matrix A Forward Backwards Sweep results for optimal dosing schedule to arrive at two-species stability point $(2082.76, 5206.90, 0.00)$.

Fig S7. Forwards backwards sweep results for Matrix A to three-species stability point. Matrix A Forward Backwards Sweep results for optimal dosing schedule to arrive at three-species stability point $(863.45, 4436.73, 694.82)$.

Fig S8. Forwards backwards sweep results for Matrix B to two-species stability point. Matrix B Forward Backwards Sweep results for optimal dosing schedule to arrive at two-species stability point $(2600.00, 5099.00, 0.00)$.

Fig S9. Forwards backwards sweep results for Matrix C to two-species stability point. Matrix C Forward Backwards Sweep results for optimal dosing schedule to arrive at two-species stability point $(0.0, 3764, 4354)$.

Fig S10. Forwards backwards sweep results for Matrix C to three-species stability point. Matrix C Forward Backwards Sweep results for optimal dosing schedule to arrive at three-species stability point $(1003, 5739, 790)$.

Fig S11. Forwards backwards sweep results for Matrix C to two-species stability point. Matrix C Forward Backwards Sweep results for optimal dosing schedule to arrive at two-species stability point $(2840, 5795, 0)$.

Fig S12. Forwards backwards sweep results for Matrix D to two-species stability point. Matrix D Forward Backwards Sweep results for optimal dosing schedule to arrive at two-species stability point $(3558, 5310, 0.00)$.

S6 Optimal Path Projections

Projections for FBS results for Matrix A to the two-species equilibrium are shown in Fig S13.

Fig S13. Two-dimensional projections of state dynamics from FBS for Matrix A to the two-species equilibrium. Projections of the state trajectories from 100 random initial states $x(t_0)$ to x^* with $\Lambda^*(\cdot)$ found by FBS to the two species equilibria. (x_{T^+}, x_{T^P})-space in the left panel, (x_{T^+}, x_{T^-})-space in the center panel, and (x_{T^P}, x_{T^-})-space in the right panel. Paths highlighted in red breach the patient viability constraint before reaching the equilibrium point.

Projections for FBS results for Matrix A to the three-species equilibrium are shown in Fig S14.

Fig S14. Two-dimensional projections of state dynamics from FBS for Matrix A to the three-species equilibrium. Projections of the state trajectories from 100 random initial states $x(t_0)$ to x^* with $\Lambda^*(\cdot)$ found by FBS to the three species equilibria. (x_{T^+}, x_{T^P})-space in the left panel, (x_{T^+}, x_{T^-})-space in the center panel, and (x_{T^P}, x_{T^-})-space in the right panel. Paths highlighted in red breach the patient viability constraint before reaching the equilibrium point.

S7 Comparison of Titration Protocols for One Patient under Matrix A

Fig S15 shows the population dynamics of the same incoming simulated patient under the four titration protocols. The patients incoming tumor volume is 5924.83. Panel (a) attempts to stabilize at V_a with the initial dose as $\Lambda(t_0) = 1$. The high initial doses cause the volume to drop below V_a and in response the dose is titrated down quite quickly. Unfortunately, due to the high initial doses the underlying tumor composition includes a high proportion of T^- cells and stabilization is lost once these cells outgrow the T^P population. Panel (b) attempts to instead stabilize at V_b with the same initial dose of $\Lambda(t_0) = 1$. The same initial dynamics are observed but because the stabilization volume is allowed to be larger than that in panel (a), the T^P population increases enough to out-compete the T^- population. Furthermore, a T^+ population is sustained by the large T^P population and stabilization is achieved.

In panel (c), the target volume is again V_a but the initial dose is now changed to $\Lambda(t_0) = 0$. The dosage is only increased as the T^P and T^+ cells begin to grow, but the competitive release of T^- cells is avoided because no high doses of abiraterone were given. The underlying dynamics show that eventually the T^- cells will take over the tumor population but the total tumor volume does not exceed the patient viability constraint during the simulation. If instead we use V_b, the presence of the large T^P and T^+ population out-compete the T^- population and stabilization is achieved.

Fig S15. Example of the four titration protocols. The population dynamics and associated abiraterone treatment for the four titration protocols for the same example patient. (A) $\Lambda(t_0) = 1$ stabilizing at incoming volume. (B) $\Lambda(t_0) = 0$ stabilizing at incoming volume. (C) $\Lambda(t_0) = 0$ stabilizing at incoming volume. (D) $\Lambda(t_0) = 0$ stabilizing at $V_b = 7000$.

S8 Kaplan Meier Analysis for Additional Matrices

The six clinically feasible protocols discussed in 5 is given to 10,000 simulated patients for each of the four original matrices provided in S1 and the four matrices with increased coefficients provided in S2. In addition to the six protocols, the case where no abiraterone is also shown. In this way, 70,000 simulated patients were run for each of the eight matrices, resulting in 560,000 total patients. The Kaplan Meier survival curve for all 560,000 patients combined is shown in Fig S16. Combining all patients together can help to understand the risk of giving each of the therapies if there is absolutely no information about the underlying biology. While all treatments have a similar median time to progression, the increasing dose titration protocols provide the best overall survival. In particular the increasing dose titration protocol with the stabilizing at a large tumor volume is the best for the cohort of patients. We see that maximum tolerated dose protocol is not ideal.

Fig S16. Kaplan Meier survival curves for all simulated patients. Combined Kaplan Meier survival analysis for all $560,000$ simulated patients under all possible matrices and treatments.

In Fig S17 the Kaplan Meier survival curves for all patients with original matrices and all patients with new matrices separately are shown. Furthermore, in Figs S18-S21, the Kaplan Meier curves are shown for each matrix individually.

Fig S17. Kaplen Meier survival curves for all simulated patients split by original and increased coefficients. Kaplan Meier Survival Analysis for all matrices combined for both the new matrices with increased competition coefficients as well as the KM analysis for the original matrices with all coefficients less than or equal to 1. (A) All matrices with increased coefficients. (B) All matrices with original coefficients.

Fig S18. Kaplen Meier survival curves for Matrix A patients split by original and increased coefficients. Kaplan Meier Survival Analysis for Matrix A for both the new matrices with increased competition coefficients as well as the KM analysis for the original matrices with all coefficients less than or equal to 1. (A) Matrix A with increased coefficients. (B) Matrix A original coefficients. Please note the x-axis limits are only [0, 4000].

Fig S19. Kaplen Meier survival curves for Matrix B patients split by original and increased coefficients. Kaplan Meier Survival Analysis for Matrix B for both the new matrices with increased competition coefficients as well as the KM analysis for the original matrices with all coefficients less than or equal to 1. (A) Matrix B with increased coefficients. (B) Matrix B original coefficients. Please note the x-axis limits are only [0, 4000].

Fig S20. Kaplen Meier survival curves for Matrix C patients split by original and increased coefficients. Kaplan Meier Survival Analysis for Matrix C for both the new matrices with increased competition coefficients as well as the KM analysis for the original matrices with all coefficients less than or equal to 1. (A) Matrix C with increased coefficients. (B) Matrix C original coefficients. Please note the x-axis limits are only [0, 4000].

Fig S21. Kaplen Meier survival curves for Matrix D patients split by original and increased coefficients. Kaplan Meier Survival Analysis for Matrix D for both the new matrices with increased competition coefficients as well as the KM analysis for the original matrices with all coefficients less than or equal to 1. (A) Matrix D with increased coefficients. (B) Matrix D original coefficients. Please note the x-axis limits are only [0, 4000].

S9 Surviving Initial Tumor Compositions for Matrix A

Fig S22 shows the projections of all 10000 initial tumor compositions used in testing the clinically feasible protocols.

Fig S22. Two-dimensional projection of clinically feasible protocol simulations initial conditions. The projections of all 10,000 initial tumor compositions used in testing the clinically feasible protocols. (x_{T^+}, x_{T^P})-space in the left panel, (x_{T^+}, x_{T^-})-space in the center panel, and (x_{T^P}, x_{T^-})-space in the right panel.

Fig S23 shows the projections of 1139 initial tumor compositions that did not breach the patient viability constraint during the simulation under adaptive therapy.

Fig S23. Projections of initial conditions of surviving patients under adaptive therapy. Projections of the initial tumor compositions of the 1139 patients that survived under adaptive therapy. (x_{T^+}, x_{T^P})-space in the left panel, (x_{T^+}, x_{T^-})-space in the center panel, and (x_{T^P}, x_{T^-})-space in the right panel.

Fig S24 shows the projections of 912 initial tumor compositions that did not breach the patient viability constraint during the simulation under titration with $\Lambda(t_0) = 1$ and target volume is the initial tumor volume, V_a.

Fig S24. Projections of initial conditions of surviving patients under titration with $\Lambda(t_0) = 1$ and target volume is the initial tumor volume , V_a. Projections of the initial tumor compositions of the 912 patients that survived under titration with $\Lambda(t_0) = 1$ and target volume is the initial tumor volume, V_a. (x_{T^+}, x_{T^P})-space in the left panel, (x_{T^+}, x_{T^-})-space in the center panel, and (x_{T^P}, x_{T^-})-space in the right panel.

Fig S25 shows the projections of 2420 initial tumor compositions that did not breach the patient viability constraint during the simulation under titration with $\Lambda(t_0) = 0$ and and target volume is the initial tumor volume, V_a.

Fig S25. Projections of initial conditions of surviving patients under titration with $\Lambda(t_0) = 0$ and and target volume is the initial tumor volume, V_a. Projections of the initial tumor compositions of the 2420 patients that survived under titration with $\Lambda(t_0) = 0$ and and target volume is the initial tumor volume, V_a. (x_{T^+}, x_{T^P})-space in the left panel, (x_{T^+}, x_{T^-})-space in the center panel, and (x_{T^P}, x_{T^-})-space in the right panel.

Fig S26 shows the projections of 2039 initial tumor compositions that did not breach the patient viability constraint during the simulation under titration with $\Lambda(t_0) = 1$ and target volume is $V_b = 7000$.

Fig S26. Projections of initial conditions of surviving patients under titration with $\Lambda(t_0) = 1$ and target volume is $V_b = 7000$. Projections of the initial tumor compositions of the 2039 patients that survived under titration with $\Lambda(t_0) = 1$ and target volume is $V_b = 7000$. (x_{T^+}, x_{T^P})-space in the left panel, (x_{T^+}, x_{T^-})-space in the center panel, and (x_{T^P}, x_{T^-})-space in the right panel.

Fig S27 shows the projections of 6555 initial tumor compositions that did not breach the patient viability constraint during the simulation under titration with $\Lambda(t_0) = 0$ and target volume is $V_b = 7000$.

Fig S27. Projections of initial conditions of surviving patients under titration with $\Lambda(t_0) = 0$ and target volume is $V_b = 7000$. Projections of the initial tumor compositions of the 6555 patients that survived under titration with $\Lambda(t_0) = 0$ and target volume is $V_b = 7000$. (x_{T^+}, x_{T^P})-space in the left panel, (x_{T^+}, x_{T^-})-space in the center panel, and (x_{T^P}, x_{T^-})-space in the right panel.

References

1. Werahera PN, Glode LM, La Rosa FG, Lucia MS, Crawford ED, Easterday K, et al. Proliferative tumor doubling times of prostatic carcinoma. Prostate cancer. 2011;2011.

2. Pontryagin LS, Boltianski VG, Gamkrelidze RV, Mishchenko EF, Brown DE. The mathematical theory of optimal processes; 1964. Available from: http://opac.inria.fr/record=b1122221.

3. Metz JAJ, Staňková K, Johansson J. The adaptive dynamics of life histories: From fitness-returns to selection gradients and Pontryagin's maximum principle. Journal of Mathematical Biology. 2016;72(4):1125–1152.
