# Supplementary figures and images for: Optimal control to reach eco-evolutionary stability in metastatic castrate-resistant prostate cancer
Source: PLoS One. 2020 Dec 8;15(12):e0243386. doi: 10.1371/journal.pone.0243386 (PMC7723267; doi:10.1371/journal.pone.0243386)

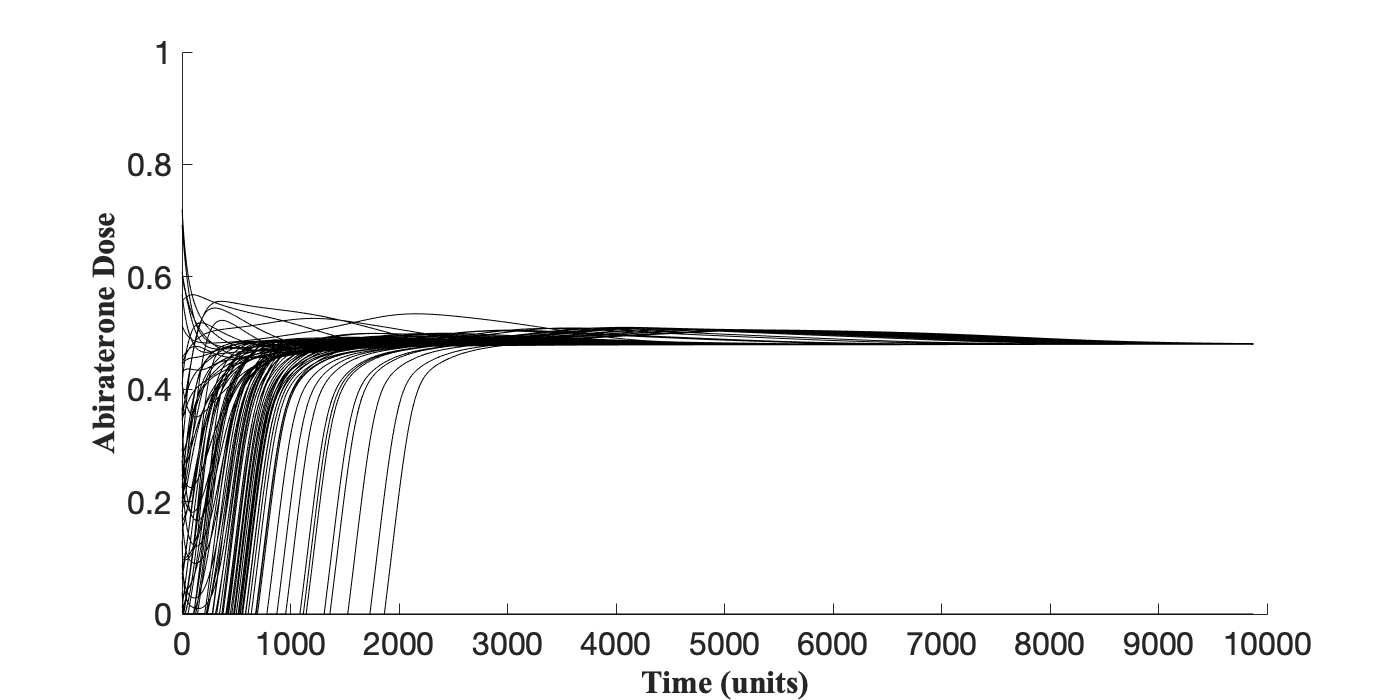

Supplement: S1 File — (ZIP) [file pone.0243386.s001.zip › SupportingInformation/FigureS7.tiff]

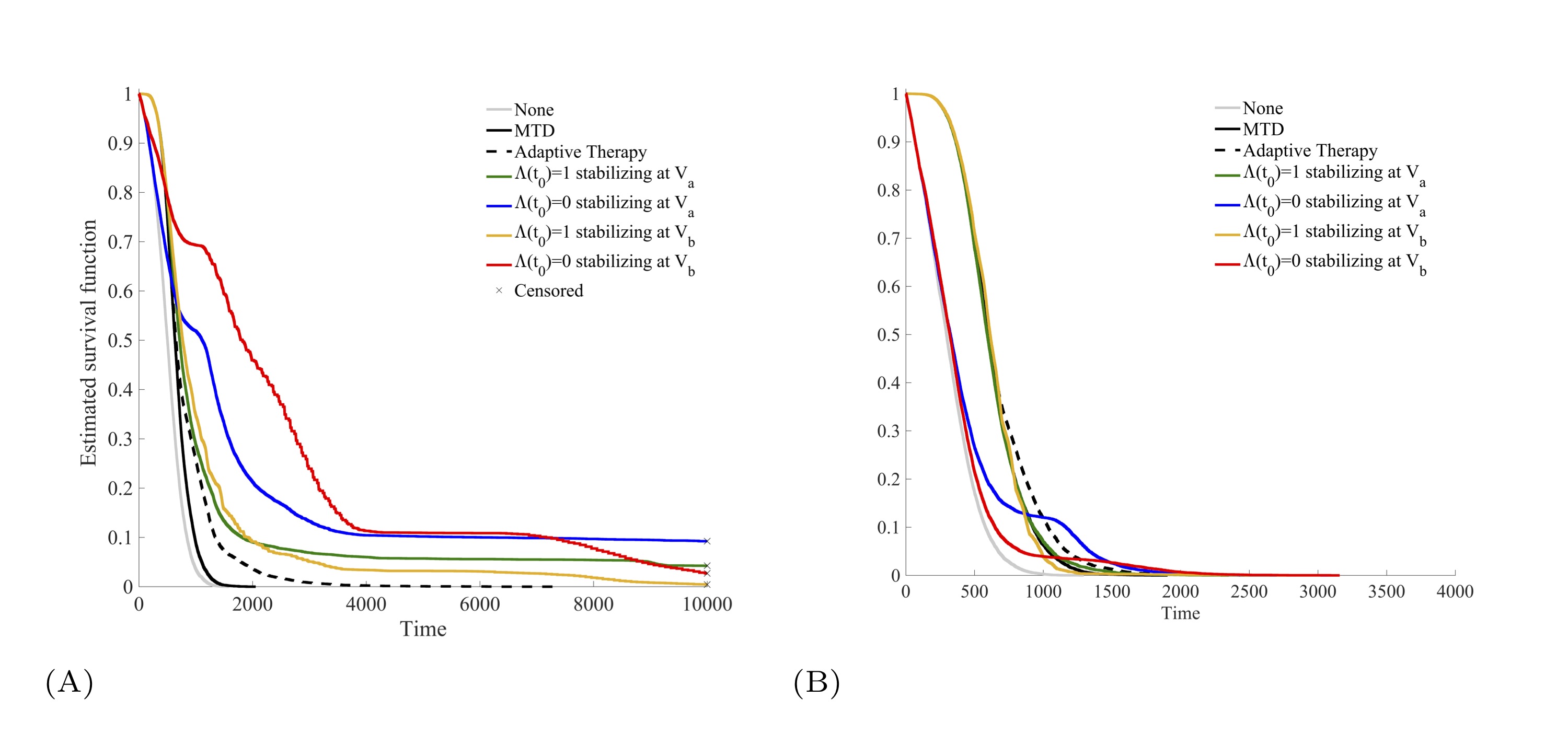

Supplement: S1 File — (ZIP) [file pone.0243386.s001.zip › SupportingInformation/FigureS20.tiff]

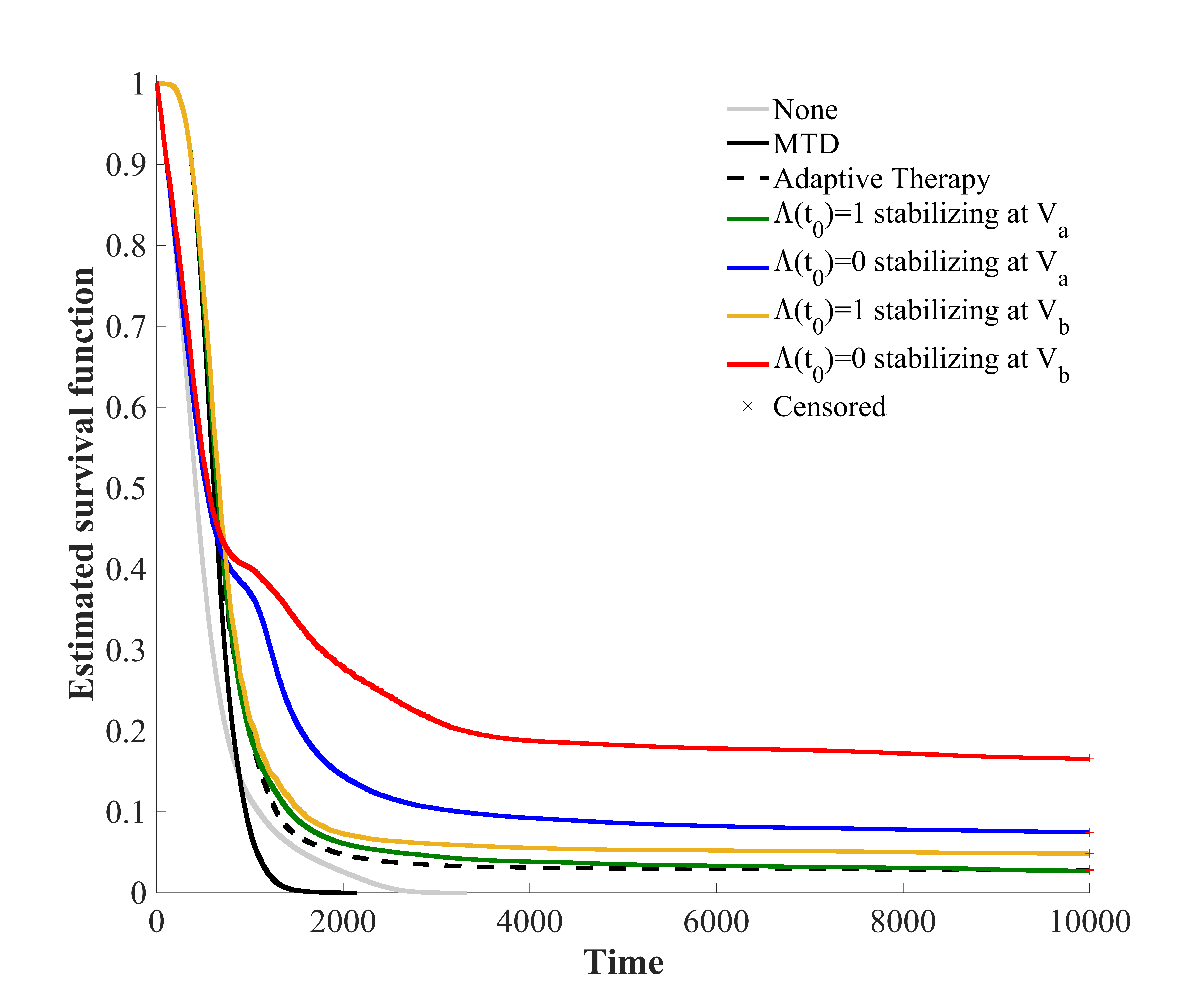

Supplement: S1 File — (ZIP) [file pone.0243386.s001.zip › SupportingInformation/FigureS16.tiff]

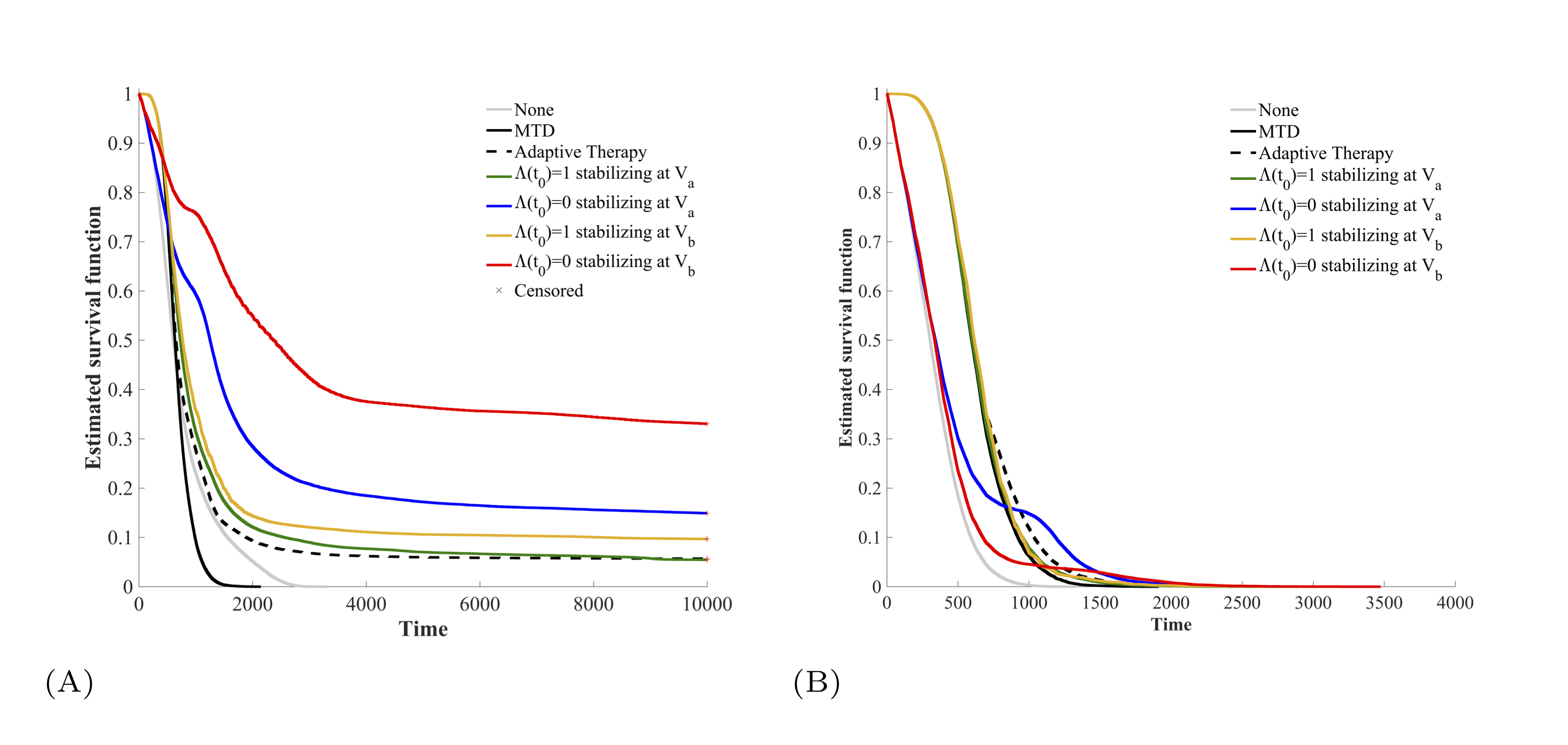

Supplement: S1 File — (ZIP) [file pone.0243386.s001.zip › SupportingInformation/FigureS17.tiff]

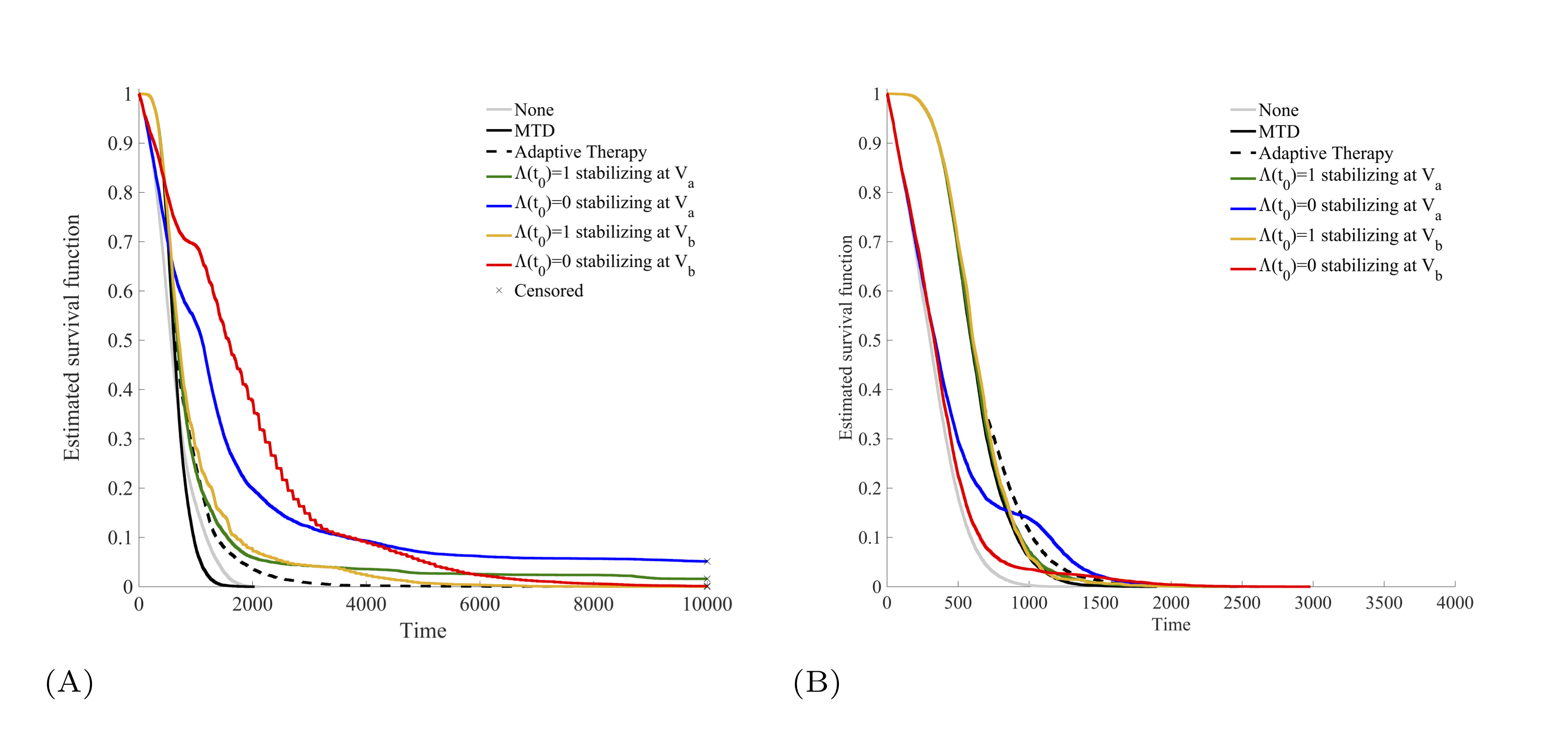

Supplement: S1 File — (ZIP) [file pone.0243386.s001.zip › SupportingInformation/FigureS21.tiff]

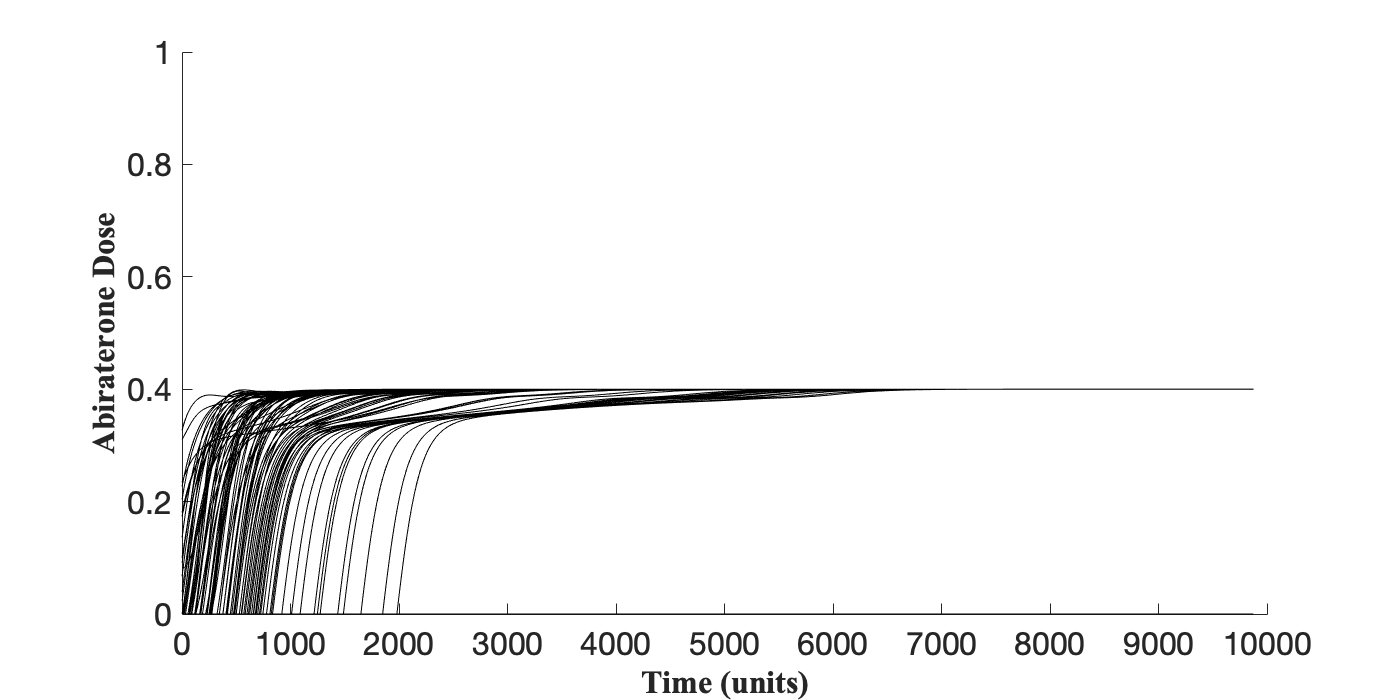

Supplement: S1 File — (ZIP) [file pone.0243386.s001.zip › SupportingInformation/FigureS6.tiff]

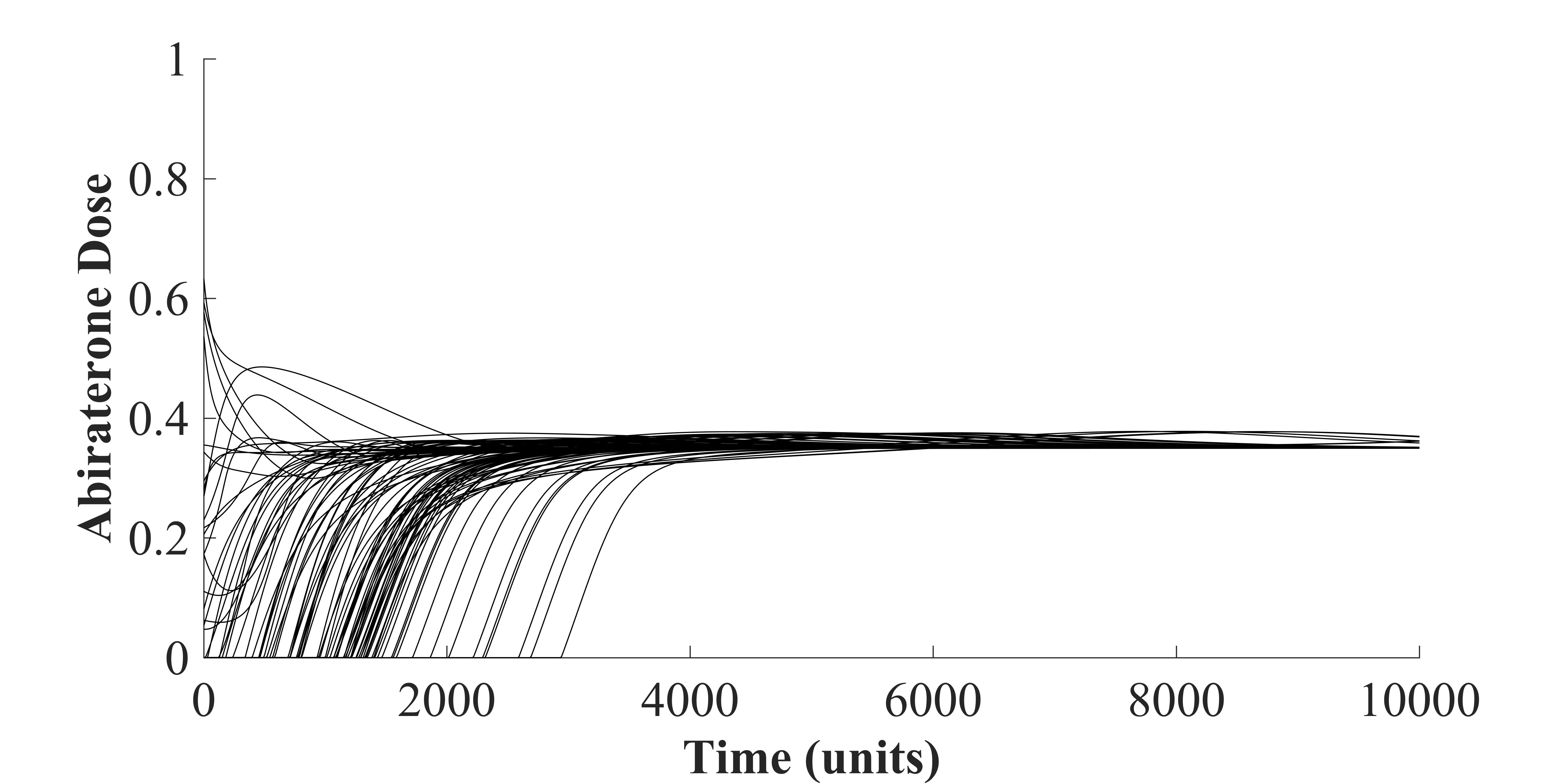

Supplement: S1 File — (ZIP) [file pone.0243386.s001.zip › SupportingInformation/FigureS10.tiff]

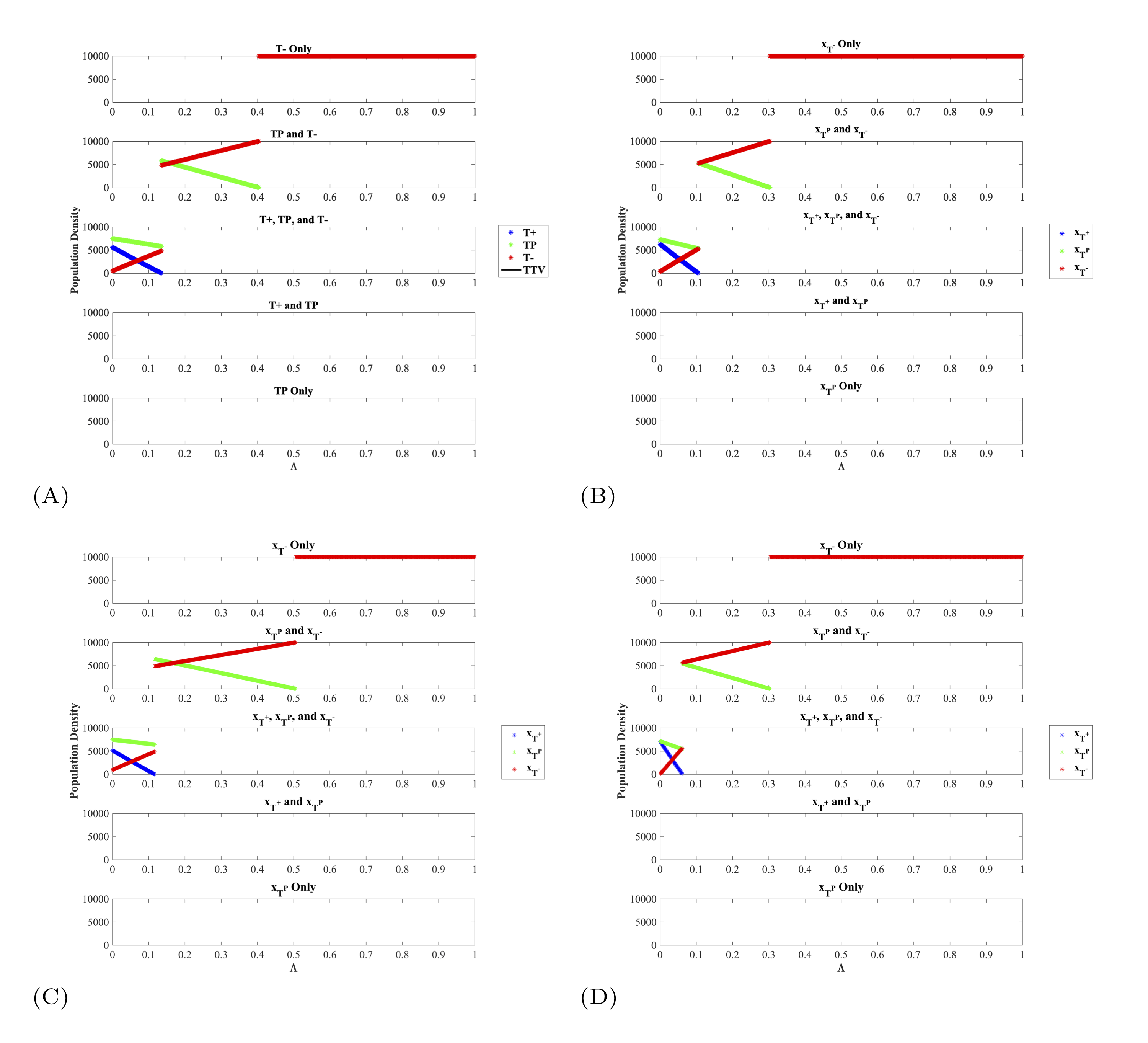

Supplement: S1 File — (ZIP) [file pone.0243386.s001.zip › SupportingInformation/FigureS1.tiff]

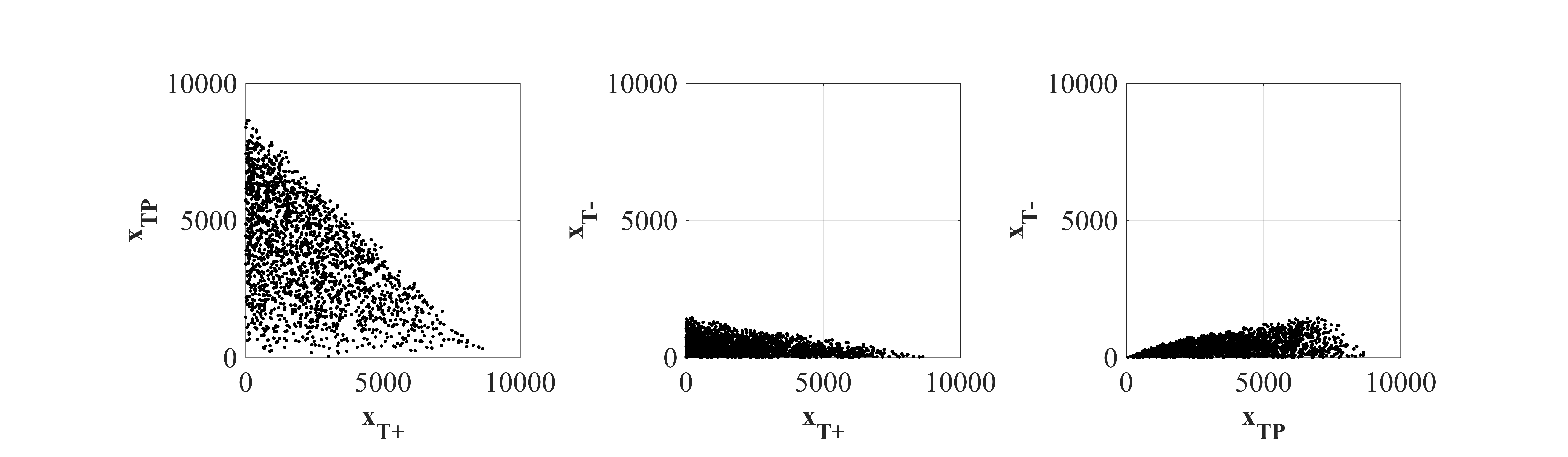

Supplement: S1 File — (ZIP) [file pone.0243386.s001.zip › SupportingInformation/FigureS26.tiff]

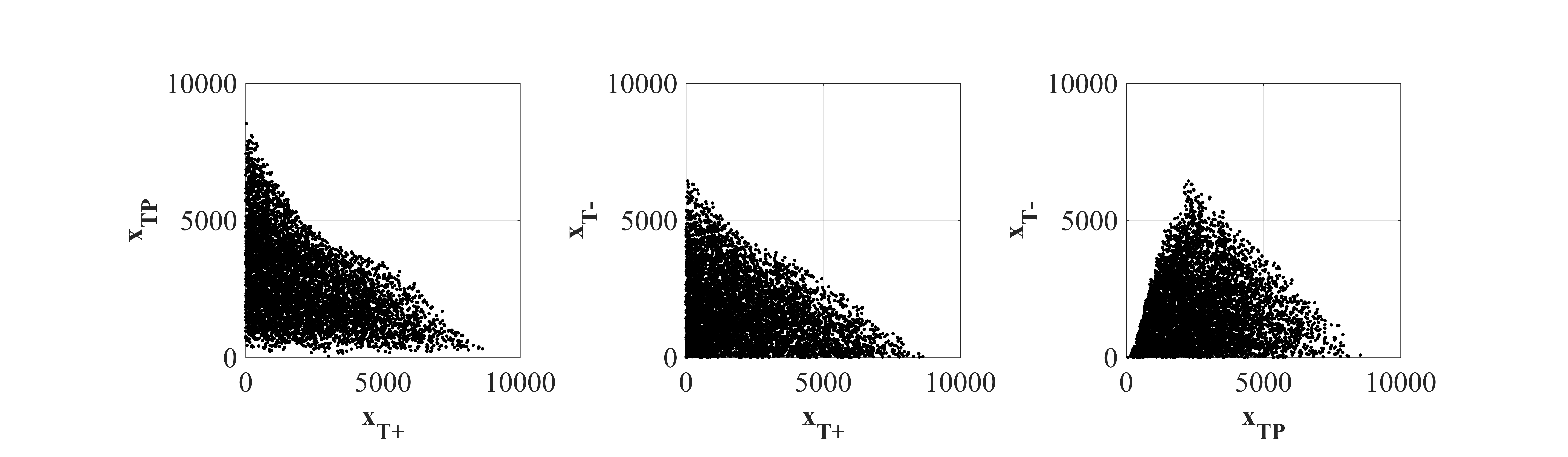

Supplement: S1 File — (ZIP) [file pone.0243386.s001.zip › SupportingInformation/FigureS27.tiff]

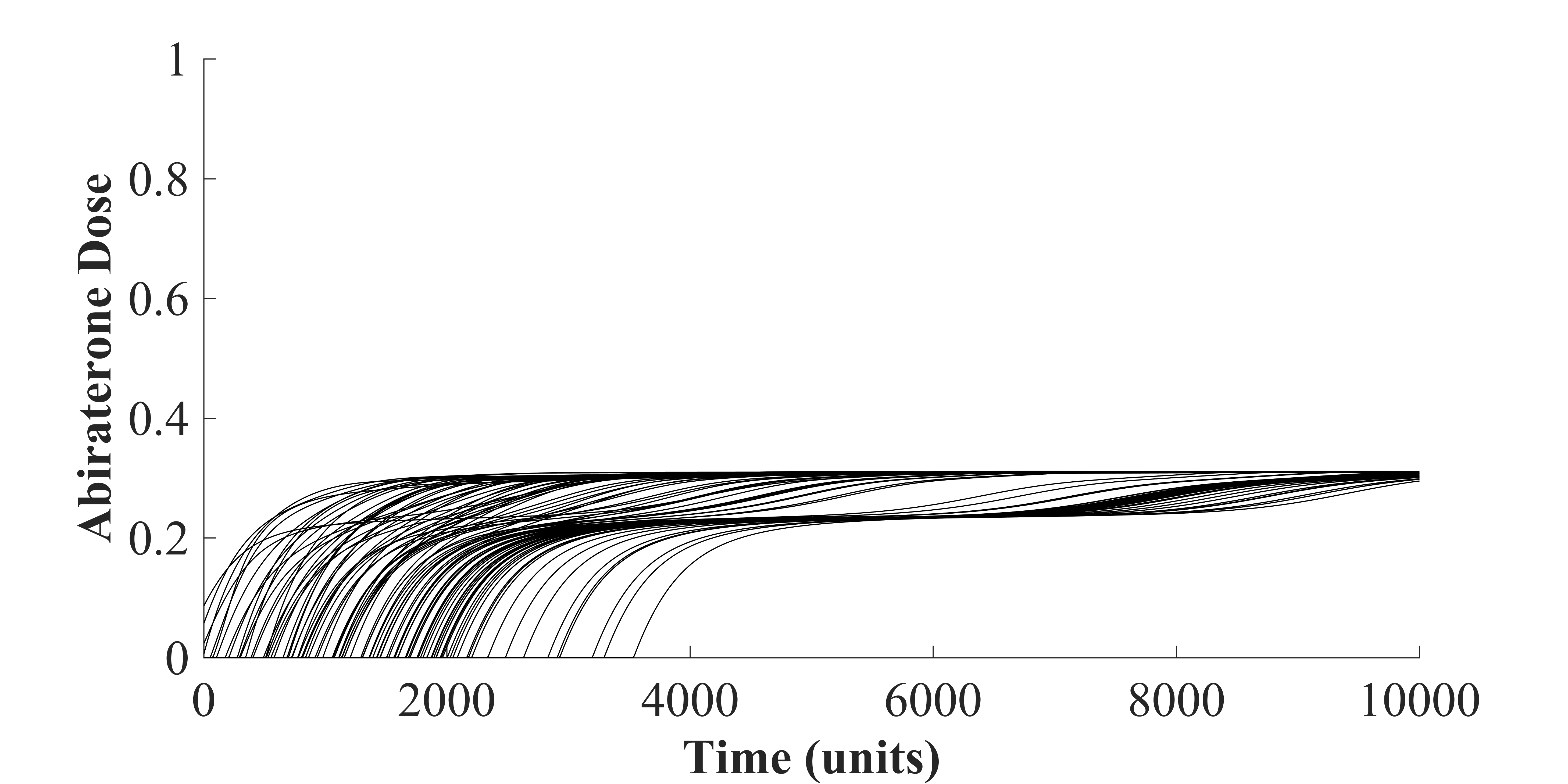

Supplement: S1 File — (ZIP) [file pone.0243386.s001.zip › SupportingInformation/FigureS11.tiff]

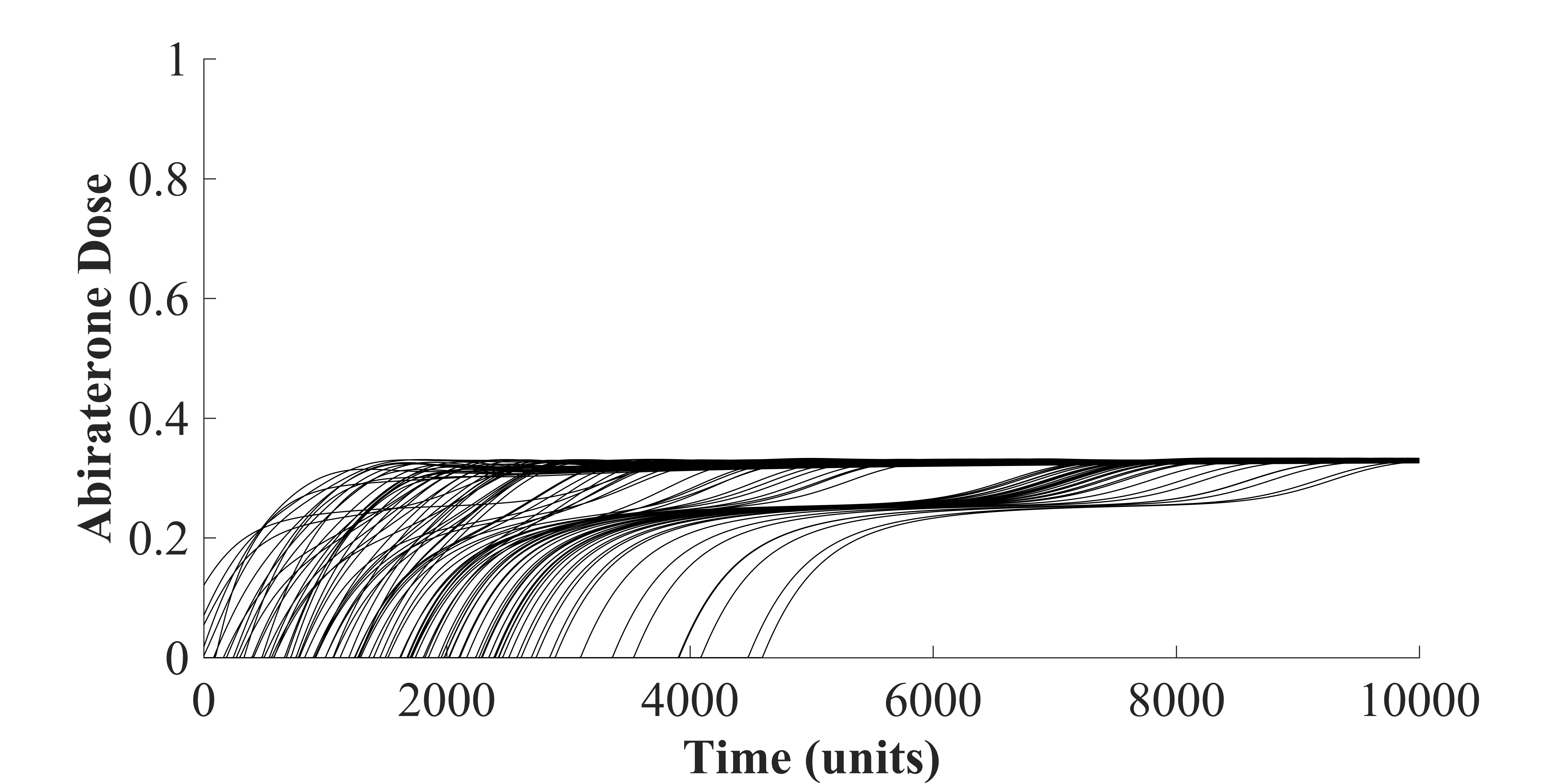

Supplement: S1 File — (ZIP) [file pone.0243386.s001.zip › SupportingInformation/FigureS12.tiff]

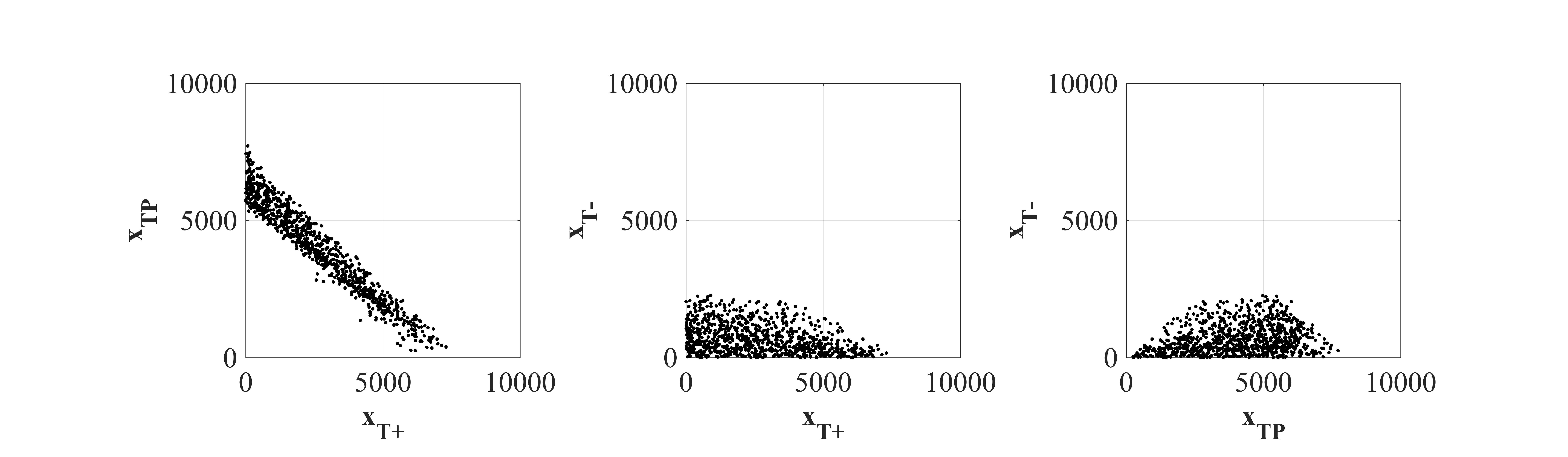

Supplement: S1 File — (ZIP) [file pone.0243386.s001.zip › SupportingInformation/FigureS24.tiff]

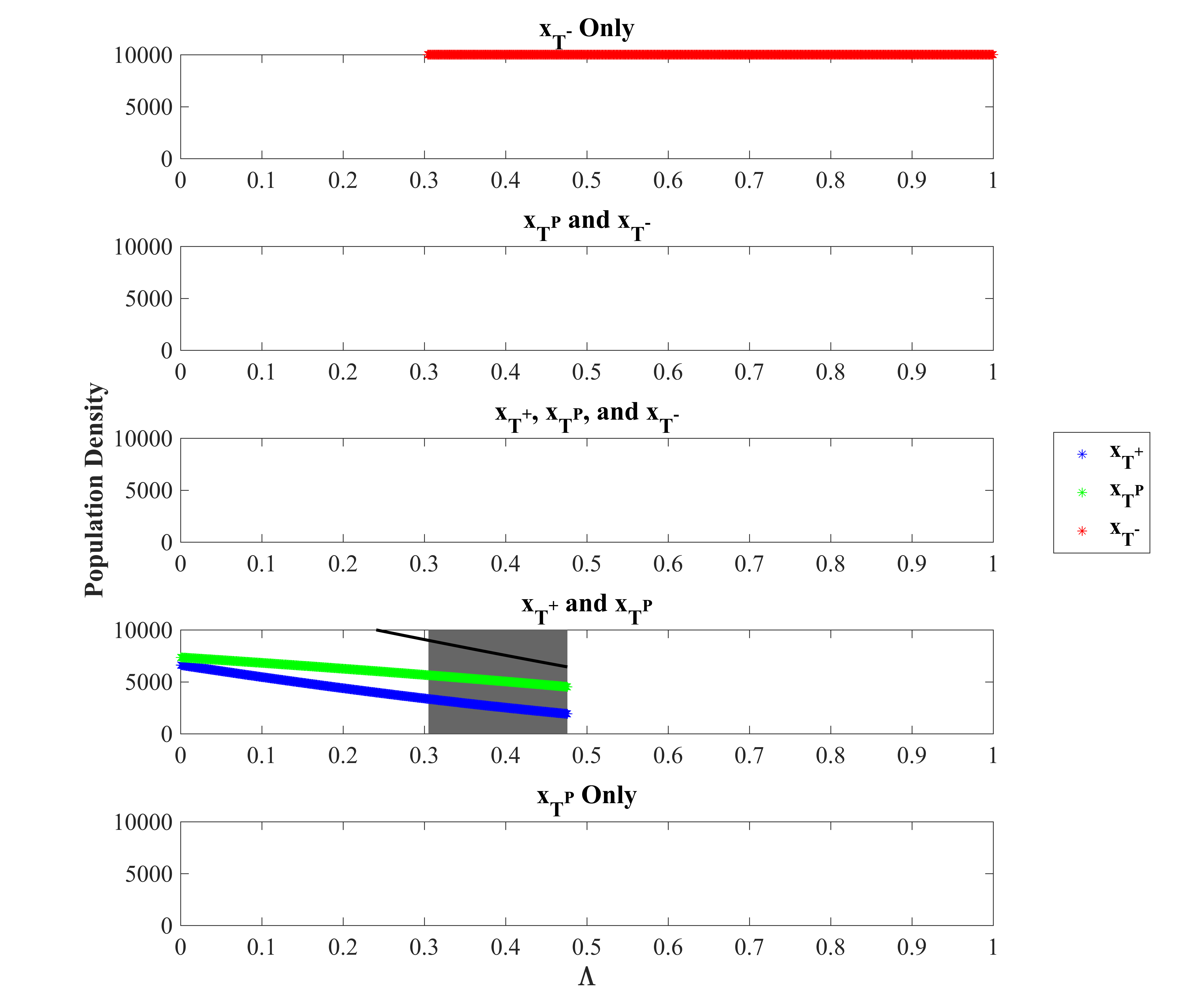

Supplement: S1 File — (ZIP) [file pone.0243386.s001.zip › SupportingInformation/FigureS3.tiff]

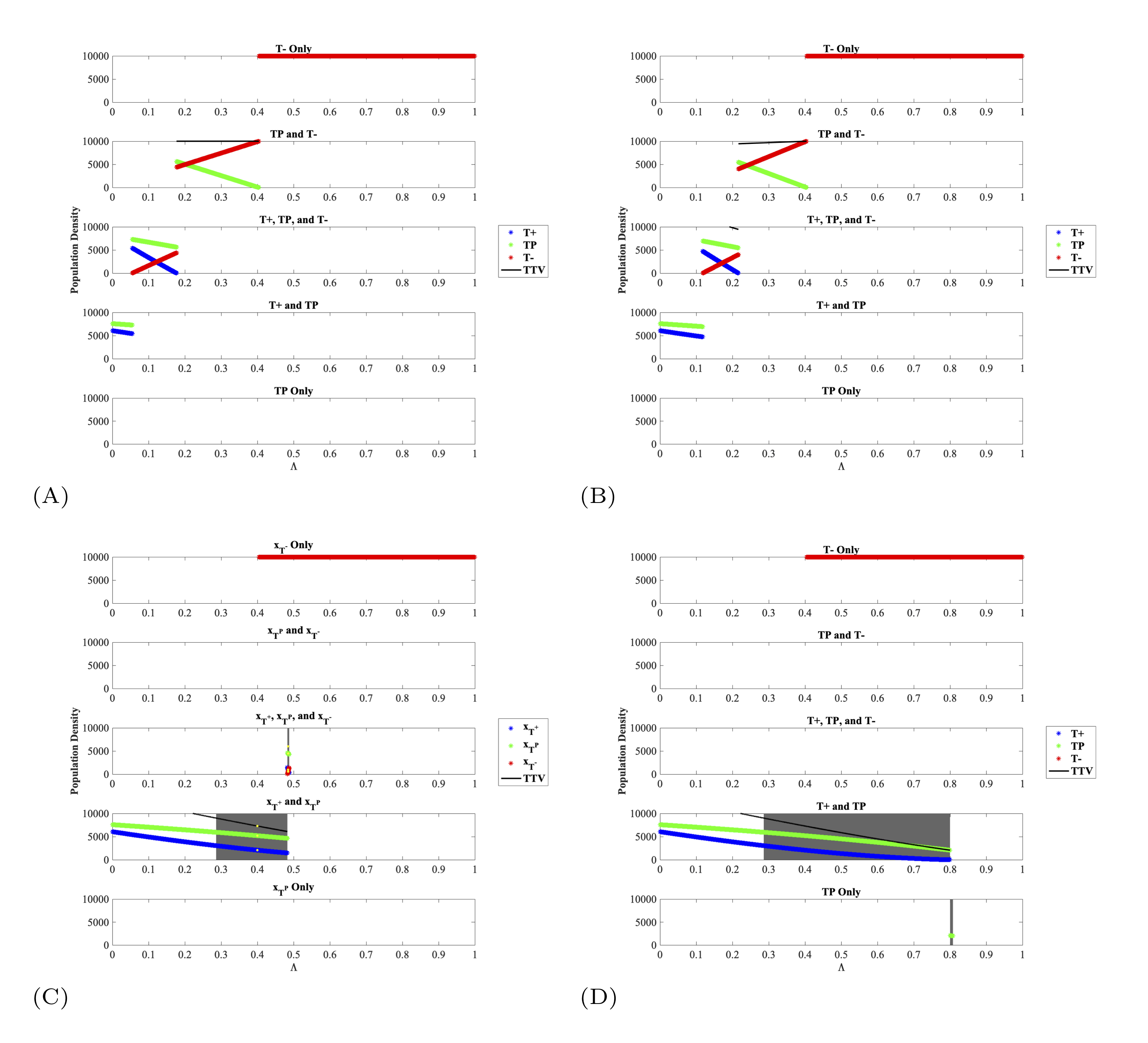

Supplement: S1 File — (ZIP) [file pone.0243386.s001.zip › SupportingInformation/FigureS2.tiff]

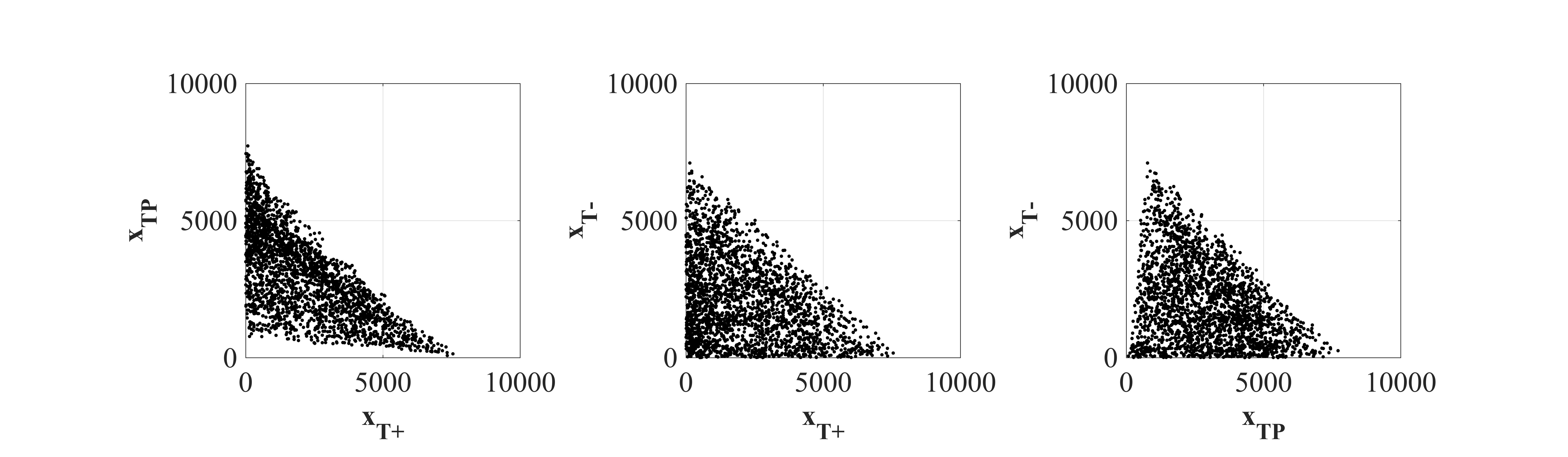

Supplement: S1 File — (ZIP) [file pone.0243386.s001.zip › SupportingInformation/FigureS25.tiff]

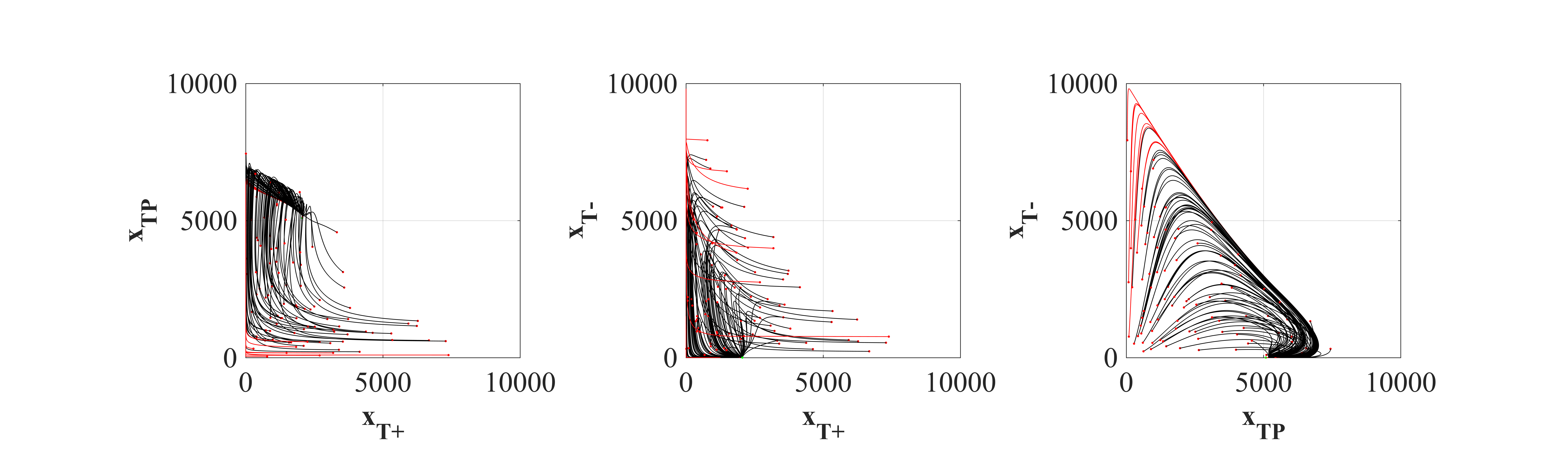

Supplement: S1 File — (ZIP) [file pone.0243386.s001.zip › SupportingInformation/FigureS13.tiff]

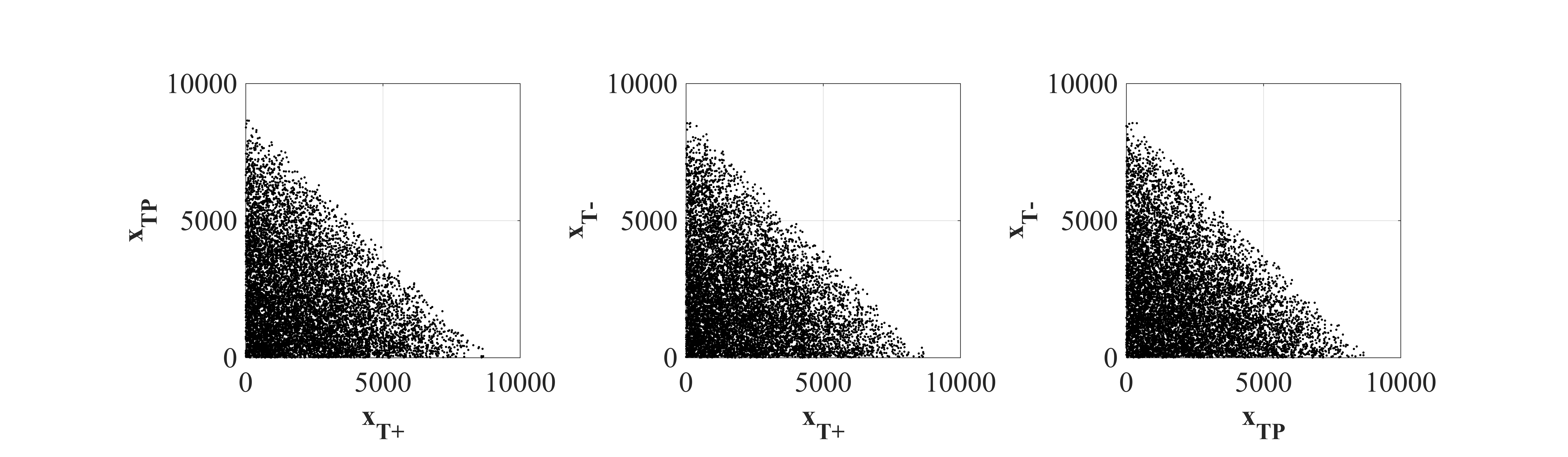

Supplement: S1 File — (ZIP) [file pone.0243386.s001.zip › SupportingInformation/FigureS22.tiff]

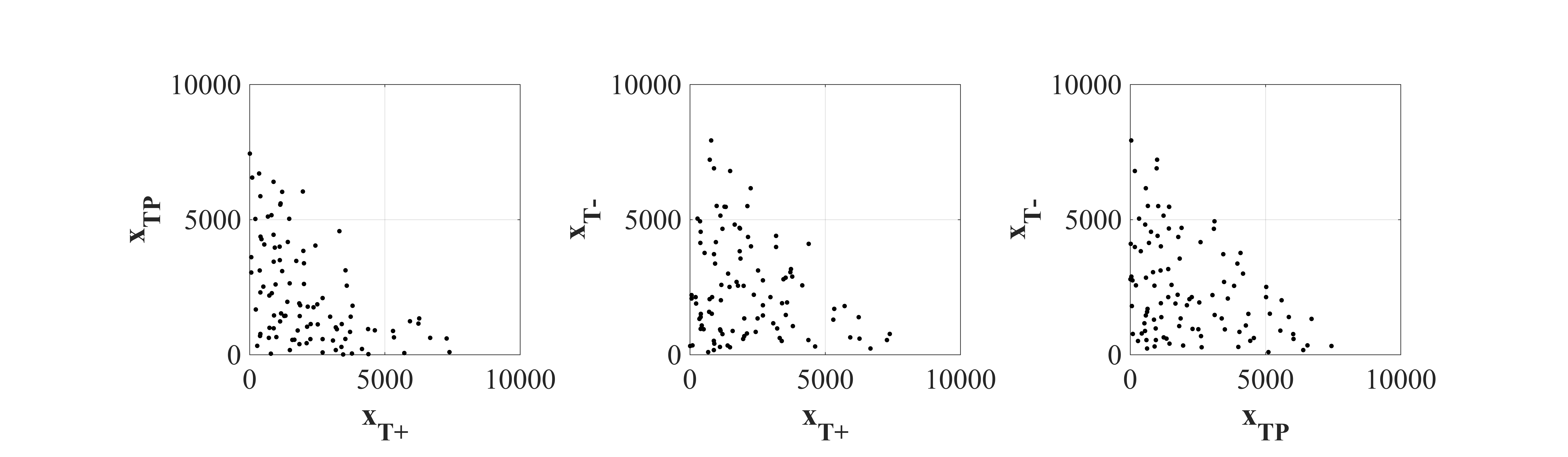

Supplement: S1 File — (ZIP) [file pone.0243386.s001.zip › SupportingInformation/FigureS5.tiff]

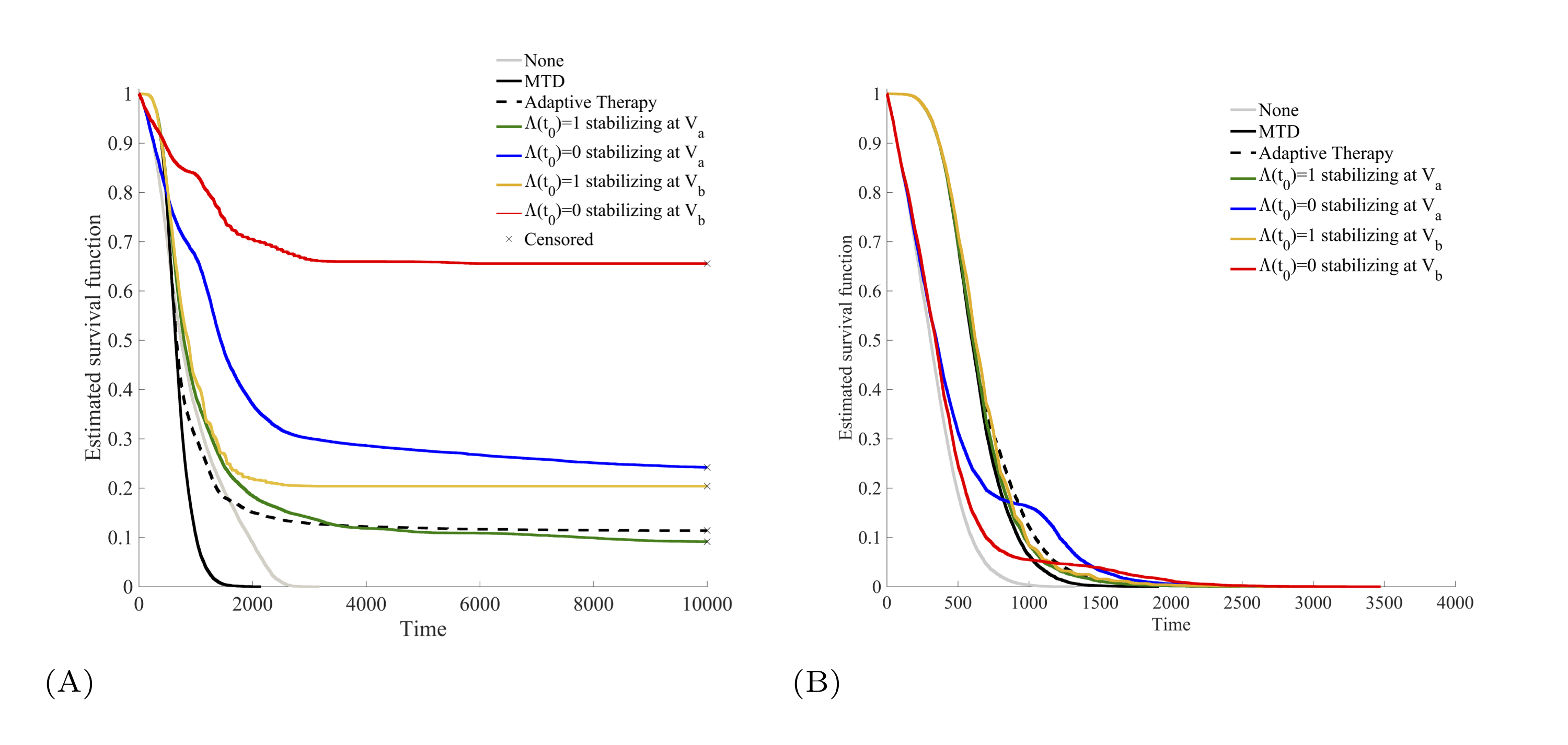

Supplement: S1 File — (ZIP) [file pone.0243386.s001.zip › SupportingInformation/FigureS18.tiff]

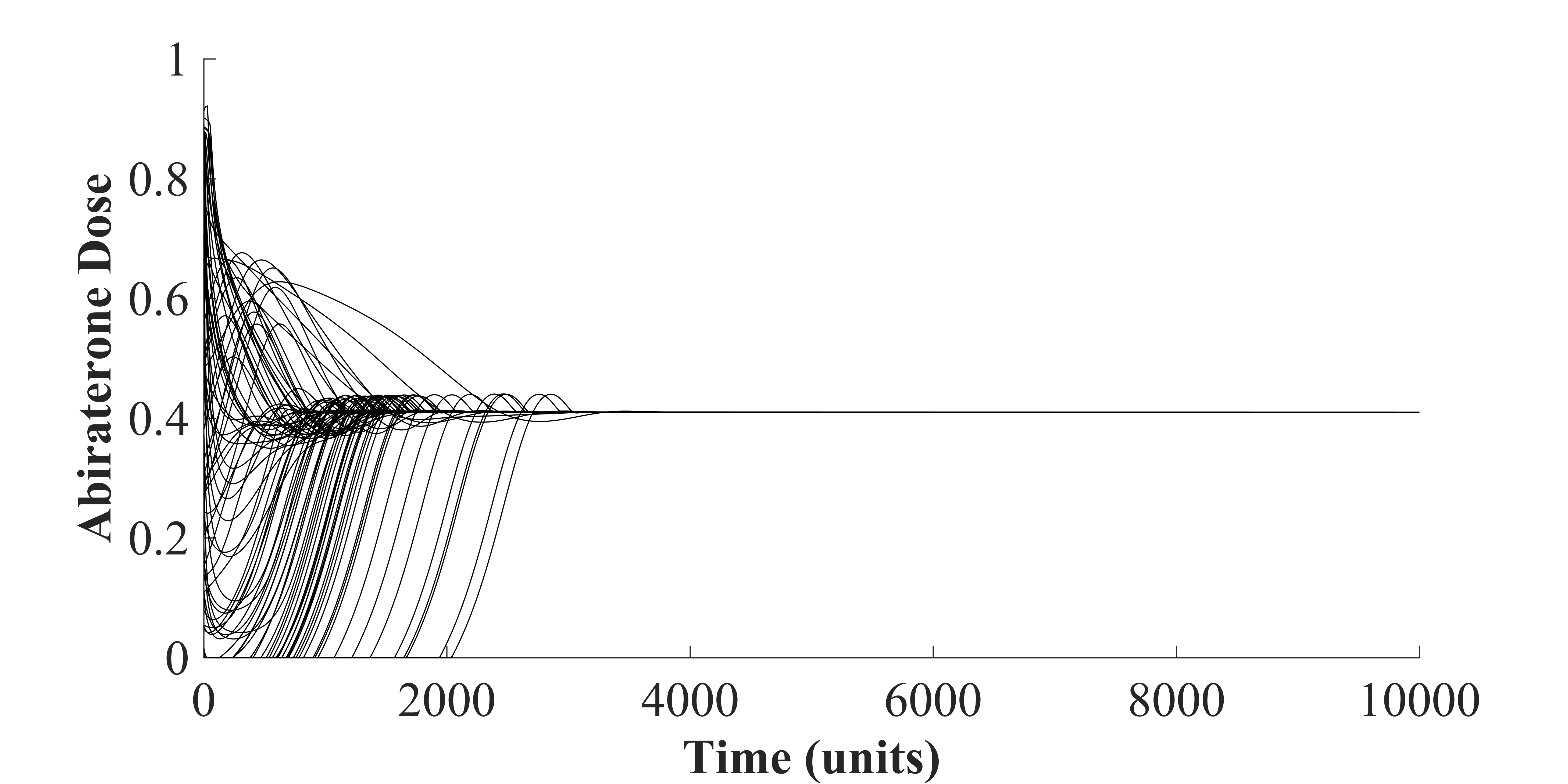

Supplement: S1 File — (ZIP) [file pone.0243386.s001.zip › SupportingInformation/FigureS9.tiff]

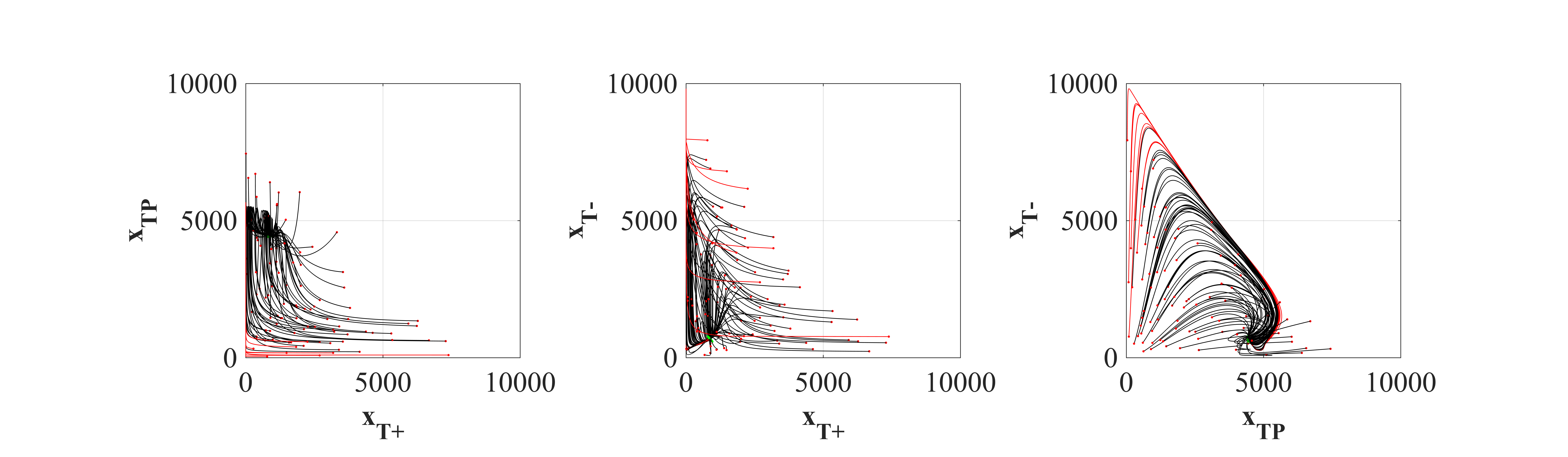

Supplement: S1 File — (ZIP) [file pone.0243386.s001.zip › SupportingInformation/FigureS14.tiff]

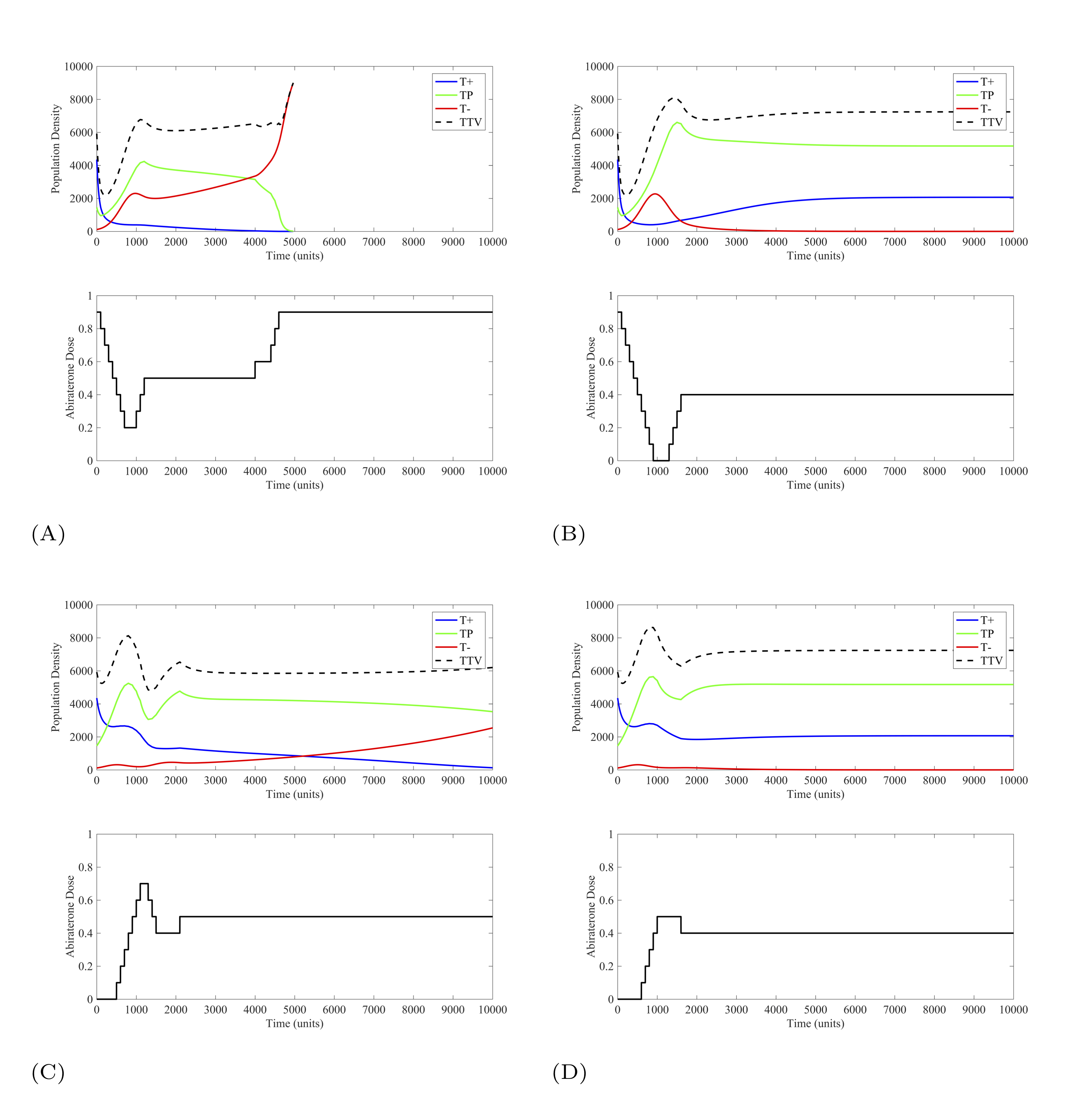

Supplement: S1 File — (ZIP) [file pone.0243386.s001.zip › SupportingInformation/FigureS15.tiff]

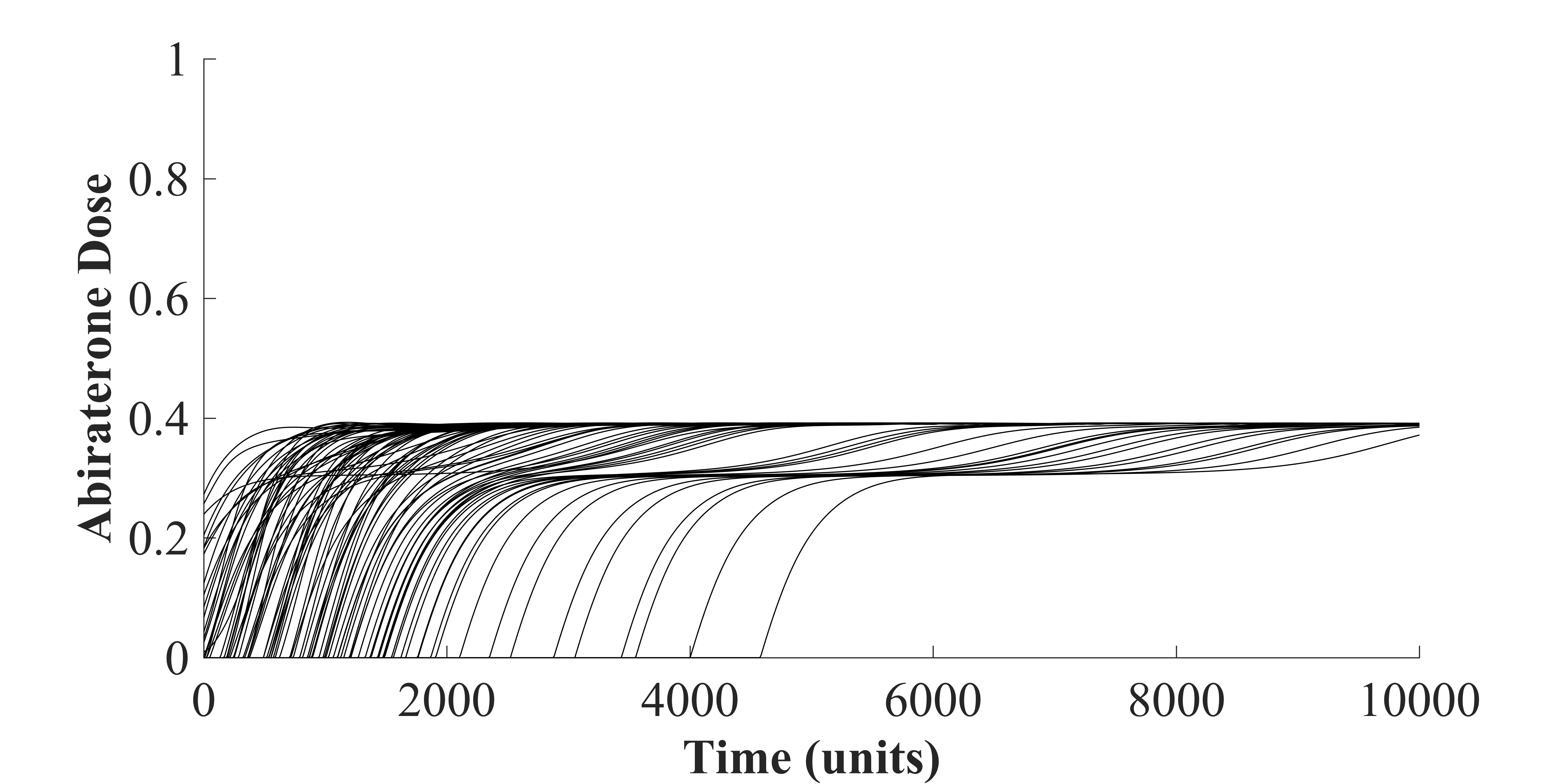

Supplement: S1 File — (ZIP) [file pone.0243386.s001.zip › SupportingInformation/FigureS8.tiff]

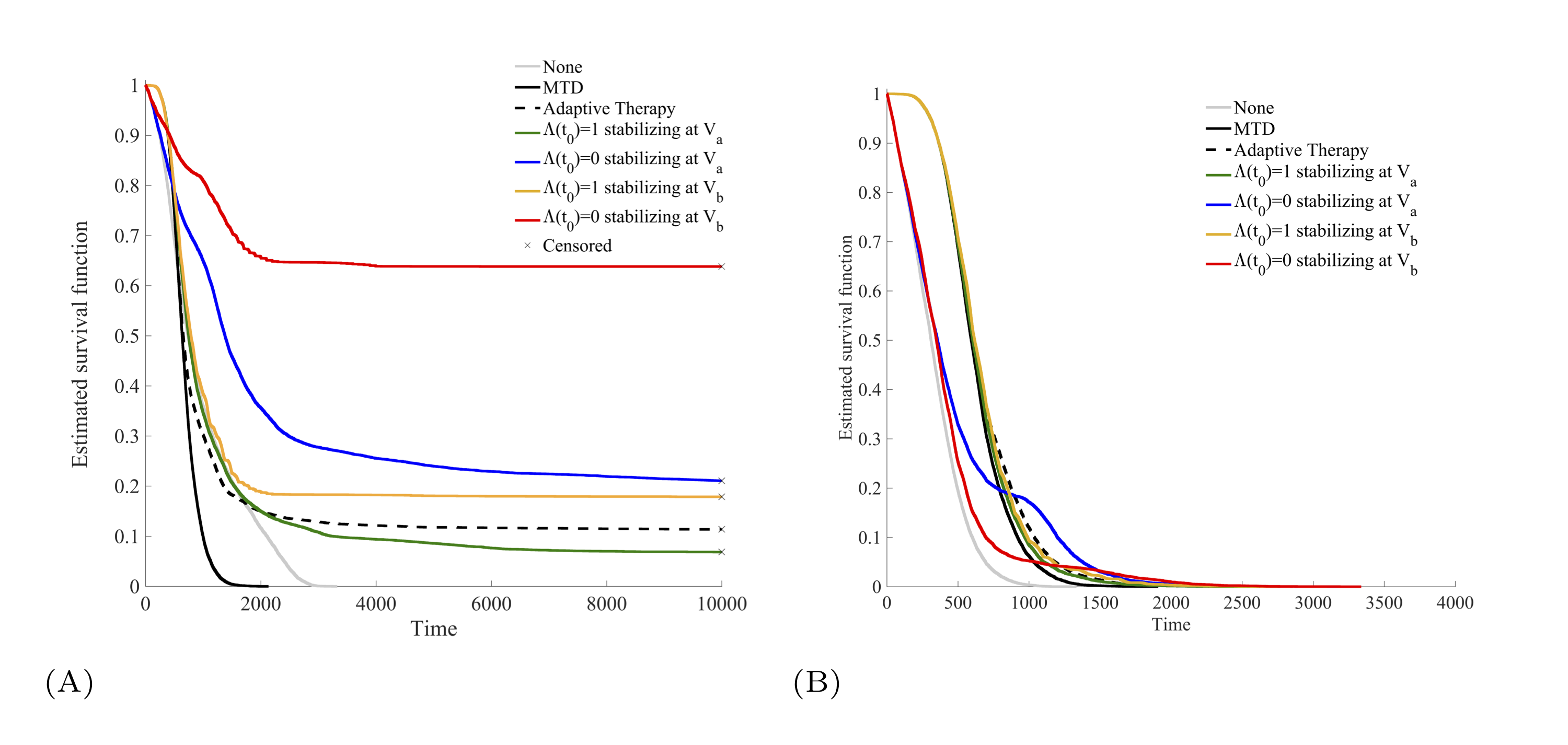

Supplement: S1 File — (ZIP) [file pone.0243386.s001.zip › SupportingInformation/FigureS19.tiff]

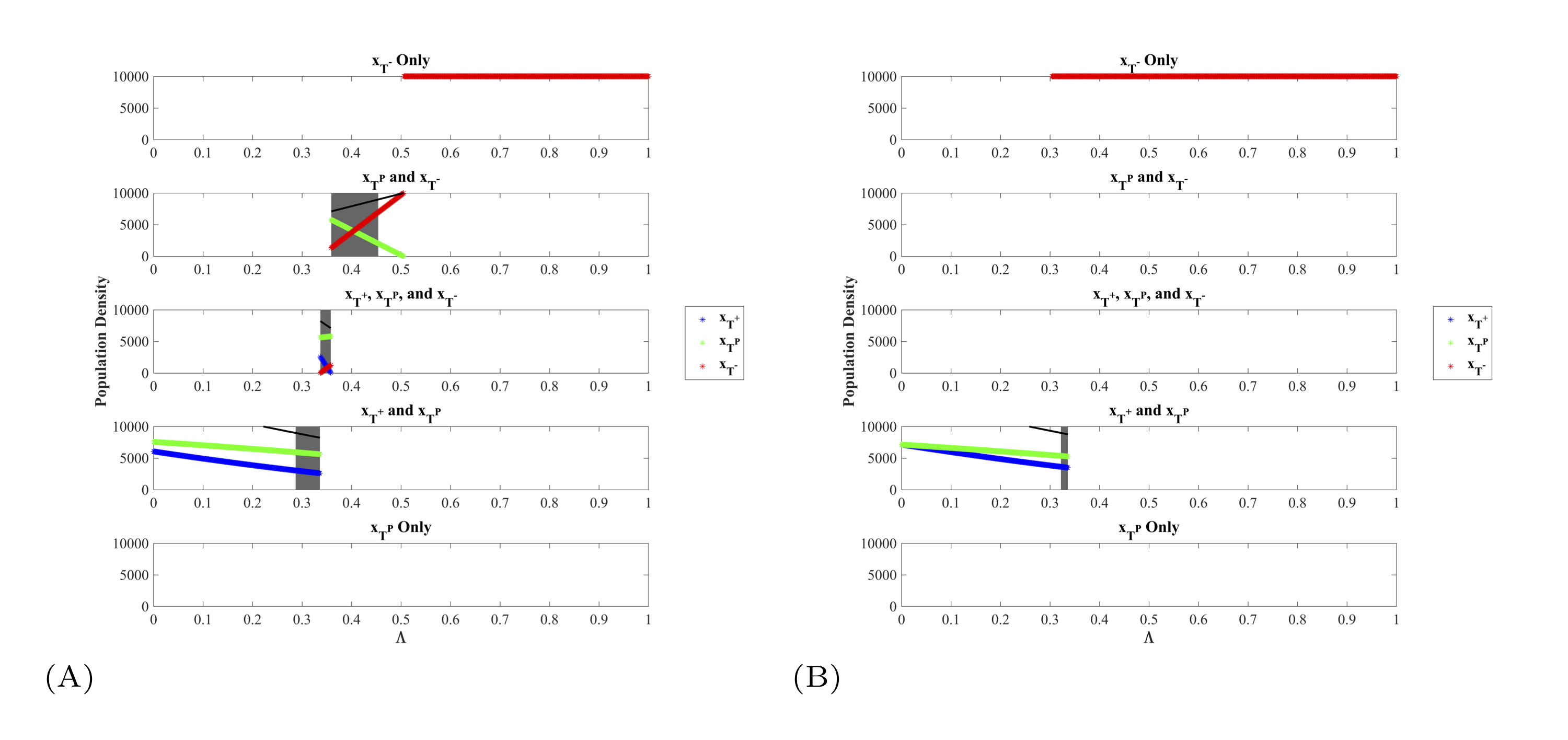

Supplement: S1 File — (ZIP) [file pone.0243386.s001.zip › SupportingInformation/FigureS4.tiff]

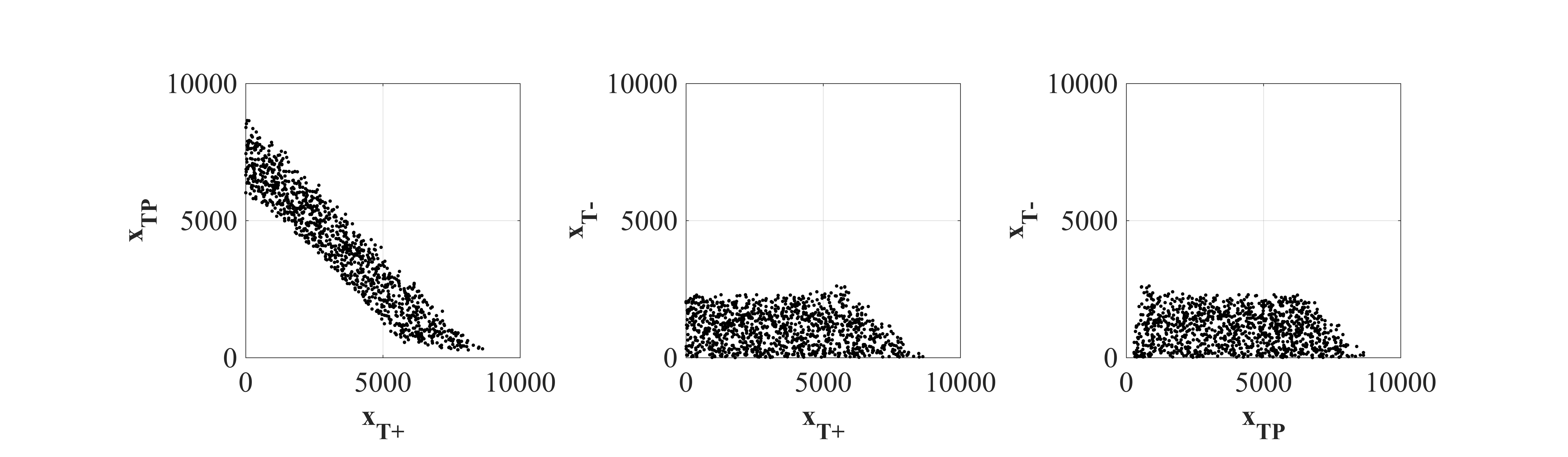

Supplement: S1 File — (ZIP) [file pone.0243386.s001.zip › SupportingInformation/FigureS23.tiff]
